# FKBP12 determines ligand-specific SMAD signaling in individual living endothelial cells

**DOI:** 10.1016/j.isci.2026.117728

**Published:** 2026-09-22

**Authors:** Zixin Huang, Sonja Lenhardt, Petra Snyder, Ahmed Bulldan, Felix Hausch, Alexander Loewer

**Affiliations:** 1 Department of Biology, Systems Biology of the Stress Response, Technical University Darmstadt, Darmstadt, Germany; 2 Department of Chemistry, Technical University Darmstadt, Darmstadt, Germany; 3 Centre for Synthetic Biology, Technical University Darmstadt, Darmstadt, Germany

**Keywords:** Biochemistry, cell biology

## Abstract

The BMP signaling pathway plays a pivotal role in regulating the formation and maintenance of our vascular system, employing a diverse set of ligands, receptors, and cytoplasmic mediators. However, little is known about how the interplay of these elements is regulated dynamically in individual living endothelial cells. Here, we developed fluorescent reporter to quantify BMP signaling and used a combination of time-resolved microscopy and computer-aided analysis to demonstrate ligand-specific dynamics of SMAD1, SMAD5, and SMAD9. Using this approach, we identified FKBP12 as a critical regulator of receptor complex activity that determines the balance of SMAD1/5/9 and SMAD2/3 signaling upon treatment with angiogenic ligands such as BMP7 and Activin A. This provides insights into the regulation of the BMP pathway in endothelial cells and offers a potential node for modulating vascular pathologies where the SMAD1/5/9-SMAD2/3 balance is disrupted.

## Highlights

• Fluorescent reporters reveal ligand-specific SMAD dynamics in endothelial cells
• BMP9 and BMP10 induce robust SMAD1/5/9 signaling via ALK1 activity
• FKBP12 restricts ALK2-mediated SMAD1/5/9 activation by BMP7 and Activin A
• FKBP12 controls the balance between SMAD1/5/9 and SMAD2/3 signaling

## Graphical abstract

**Figure:**
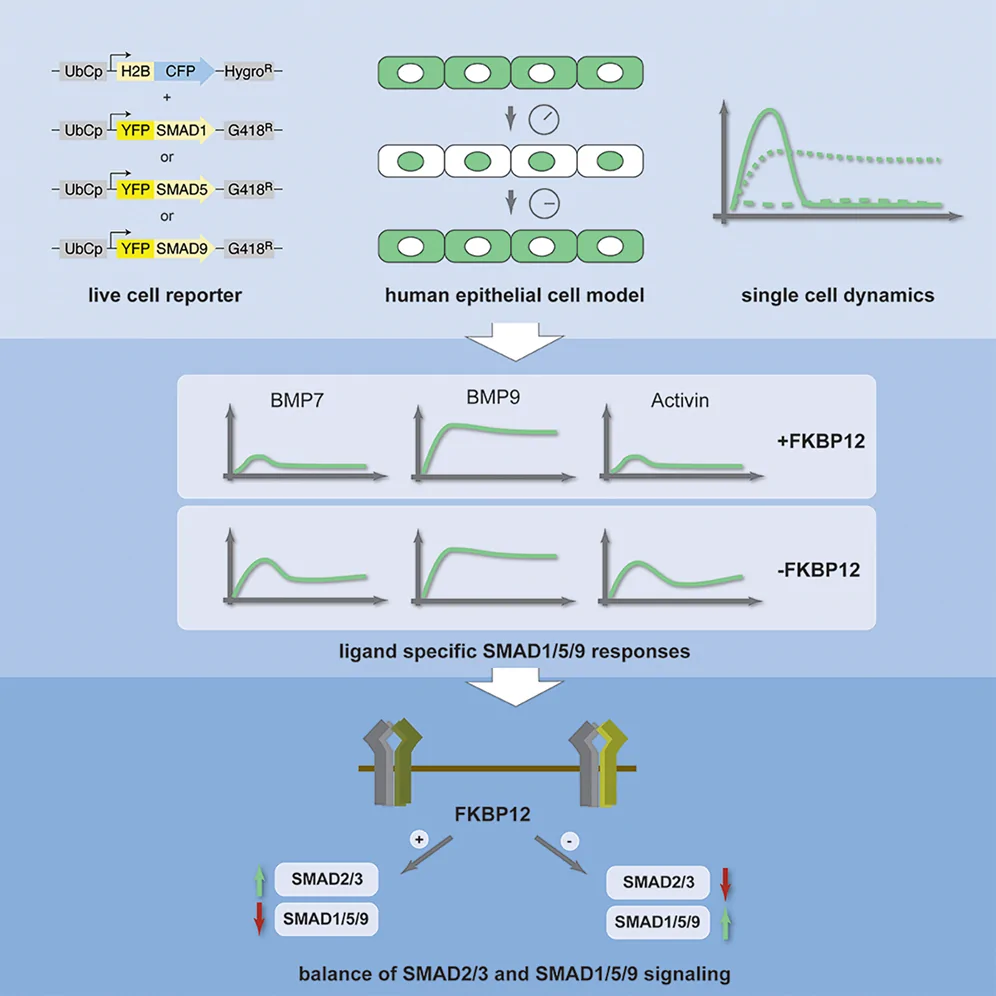

## Introduction

Our vascular system comprises an intricate network of vessels that ensure sufficient supplies of nutrients and respiratory gases to our tissues. As the vascular system needs to adapt to varying needs during development and tissue homeostasis, it retains the ability to remodel by forming new or modifying existing vessels. This vascular remodeling is governed by signaling processes including the SMAD pathway, which conveys both pro- and antiangiogenic signals to endothelial cells that line our vasculature.^1^ While individual functions of ligands have been revealed, we still lack an understanding of how the balance between different signaling activities allows individual cells to faithfully determine appropriate cell fates over time.

The SMAD signaling cascade is activated by the tumor growth factor (TGF)-β superfamily of ligands, which bind to serine/threonine kinase receptors on the cell surface.^2^ Upon ligand binding, type II receptors phosphorylate and activate type I receptors, which then phosphorylate receptor associated SMAD proteins (R-SMAD). These phosphorylated R-SMADs form complexes with a common SMAD protein (SMAD4) and translocate to the nucleus, where they regulate the transcription of target genes to orchestrate cellular responses to external stimuli. The SMAD pathway comprises more than 30 ligands, a range of type I and type II receptors, and five different R-SMADs.^3^ It can be divided into the TGF-β and bone morphogenetic protein (BMP) branches. The TGF-β branch, which includes TGF-β ligands, Activins, and growth differentiation factors (GDF), primarily utilizes the type I receptors activin receptor-like kinase 4 (ALK4, ACVR1B) and ALK5 (TGFBR1) with the type II receptor TGFBR2 to activate SMAD2 and SMAD3. The BMP branch engages the type I receptors ALK1 (ACVRL1), ALK2 (ACVR1), and ALK3 (BMPR1A), along with the type II receptors BMPR2, ACVR2A, and ACVR2B to activate SMAD1, SMAD5, and SMAD9. Co-receptors such as endoglin (ENG) for the BMP and betaglycan (TGFBR3) for the TGF-β branch further modulate signaling in endothelial cells.^4^^,^^5^

Ligands of the SMAD pathway play crucial roles across various stages of vascular development. During angiogenesis, tip cells at the leading edge of the sprouting vessels rely on SMAD1/5/9 activation by BMP2/6/7 for navigating and establishing new branches in the growing vessel network.^6^ Stalk cells—which follow the tip cells and contribute to forming the vessel lumen—are regulated by the SMAD2/3 pathways through TGF-β. This ensures proper vessel maturation and stability, facilitating cell proliferation and maintaining the structural integrity of the developing vessels.^7^ Finally, in mature vessels signaling via SMAD1/5/9 ensures that endothelial cells receive the appropriate inputs to maintain vascular quiescence and homeostasis. BMP9 and BMP10 are key ligands in this process.^5^ The role of Activin A is less clear, as it was described to both increase tubulogenesis and inhibit endothelial cell growth.^8^^,^^9^

The initial binding of ligand and receptor complexes is a critical determinant of signaling outcomes.^10^ Some ligands bind to defined complexes of type I and type II receptor dimers, while others are more promiscuous in their binding.^11^ BMP9 and BMP10, for example, bind almost exclusively to complexes of ALK1 and BMPRII,^12^ while BMP7 has the highest affinity to ALK2 in complex with BMPRII or ACTRIIA/B.^13^ Activin A, in contrast, was shown to signal both via ALK4 and ALK2 depending on the cellular context.^14^ The occurrence of mixed receptor complexes adds complexity to the pathway. Furthermore, intracellular regulators influence receptor-mediated SMAD phosphorylation. For example, it was reported that the immunophilin FK506-binding protein 12 (FKBP12) regulates type I receptors by binding to their glycine-serine-rich (GS) region and preventing their activation by type II receptors.^15^^,^^16^ Feedback by inhibitory SMAD proteins such as SMAD6 interferes with signal transduction at multiple levels.^17^ Collectively, these elements shape the dynamics of SMAD activity and influence endothelial cell fate decisions.

Dysregulation of the SMAD pathway can cause human vascular diseases: mutations in the BMP9 receptor ALK1 (ACVRL1) or its co-receptor ENG, for instance, attenuate SMAD1/5/9 signaling and cause hereditary hemorrhagic telangiectasia (HHT), which is characterized by arterio-venal malformations.^18^ Increased activity of SMAD2/3 by BMPR2 mutations leads to pulmonary arterial hypertension (PAH), where blood vessels narrow and remodel abnormally.^19^ This highlights that the right balance of SMAD signaling is critical for vascular homeostasis.^20^^,^^21^

In previous work, we demonstrated the importance of SMAD2 dynamics for regulating migration and survival in breast epithelial cells in a ligand and state-specific manner.^22^^,^^23^ We therefore hypothesized that the complex interplay of ligands and receptor complexes would result in specific dynamics of SMAD proteins in endothelial cells as well. In this context, we were specifically interested in investigating how both branches of SMAD signaling are balanced. We utilized a model for human vascular endothelial cells to generate fluorescent reporters and quantify SMAD dynamics over long time periods by live-cell microscopy and automated image analysis. Using pharmacological perturbations, we investigated the role of ligands and receptor complexes and reveal a regulatory function of FKBP12 in determining the balance of the different branches of the BMP/SMAD signaling pathway. We finally connect SMAD dynamics to target gene regulation and cell fate. Our insights in the regulatory logic governing this critical pathway will provide starting points for new therapeutic strategies to counteract human vascular diseases.

## Results

### Fluorescent reporters reveal SMAD dynamics upon BMP treatment

To systematically understand SMAD dynamics and monitor pathway activation on the single cell level, we developed stable clonal reporter for SMAD1 activation in EA.hy926 cells. EA.hy926 are a frequently employed model for human vascular endothelial cells generated by fusing primary human umbilical vein cells with the lung carcinoma cell line A549.^24^ The construction of reporter cells involved expressing an N-terminal fusion of the SMAD1 cDNA with the yellow fluorescent protein mVenus (YFP) under the control of a constitutive promoter (UbCp), similar to previously reported fusion constructs of SMAD2.^22^^,^^25^ Additionally, cells were engineered to stably express the nuclear marker histone H2B-CFP for segmentation and tracking of cells by computer-aided image analysis (Figure 1A).^22^ We selected a suitable clone based on expression levels and validated that both endogenous SMAD1/5/9 and YFP-SMAD1 showed similar phosphorylation patterns upon BMP9 stimulation in wild-type and reporter cell lines (Figures 1B, see Figures S1B–S1E for quantification). Specifically, phosphorylation levels peaked at approximately 1.5 h post-stimulation, followed by a gradual decrease over time. For these and all subsequent experiments, we reduced serum levels to 1.5% 20 h before stimulation to deplete any pre-existing ligands of the BMP family from the medium. Furthermore, dose-response analysis demonstrated that phosphorylation levels of phosphorylated SMAD1/5/9 and YFP-SMAD1 correlated with the concentration of the ligand. To corroborate our findings, we examined the induction of the well-characterized SMAD1/5/9 target genes Endothelin 1 (EDN1) and inhibitor of DNA binding 2 (ID2) by quantitative reverse-transcription PCR (RT-qPCR) (Figure 1C) and revealed similar expression levels between wild-type and reporter cell lines. This highlights that expression of fluorescently tagged SMAD1 did not severely perturb downstream processes of the signaling pathway. To further elucidate the dynamics of the SMAD1/5/9 pathway and to facilitate a comparative analysis, we generated similar fluorescent reporter for SMAD5 and SMAD9 and validated them by western blot and RT-qPCR as described (Figures S2 and S3). Please note that expression of YFP-SMAD5 led to slightly increased expression of target genes both in basal condition and upon stimulation.

**Figure 1:**
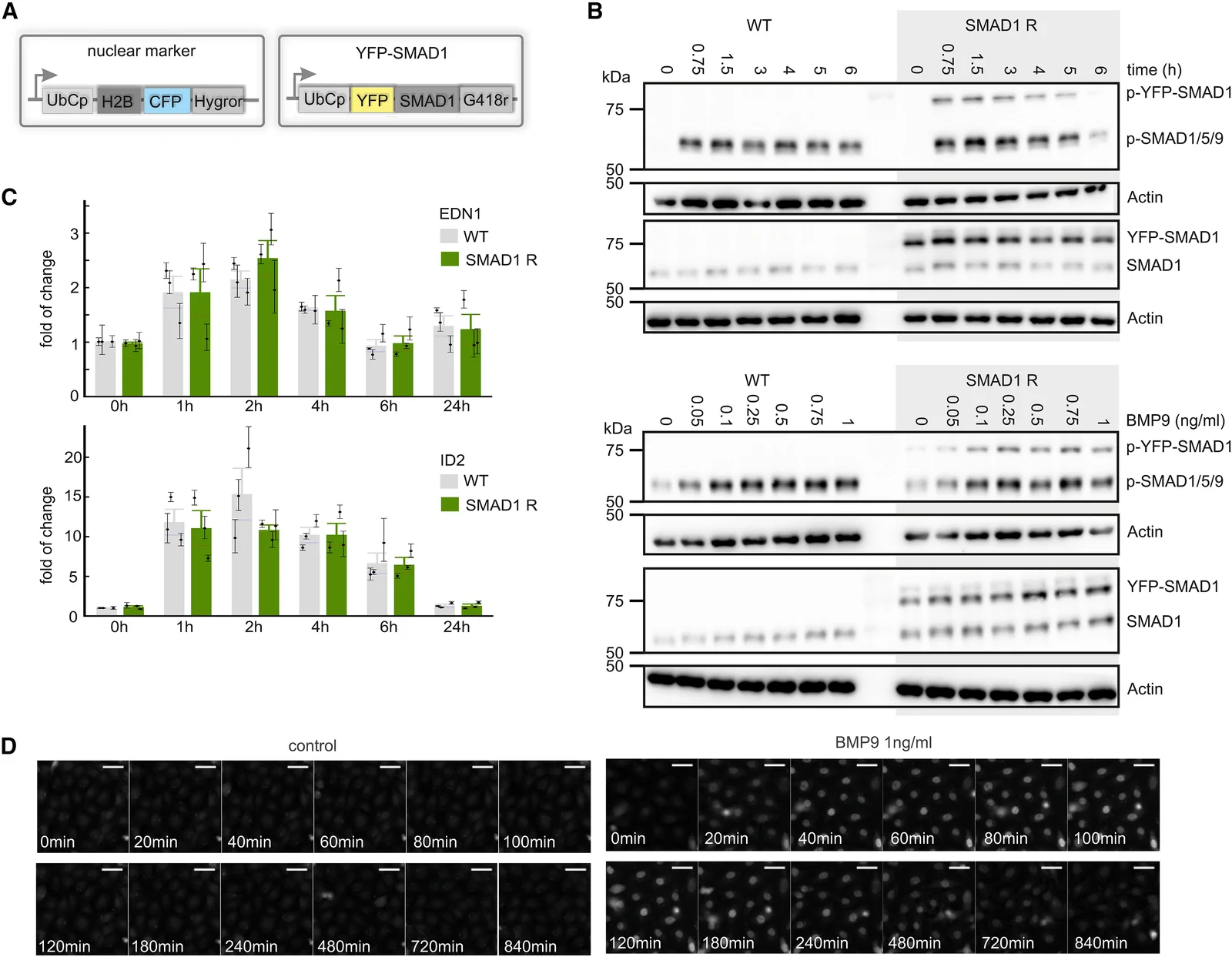
Selection of SMAD1 reporter clone and validation of SMAD1 dynamics (A) Scheme of the fluorescent reporter system. (B) Western blot analysis of SMAD1 activation in SMAD1 reporter cells and parental EA.hy926 wild-type cells. Phosphorylated SMAD1/5/9 and total SMAD1 were examined at indicated time points after stimulation with 1 ng/mL BMP9 (top) as well as 1.5 h after stimulation with varying doses of the ligand (bottom). Actin served as loading control. Representative blots from three independent experiments are shown. (C) RT-qPCR analysis of SMAD1 target gene expression in EA.hy926 WT and EA.hy926 SMAD1 reporter cells. Cells were treated with 1 ng/mL BMP9 and samples harvested at indicated time points. Fold changes were calculated in relation to unstimulated cells and β-actin as a reference. Colored bars represent mean ± standard deviation (SD) of biological triplicates, individual data points ± standard deviation (SD) of technical triplicates are shown in black. (D) Representative images from live-cell microscopy of reporter cells under control conditions or upon treatment with 1 ng/mL BMP9. Scale bars: 50 μm. Representative images from three independent experiments are shown. For (B)–(D), cells were pre-cultured in 1.5% FBS medium for 20 h prior to BMP9 stimulation.

Next, we employed our reporter to investigate the intracellular localization dynamics of SMADs in living cells using time-lapse microscopy. SMAD1 reporter cells were treated with 1 ng/mL BMP9 and imaged at 10-min intervals over a 16-h period (Figure 1D). As expected, we observed a robust nuclear translocation of SMAD1 about 1-h post-stimulation, followed by a gradual return to the cytoplasm.

To quantify SMAD dynamics, we measured fluorescence intensities both in the nucleus and cytoplasm using computer-aided image analysis and generated trajectories of the nuclear to cytoplasmic (nuc/cyt) SMAD ratio as a surrogate for pathway activation in individual cells (Figure 2A, see STAR Methods for details). Interestingly, we observed significant heterogeneity of the response. In general, cells exhibited nuclear accumulation of SMAD1 shortly after the initial stimulus. However, while some cells displayed relatively stable signaling afterward (e.g., cell 3 and 5), others demonstrated repeated nuclear translocation of SMAD1 (e.g., cell 2 and 6). This is consistent with previously reported heterogeneity of SMAD2 translocation in breast epithelial cells, which was mainly attributed to varying levels of signaling molecules such as receptors or feedback regulators.^22^ Varying cell-cycle states could contribute to the observed variability as well (Figures S4A and S4B). The median nuc/cyt SMAD1 ratio for the cell population showed an initial strong peak of SMAD1 translocation approximately 1.5 h post-stimulation, followed by a gradual return to basal levels (Figure 2B). Notably, even a stimulation control containing only fresh medium without added ligand elicited a minor nuclear translocation peak, highlighting the sensitivity of SMAD1 to minimal ligand levels (Figure 2B). We observed similar dynamics of SMAD5 and SMAD9 upon BMP stimulation using the corresponding fluorescent reporter cell lines (Figures S4C and S4I). However, for SMAD5, the remaining ligands in the stimulation control did not suffice to induce a translocation peak, suggesting a lower sensitivity of this signaling mediator compared to SMAD1 and SMAD9.

**Figure 2:**
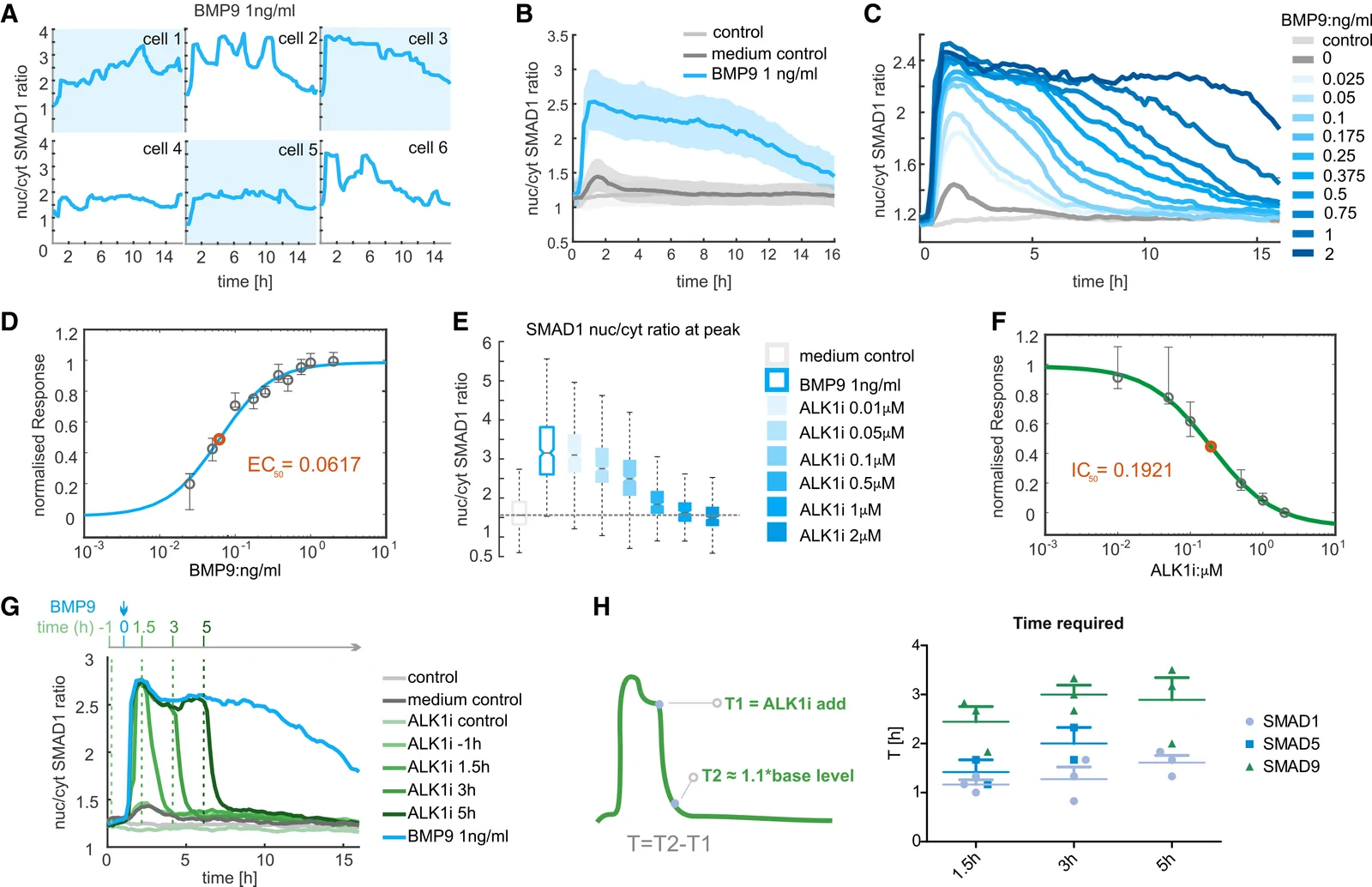
Dynamics of SMAD signaling in living cells (A) Nuc/cyt YFP-SMAD1 ratios for selected cells upon stimulation with 1 ng/mL BMP9. (B) Median nuc/cyt SMAD1 ratio in cell populations upon stimulation with 1 ng/mL BMP9 in fresh medium containing 1.5% FBS (blue), cells treated with fresh medium containing 1.5% FBS only (dark gray), or in unstimulated control populations (light gray). Solid lines represent median values; shaded areas indicate 25^th^ to 75^th^ percentiles. (C) Median nuc/cyt SMAD1 ratio following stimulation with indicated BMP9 concentrations. (D) Normalized SMAD1 response to increasing doses of BMP9 based on peak median nuc/cyt SMAD1 ratios. The half-maximal effective concentration (EC_50_ value) was calculated from the indicated fit (blue line and orange circle). Data represent mean ± SD from three independent experiments. (E) Peak nuc/cyt SMAD1 ratio in cells treated with various ALK1i (K02288) concentrations 1 h before stimulation with 1 ng/mL BMP9. Boxes include data between the 25^th^ and 75^th^ percentiles of the cell population, black lines indicate the median and whiskers extend to maximum values within 1.5× the interquartile range. Notches indicate 95% confidence intervals. Dashed line serves as guide to the eye. (F) Normalized SMAD1 response to increasing concentrations of ALK1i based on peak nuc/cyt SMAD1 ratios upon stimulation with 1 ng/ml BMP9. The half-maximal inhibitory concentration (IC_50_ value) was calculated from the indicated fit (blue line and orange circle). Data represent mean ± SD from three independent experiments. (G) ALK1 inhibitor efficacy at different timepoints relative to BMP9 induction. Cells were treated with 1 μM ALK1i 1 h before or 1.5, 3, 5 h after 1 ng/mL BMP9 stimulation. Lines indicate median nuc/cyt SMAD1 ratio in cell populations. (H) Time required to reduce nuc/cyt ratio from 90% to 10% for SMAD1 (lilac), SMAD5 (blue), and SMAD9 (green) upon 1 μM ALK1i addition at 1.5, 3, or 5 h after BMP9 stimulation. Symbols represent individual biological replicates; lines represent the mean. For all experiments, cells were pre-cultured in 1.5% FBS medium for 20 h prior to stimulation. Time-lapse imaging was performed every 10 min for 16 h, starting 0.5 h before stimulation, unless otherwise stated. See Table S1 for number cells analyzed in this and subsequent figures. Data are representative for at least three biological repeats if not stated otherwise.

To further investigate the sensitivity of cells to BMP9, we systematically exposed reporter cells to varying concentrations of BMP9 (Figure 2C). Increasing BMP9 doses resulted in elevated peak levels of SMAD1 translocation, saturating at approximately 0.375 ng/mL, with a half-maximal effective concentration (EC_50_) of 0.06 ng/mL (Figure 2D). Notably, higher BMP9 doses lead to sustained SMAD1 translocation and a delayed adaptation back to pre-stimulus levels (Figure 2C). As it has been previously shown that TGF-β family ligands are internalized with their receptors and degraded by the lysosome, leading to termination of signaling once ligands are depleted from the medium,^22^^,^^26^^,^^27^^,^^28^ our observation indicates that the duration of BMP9 signaling in endothelial cells is determined by ligand depletion as well. SMAD5 and SMAD9 exhibited similar dose-dependent responses (Figures S4D and S4J). However, both required higher concentrations to reach saturation with SMAD5 demonstrating the highest EC_50_ value among the three proteins (Figures S4E and S4K).

To validate the dependency of SMAD activation by BMP9 on ALK1, we treated reporter cells with the inhibitor K02288 (ALK1i) 1 h prior to stimulation. Live-cell microscopy experiments revealed a decrease in SMAD1 translocation into the nucleus with increasing concentrations of inhibitor (Figure 2E). The calculated half-maximal inhibitory concentration (IC_50_) of the peak nuc/cyt SMAD1 ratio was 192 nM (Figure 2F). Inhibition of SMAD5 and SMAD9 by ALK1i followed a similar pattern (Figures S4F and S4L) with overall lower IC_50_ values (SMAD5: 47 nM, SMAD9: 107 nM; Figures S4G and S4M). Importantly, sustained translocation of SMAD proteins was dependent on ALK1 activity at all time points (Figures 2G, S4H, and S4N). When we calculated the duration required for SMAD proteins to relocate to the cytoplasm upon inhibitor treatment, we observed that the SMAD9 nuc/cyt ratio returned more slowly to baseline compared to SMAD1 and SMAD5 (Figure 2H).

In summary, our comparative analysis of SMAD1, 5, and 9 translocations upon BMP9 stimulation revealed qualitatively similar dynamics but quantitatively differing sensitivity to receptor activity as well as kinetics of cytoplasmic relocation upon signal termination.

### FKBP12 modulates the activity of type I receptors in EA.hy926 cells

So far, we focused on BMP9-induced SMAD signaling in endothelial cells. Next, we aimed to gain a more comprehensive understanding of SMAD1/5/9-mediated responses to ligands of the BMP and TGF-β superfamily. To this end, we selected several ligands activating SMAD1/5/9 signaling, BMP2, BMP6, BMP7, BMP9, BMP10, as well as ligands known to activate the SMAD2/3 branch, such as TGF-β, GDF11, and Activin A.^3^ Previously published RNA-seq data confirmed expression of both type I and type II receptors in EA.hy926 cells, although at varying levels (Figures S5A–S5C).^29^ Initially, we employed western blot analysis and observed distinct patterns of SMAD1/5/9 phosphorylation upon ligand stimulation. Notably, only BMP9 and BMP10 elicited robust phosphorylation of SMAD1/5/9, with BMP2 and BMP7 inducing weaker signals (Figures 3A and S5F). For SMAD2, we observed robust phosphorylation upon TGF-β, GDF11, and Activin A treatment as expected. BMP9 and BMP10 induced weaker signals as previously reported for human endothelial cells.^30^ For SMAD3, the results upon BMP stimulation were difficult to interpret, as the presence of a band corresponding to exogenously expressed YFP-SMAD1 indicated cross-reactivity of the utilized phosphor-specific antibodies in EA.hy926 cells (Figure 3A). Such cross-reactivity was previously reported for human endothelial cells^30^ and corroborated by further analysis of a knockout cell line and the use of pharmacological inhibitors (Figure S5G).

**Figure 3:**
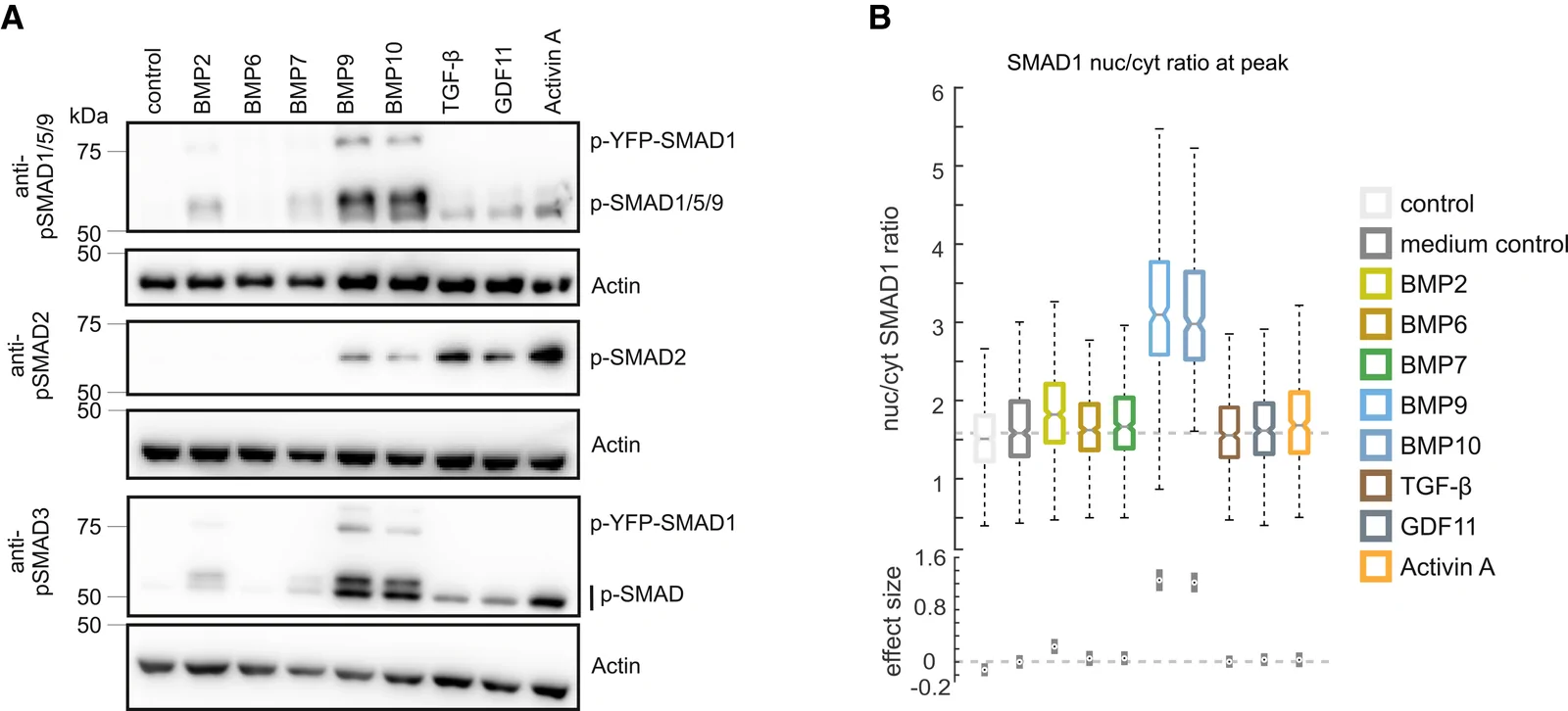
Different ligands induce specific SMAD responses (A) Western blot analysis of SMAD1/5/9 (top), SMAD2 (middle), and SMAD3 (bottom) phosphorylation in SMAD1 reporter cells 1.5 h after stimulation with different TGF-β superfamily ligands. Concentrations used were: BMP2 (250 ng/mL), BMP6 (250 ng/mL), BMP7 (250 ng/mL), BMP9 (1 ng/mL), BMP10 (1 ng/mL), TGF-β (50 ng/mL), GDF11 (250 ng/mL), and Activin A (250 ng/mL). Actin served as loading control. Representative blots from three independent experiments are shown. (B) Quantification of peak nuc/cyt SMAD1 ratio following stimulation with the indicated ligands. Ligand concentrations were identical to those used in (A). Upper part of the image: boxplots showing distribution of cellular responses. Lower part of the image: estimated changes in SMAD1 nuclear translocation compared to non-ligand stimulated cells. Error bars represent 90% confidence intervals determined by permutation testing. Dashed lines are provided as guides to the eye. Cells were cultured in 1.5% FBS medium for 20 h prior to stimulation. Time-lapse imaging was performed every 10 min for 16 h, starting 0.5 h before stimulation. Data are representative for three biological repeats.

We therefore turned to time-lapse microscopy of reporter cell lines to screen SMAD translocation upon stimulation with varying ligands. Remarkably, only cells treated with BMP9 and BMP10 exhibited robust translocation of SMAD1, SMAD5, and SMAD9 (Figures 3B, S5H, and S5I). The response to BMP2 and BMP7 further highlighted differences in sensitivity among the three proteins, with SMAD1 and SMAD9 displaying slightly elevated nuclear accumulation upon stimulation, while no response could be observed for SMAD5. Our observations from time-lapse microscopy were confirmed by qPCR-based analysis of target gene expression upon stimulation with BMP-family ligands (Figure S5J).

The differential response to BMP-family ligands indicates that EA.hy926 cells mainly respond to activation of ALK1. This could be caused by the comparably low expression of ALK2 and ALK3 (Figure S5A). Alternatively, we hypothesized that inhibitory factors impede the activity of the remaining receptors. As BMP9 and 10 elicited robust SMAD translocation, we excluded factors acting downstream of SMAD phosphorylation. Furthermore, expression analysis revealed low or absent expression of extracellular inhibitory proteins known to block SMAD signaling (Figure S5D).^15^ Moreover, the high concentration of externally supplied ligands is expected to saturate any remaining extracellular inhibitors. Therefore, we focused on factors that inhibit receptors themselves and observed that FKBP12 (encoded by FKBP1A gene), a known inhibitor of ALK1, ALK2, and ALK3,^31^ was highly expressed in EA.hy926 cells (Figure S5E). FKBP12 binds to type I receptors, obstructing signaling by occupying the phosphorylation site in the GS region.^15^ To test the involvement of FKBP12 in regulating the activity of type I receptors in endothelial cells, we employed a FKBP12 proteolysis targeting chimera (PROTAC), a bifunctional small molecule that recruits the ubiquitin ligase von Hippel-Lindau protein (VHL) to FKBP12 and thereby induces its degradation (Figure 4A).^32^ Western blot analysis confirmed dose- and time-dependent degradation of FKBP12 protein by the PROTAC (Figure 4B).

**Figure 4:**
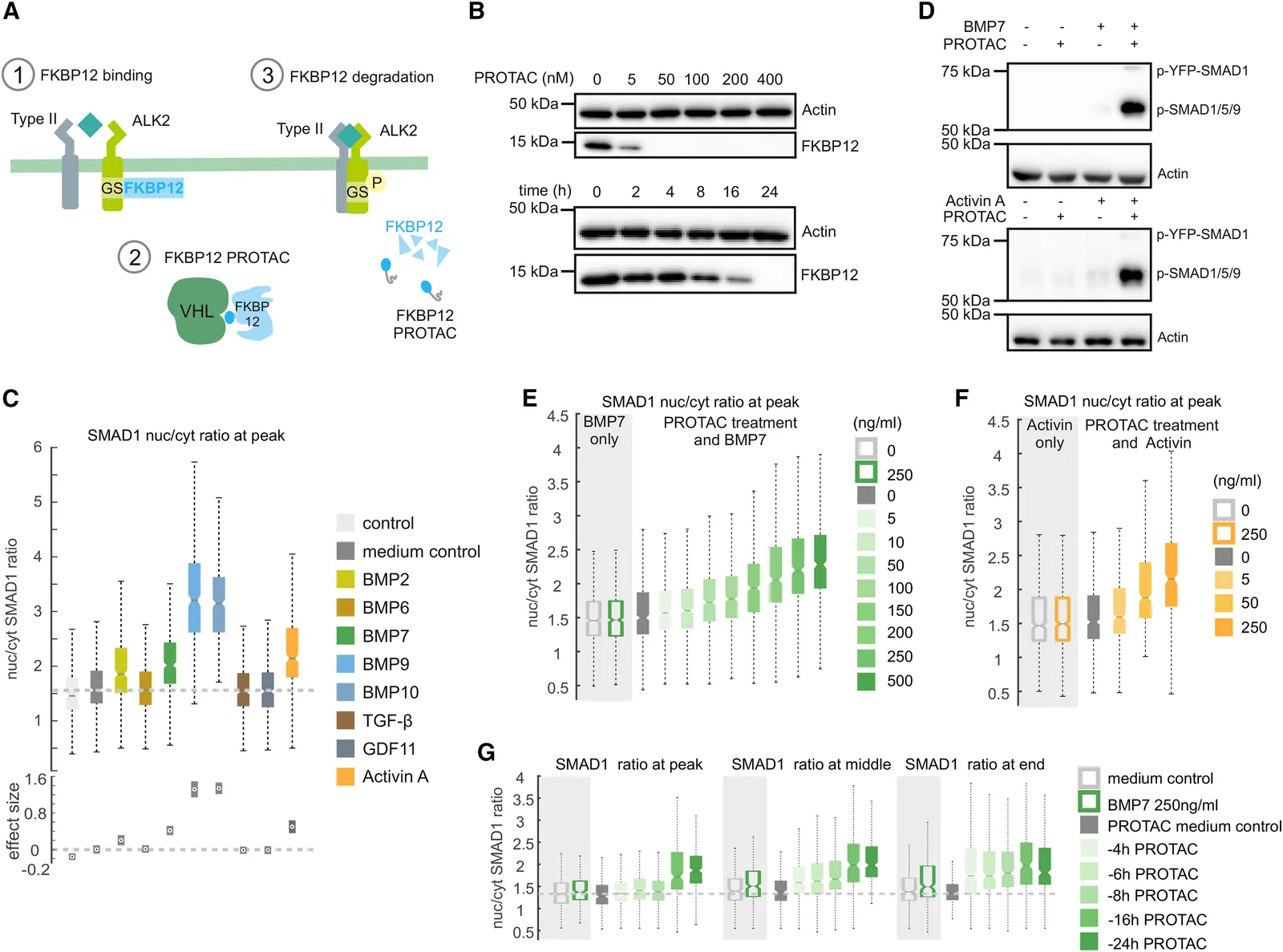
FKBP12 absence rescues SMAD response to ALK2-dependent ligands (A) Schematic of FKBP12 inhibition of ALK2 and its degradation by PROTAC, enabling ALK2 activation. (B) Western blot analysis of FKBP12 degradation in SMAD1 reporter cells. Top: dose-dependent PROTAC effect at 24 h. Bottom: time course of 200 nM PROTAC treatment. Actin served as loading control. Representative blots from two independent experiments are shown (B and D). (C) Peak nuc/cyt SMAD1 ratio following ligand stimulation with PROTAC pre-treatment. Upper part of the image: boxplots showing distribution of cellular responses. Lower part of the image: estimated changes in SMAD1 nuclear translocation compared to non-ligand stimulated cells. Error bars represent 90% confidence intervals determined by permutation testing. Dashed lines are provided as guides to the eye. (D) Western blot of SMAD1/5/9 phosphorylation 1.5 h after BMP7 (250 ng/ml) or Activin A (250 ng/ml) stimulation, with or without PROTAC pre-treatment. Actin served as loading control. (E and F) Dose-response analysis of peak nuc/cyt SMAD1 ratio for (E) BMP7 and (F) Activin A, with or without PROTAC pre-treatment. (G) Time-dependent effects of PROTAC addition on BMP7-induced SMAD1 nuclear translocation. Nuc/cyt SMAD1 ratios were analyzed at the peak of the response (around 1.5 h), at the middle of the observation period (12 h), and at the end (24 h). PROTAC (200 nM) was added 24 h before ligand stimulation in 1.5% FBS medium unless otherwise stated. Ligand concentrations as in Figure 3A. Imaging began 0.5 h before stimulation. Data in (C) and (E–G) are representative for three biological repeats.

Next, we depleted FKBP12 in SMAD reporter cells before stimulation with our selected ligands and monitored SMAD translocation by live-cell microscopy. Intriguingly, FKBP12 depletion led to increased SMAD1 activation in response to BMP7 and Activin A stimulation, which are mainly binding to ALK2 as type I receptor to activate the SMAD1/5/9 pathway (Figures 4C and S6). Consistently, we observed increased phosphorylation of SMAD1/5/9 under these conditions (Figure 4D). Interestingly, the SMAD response to BMP9 and BMP10 was almost unaffected despite reports that these ligands can utilize ALK2 as well (Figure S6).^33^^,^^34^ Next, we stimulated cells with varying doses of BMP7 and Activin A in the absence of FKBP12 and observed dose-dependent increases in SMAD1 translocation (Figures 4E and 4F). Remarkably, the dynamics of SMAD translocation were similar in all conditions that elicited a response. To determine the kinetics of FKBP12-mediated ALK2 inhibition, we pre-incubated cells with PROTAC for different periods of time before BMP7 stimulation (Figure 4G). It required several hours of PROTAC treatment to induce SMAD1 dynamics. However, cells pre-incubated for a shorter duration exhibited an elevated SMAD1 response at later time periods, suggesting a direct correlation between FKBP12 degradation and heightened SMAD1 signaling. In summary, these findings strongly support the hypothesis that FKBP12 blocks ALK2 in endothelial cells to prevent SMAD activation by certain ligands.

### FKBP12 regulates the balance of SMAD1/5/9 and SMAD2/3 activation by ligands of the BMP family

Our results support that FKBP12 inhibits SMAD1/5/9 activation by BMP7 and Activin A in untreated cells. However, we observed SMAD2/3 phosphorylation at least upon Activin A stimulation under these conditions (Figure 3A). Does loss of FKBP12 upon PROTAC treatment affect SMAD2/3 activation as well? Western blot analysis did not provide conclusive results when comparing conditions with or without FKBP12 (Figures S7A–S7F). We therefore established a clonal EA.hy926-based reporter cell line that expresses SMAD2 coupled to the red fluorescent protein mCherry in addition to YFP-SMAD1 (Figure 5A). We stimulated these dual reporter cells with varying doses of BMP9 and TGF-β. Interestingly, BMP9 exclusively stimulated SMAD1 translocation. SMAD2 remained cytoplasmic in individual cells despite the weak signals for phospho-SMAD2 previously observed in western blot analysis (Figure 5B, compare Figure 3A). Conversely, high doses of TGF-β resulted in noticeable SMAD2 translocation, reaching its peak at 5 ng/mL and showing a slight decrease in amplitude at higher doses (Figure 5C). There was no SMAD1 response detectable upon TGF-β stimulation. These observations indicate independent responses of the SMAD1/5/9 and SMAD2/3 branches to ALK1 and ALK5-activating ligands, respectively.

**Figure 5:**
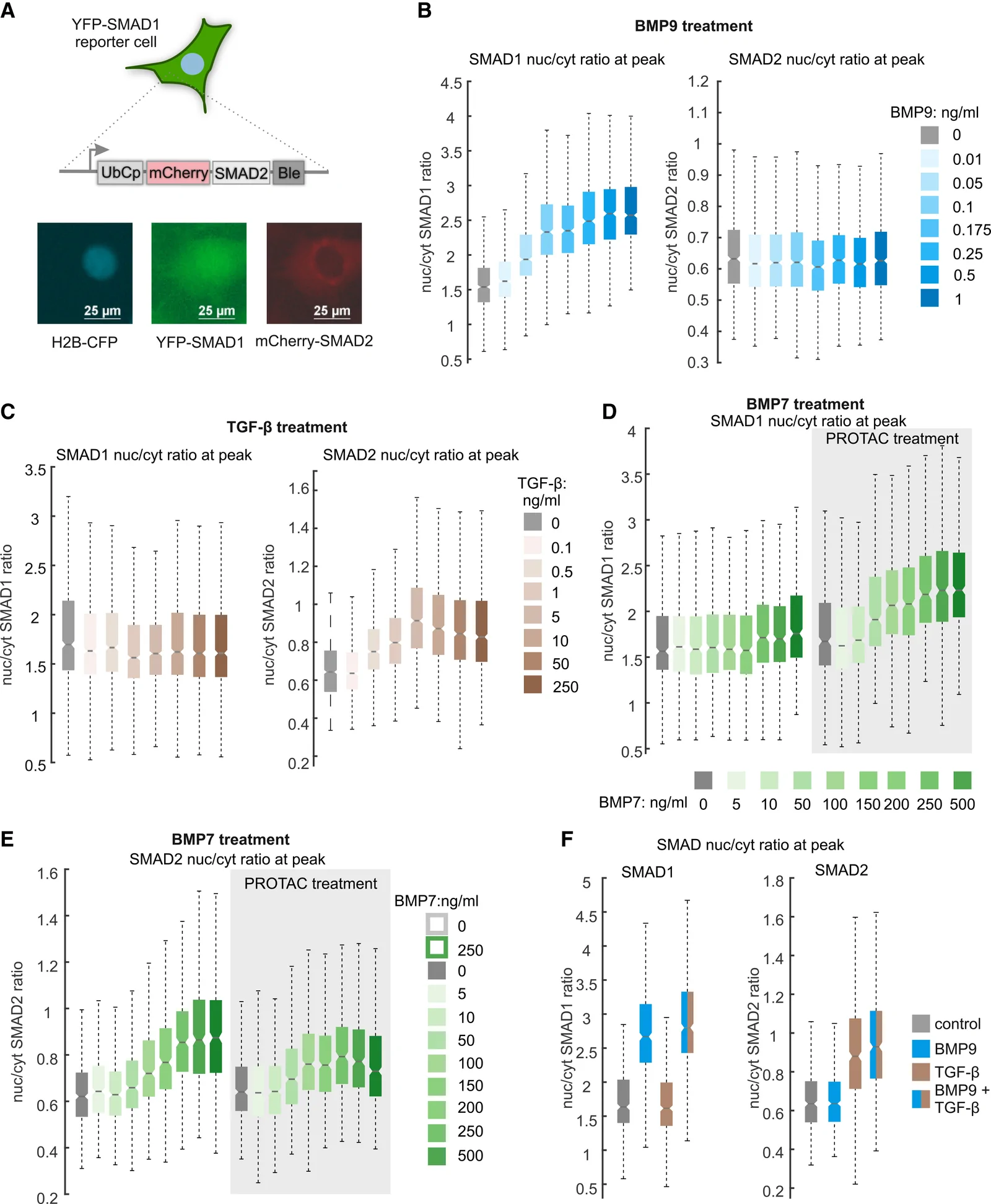
Dynamics of SMAD signaling in living cells (A) Schematic of the dual SMAD1/SMAD2 reporter system. Exemplary images are shown for all fusion proteins. Scale bars: 25 μm. (B and C) Dose-response analysis of peak nuc/cyt SMAD1 and SMAD2 ratios following stimulation with (B) BMP9 or (C) TGF-β. (D and E) Dose-response analysis of peak nuc/cyt SMAD1 (D) and SMAD2 (E) ratios following BMP7 stimulation, with or without PROTAC pre-treatment. (F) Comparison of peak nuc/cyt SMAD1 and SMAD2 ratios in response to BMP9 (1 ng/mL) and TGF-β (50 ng/mL) alone or in combination. Cells were cultured in 1.5% FBS medium for 20 h prior to stimulation. For (D) and (E), PROTAC (200 nM) was added 24 h before ligand stimulation. Time-lapse imaging was performed for 16 h, with ligands added 0.5 h after the start of imaging. Data in (B) and (C) are representative for four biological repeats and data in (D)–(F) for three biological repeats.

We next investigated the interplay between SMAD1 and SMAD2 dynamics upon treatment with ligands capable of engaging both signaling branches. Treating cells with BMP7 alone, we detected a weak SMAD1 and a robust SMAD2 response (Figures 5D and 5E). When we depleted FKBP12, though, we observed a shift of the response: SMAD1 translocation was augmented, particularly at higher concentrations, with a concomitant attenuation of the SMAD2 response (Figures 5D, 5E, S9A, and S9B). This could also be demonstrated by directly comparing maximal SMAD1 and SMAD2 responses at the single cell level (Figure S8A) and occurred upon Activin A treatment as well (Figure S8B). How can such a rebalancing of SMAD1 and SMAD2 signaling be explained mechanistically? Previously, it was suggested that signal shifts during co-treatment with ligands could be attributed to limited receptor numbers, feedback loops, or competition between subpathways.^35^^,^^36^

To test these hypotheses, we co-stimulated cells with BMP9 and TGF-β, activating both sub pathways via distinct type I and type II receptors. If there were feedback loops or competition between the two pathways, we would expect attenuated responses for SMAD1 and SMAD2 when comparing individual treatments with co-stimulation. However, co-stimulation did not noticeably alter SMAD responses (Figures 5F, S9C, and S9D).

We next analyzed the correlation between SMAD1 and SMAD2 dynamics. Immediately after stimulation, at the peak of the response, we observed similar correlation patterns upon stimulation with BMP7 and Activin A treatment in most cells (Figures S9E and S9F). However, at later times, a divergence of SMAD1 and SMAD2 responses could be observed in parts of the cell population, most pronounced upon BMP7 stimulation. These findings collectively support the hypothesis that the differential induction of SMAD1 and SMAD2 responses by BMP7 and Activin A upon PROTAC treatment stems from limited shared receptor availability.

### FKBP12 differentially affects homo- and heterodimeric receptor complexes

As our results failed to provide evidence for feedback inhibition or pathway competition, we focused on the hypothesis that the interplay of SMAD2/3 and SMAD1/5/9 is based on the activation of differential receptor complexes. To this end, we pharmacologically inhibited type I receptors and observed the resulting SMAD dynamics in the presence or absence of FKBP12. As expected, our analysis confirmed that BMP9 exclusively induces a SMAD1 response via ALK1/2 activation (Figure 6A). Inhibition of ALK1/2 also robustly suppressed SMAD1 responses upon BMP7 stimulation in the absence of FKBP12 (Figure 6B). Silencing receptor expression by siRNA substantiated the previously reported assignment of ALK1 mediating BMP9 and ALK2 mediating BMP7 signaling under these conditions (Figures S10A–S10C and S10E). Interestingly, ALK1/2 inhibition also prevented nuclear SMAD2 translocation independent of the FKBP12 status. In contrast, inhibition of ALK4/5 selectively impeded the SMAD2 response while leaving SMAD1 activation unaffected. Western blot analysis and siRNA-mediated silencing supported these observations (Figures S7B, S10D, and S10F). Finally, we investigated the receptor requirements for Activin A mediated SMAD responses. Similar to BMP7, SMAD1 activation upon Activin A treatment relied on ALK1/2, independent of the FKBP12 status (Figure 6C). However, ALK1/2 inhibition only partially blocked the SMAD2 response, both in the presence and absence of FKBP12, while it was completely prevented by ALK4/5 inhibition (compare Figure S7C for western blot analysis of SMAD2 phosphorylation).

**Figure 6:**
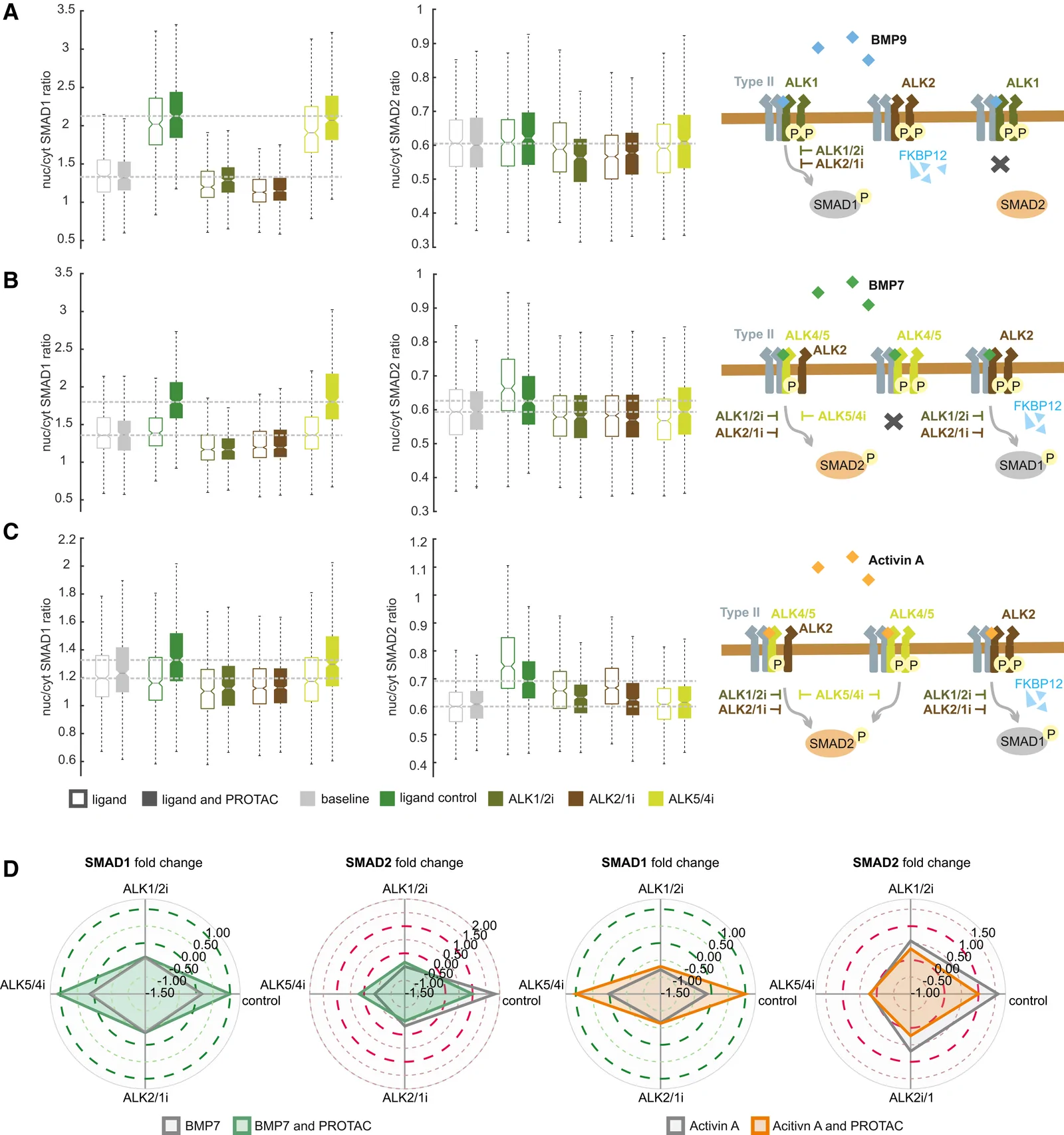
Differential SMAD signaling is based on ligand-specific receptor complexes (A–C) Peak nuc/cyt ratios of SMAD1 and SMAD2 upon stimulation with (A) BMP9 (1 ng/mL), (B) BMP7 (250 ng/mL), or (C) Activin A (250 ng/mL) over 16 h. Cells were pre-treated with PROTAC (200 nM) and ALK inhibitors ALK1/2i (K02288, 1 μM), ALK2/1i (LDN214117, 10 μM), and ALK5/4i (SB525334, 10 μM) as indicated. Dashed lines indicate baseline levels and activity upon stimulation in the absence of FKBP12, respectively, as guides for the eye. Schematics on the right illustrate a hypothetical model of SMAD activation by different receptor complexes. Figure S11 shows additional comparisons of basal nuc/cyt SMAD ratios to those after ALK inhibition alone and post-ligand stimulation. (D) Radar chart displaying fold changes in SMAD signaling under different inhibitor conditions. Baseline (0) represents median values from (A)–(C); ligand stimulation with PROTAC pre-treatment is normalized to 1. Cells were cultured in 1.5% FBS medium with or without PROTAC for 24 h. Inhibitors were added 0.5 h after experiment start, with ligands added 1 h later. Data are representative for three (A) and (B) or five (C) biological repeats.

Taken together, our results demonstrate that FKBP12 modulates the activity of specific receptor complexes and thereby determines the balance of SMAD1/5/9 and SMAD2/3 activation in a ligand-specific manner (Figure 6D). The data are compatible with a model where BMP7-induced SMAD2 activation is primarily driven by heterodimeric complexes of ALK2 and ALK4/5, while BMP7-induced SMAD1 activation in the absence of FKBP12 relies on ALK2 homodimers (Figure 6B, right side). Due to the observed SMAD2 translocation in the presence of ALK1/2 inhibitors, Activin A may also induce phosphorylation of SMAD2 via ALK4/5 homodimers (Figure 6C, right side).

### The balance of SMAD1/5/9 and SMAD2/3 activity affects target genes expression and phenotypic responses

Our previous research indicated that in mammary epithelial cells activation of the SMAD pathway leads to state-specific cellular responses that correlate with the extent of nuclear translocation rather than the activating ligands.^23^ We therefore aimed to investigate which cellular responses were elicited by SMAD2/3 and SMAD1/5/9 activation in endothelial cells. To this end, we examined expression profiles of selected target genes using RT-qPCR at an early time point (Figure 7A). Consistent with previous findings,^37^ Delta-like ligand 4 (DLL4) expression decreased significantly upon activation of the SMAD1/5/9 pathway upon BMP9 treatment and remained almost unchanged when only the SMAD2/3 pathway was stimulated by BMP7 in the presence of FKBP12 (Figure 7A). FKBP12 degradation led to a marked downregulation of DLL4 in response to BMP7 stimulation. In contrast, genes regulated by both pathways, such as ID2, exhibited more complex expression patterns. BMP7 alone increased ID2 expression, consistent with its role as a SMAD2/3 downstream target.^38^ FKBP12 removal further enhanced this effect, suggesting an additive regulation of ID2 by the FKBP12-ALK2-SMAD1/5/9 axis and the SMAD2/3 pathway (Figure 7B).

**Figure 7:**
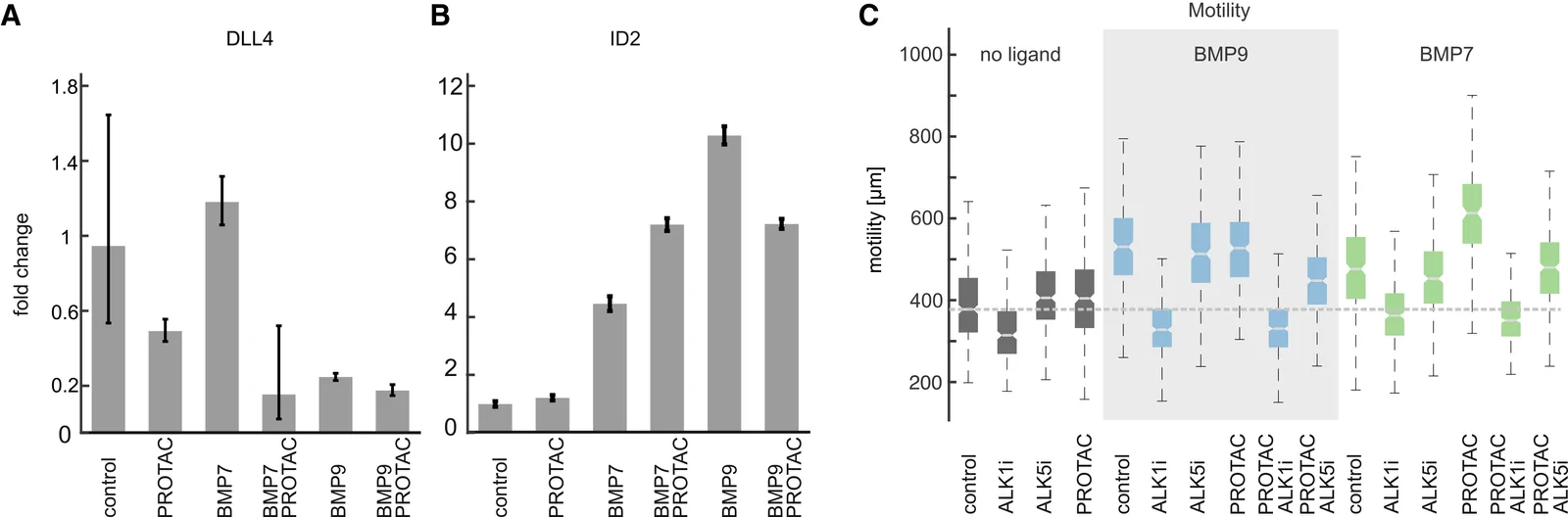
Differential SMAD activation affects downstream gene expression and cellular motility (A and B) RT-qPCR analysis of (A) DLL4 and (B) ID2 expression in SMAD1 reporter cells 1.5 h after stimulation with BMP9 (1 ng/mL) or BMP7 (250 ng/mL). Where indicated, FKBP12 PROTAC (200 nM) was added 24 h before stimulation. β-Actin served as internal control. Error bars represent standard deviation of technical triplicates. Data are representative for two biological repeats. (C) Quantification of motility in individual cells following treatment with BMP9 (1 ng/mL) or BMP7 (250 ng/mL). Cells were followed for 48 h by time-lapse microscopy and their cumulative displacement quantified. PROTAC (200 nM) was added 24 h and ALK1i (1 μM) or Alk5/4i (10 μM) were added 1 h before stimulation where indicated. See Figure S12 for replicate data. Cells were cultured in 1.5% FBS medium with or without PROTAC for 24 h. Data are representative for two biological repeats.

To determine how cell fates are affected over prolonged periods of time, we tracked individual cells for 48 h and quantified the cumulative distance traversed under different conditions. BMP9 treatment significantly enhanced cellular motility, an effect that was completely abolished by inhibition of ALK1 (Figures 7C and S12). Interestingly, BMP7 stimulation in the presence of FKBP12 also increased cell motility, albeit to a lesser extent than BMP9. BMP7 treatment in conjunction with FKBP12 degradation augmented single-cell motility, again suggesting an additive effect of both SMAD pathways. This enhanced motility was reduced by ALK4/5 inhibition (Figures 7C and S12).

In conclusion, we developed a new reporter system to reveal differential SMAD responses elicited by ligands of the BMP family in endothelial cells. We demonstrated that the adapter protein FKBP12 is a major factor for regulating receptor activity and thereby ligand sensitivity. Interestingly, FKBP12 also influences the balance of SMAD1/5/9 and SMAD2/3 signaling as well as the phenotypic response of endothelial cells to ligand stimulation.

## Discussion

SMAD signaling plays a crucial role in vascular development, homeostasis, and disease progression. The complexity of this network—encompassing diverse ligands, receptors, and signaling mediators—poses challenges in understanding how endothelial cells interpret and respond to extracellular signals. By analyzing SMAD dynamics at the single-cell level in a model of human vascular endothelial cells, we investigated redundancy and specialization of the signaling mediators SMAD1, SMAD5, and SMAD9. Genetic studies have indicated a certain degree of redundancy between these proteins.^39^ For example, SMAD1- and SMAD9-deficient embryos develop rather normally due to overlapping expression of SMAD5, although dose-dependent effects were observed in specific tissues.^40^^,^^41^ Our data corroborate differential responses at low ligand doses, suggesting that SMAD5 cannot compensate under such conditions. SMAD9, though part of general BMP signaling, has more specialized functions, for example in pulmonary vascular remodeling.^42^ We observed prolonged nuclear retention of SMAD9, which may be necessary for driving processes requiring sustained gene expression.^43^

Through profiling of SMAD activation by different ligands of the TGF-β superfamily in endothelial cells, we observed that only BMP9 and BMP10 induce strong SMAD1/5/9 activation in our reporter system. This is likely due to the predominant expression of their type I receptor, ALK1, in EA.hy926 cells.^29^ Other BMPs showed only limited activity, even at high concentrations, despite the presence of their receptors. We provide evidence that for the response to BMP7 and Activin A, this lack of activation is due to the presence of non-signaling complexes (NSCs).^44^ Mechanistically, we identified FKBP12 as a regulator of NSC formation in this context. However, the lack of SMAD activation by other ligands remains unexplained. BMP2, for example, induced only minimal SMAD1/5/9 responses despite FKBP12 removal. This contrasts with a previous study suggesting that BMP2, BMP4, and BMP10 signal through ALK2/BMPR2 complexes in the absence of FKBP12 in multiple myeloma cells.^45^ We also expected signaling activity by BMP6 due to its structural similarities and shared receptors with BMP7.^46^ It is interesting to note that BMP6 also exhibits distinct functional properties from BMP7, as it is for example resistant to Noggin inhibition.^47^ These differences highlight that structural homology does not always equate to functional equivalence and suggest that there may be additional factors influencing SMAD sensitivity to ligands.

To quantify the balance of SMAD1 and SMAD2 signaling over time, we developed a dual-reporter system. Co-treatment with TGF-β and BMP9 induced the same SMAD1 or SMAD2 responses as the individual ligands. This indicates parallel signaling without direct competition due to the utilization of distinct receptor complexes.^10^ Through comparative analysis, we sought to determine whether receptor complex composition may explain the specific signaling outcomes of Activin A and BMP7 stimulation in the presence or absence of FKBP12. Both BMP7 and Activin A activate SMAD1/5/9 through ALK2 when FKBP12 is degraded. However, we observed differences in SMAD2/3 activation. Our data support a model where BMP7-induced SMAD2 activation requires ALK4/ALK2 heterodimers, while Activin A uses both ALK4/ALK2 heterodimers and ALK4 homodimers. However, it is important to note that the pharmacological inhibitors used are limited in their specificity and that we only achieved partial knockdowns in siRNA experiments. In future studies, the proposed model of receptor complex composition needs to be substantiated by biochemical assays directly probing heterodimer formation. Nevertheless, our findings suggest that in addition to the previously described structural foundation of type I receptor specificity for R-SMADs,^48^^,^^49^ signaling specificity at the cellular level is also influenced by the configuration of FKBP12-receptor complexes. This could significantly impact a cell’s ability to generate ligand-specific SMAD responses.

Our study expands the current view of a competitive balance between SMAD1/5/9 and SMAD2/3 pathways in shaping endothelial cell phenotypes.^50^ We observed both exclusive regulation of target genes as well as additive effects by the two branches of the signaling pathway. Our cell motility assay further supports cooperative interaction. Collectively, our findings suggest that SMAD1/5/9 and SMAD2/3 can cooperate rather than compete to influence gene expression and functional outcomes in endothelial cells. FKBP12 emerges as a fine-tuning mechanism, modulating the balance and intensity of signaling through both pathways.

These results position FKBP12 as a potential therapeutic target for vascular disorders, allowing for selective modulation of SMAD pathways in BMP-related conditions. This aligns with clinical advancements using FK506 (tacrolimus) for HHT, where FK506 displaces FKBP12 from the TGF-β receptor complex, enhancing ALK1-SMAD1/5/9 signaling to reduce bleeding complications.^51^^,^^52^ In PAH, where BMPR2 mutations play a key role, FKBP12 targeting may similarly boost signaling through the remaining receptors.^53^^,^^54^ Emerging research supports the use of FKBP12-specific PROTACs as a promising strategy.^32^ Unlike FK506, which has immunosuppressive effects, these PROTACs offer increased selectivity by degrading FKBP12 without affecting the immune system or other FKBP family members. However, further studies are required to confirm the observed role of FKBP12 in primary endothelial cells and validate the therapeutic potential in *in vivo* models of vascular diseases. These models will also allow to determine potential off-target effects of FKBP12 PROTACs.

In conclusion, the results presented in this study deepen our understanding of SMAD signaling in endothelial cells by revealing how FKBP12 regulates the interaction between SMAD pathways and receptor complexes and open new avenues for therapeutic strategies based on quantitative modulation of SMAD signaling.

### Limitations of the study

EA.hy926 cells are a model system for human endothelia derived from male donors that is limited due to its hybrid nature combining endothelial properties with features of transformed cells. Further studies are needed to validate the proposed mechanisms in primary endothelial cells, cellular models derived from differentiated human stem cells or in *in vivo* model systems. Furthermore, we employed transgenic reporters based on exogenous expression of SMAD proteins fused to fluorescent proteins. While we carefully validated the functionality of these reporters through comparison with the parental cell line, expression of additional SMAD protein may potentially perturb the signaling system and thereby skew the observed results. In future studies, endogenous knock-in technology should be employed to overcome this limitation. Finally, we derived the proposed composition of receptor complexes indirectly from pharmacological and genetic perturbations. Further biochemical characterization of their molecular composition will provide deeper mechanistic insights into the stimulus-specific regulation of SMAD signaling in endothelial cells. Sex-related effects have not been assessed in this study.

## Resource availability

### Lead contact

Requests for further information and resources should be directed to and will be fulfilled by the lead contact, Alexander Loewer (loewer@bio.tu-darmstadt.de).

### Materials availability

Reporter cell lines and all stable reagents generated in this study are available from the lead contact, but we may require a completed material transfer agreement.

### Data and code availability

• Time series data of all tracked cells including data from biological repeats have been deposited at the institutional repository of Technical University Darmstadt and are publicly available as of the date of publication at https://doi.org/10.48328/tudatalib-2228.
• Raw image data reported in this paper will be shared by the lead contact upon request
• All original code has been deposited at the institutional repository of Technical University Darmstadt and is publicly available at https://doi.org/10.48328/tudatalib-2228 as of the date of publication.
• Any additional information required to reanalyze the data reported in this paper is available from the lead contact upon request.

## Acknowledgments

We thank Bettina Basso and Anna Kosin (Technical University Darmstadt) for technical assistance, Marc-André Fiedler for help characterizing SMAD9 reporter cells, Stefan Legewie and Laura Hartmann (Stuttgart University) as well as Petra Knaus and Jerome Latzlau (FU Berlin) for helpful discussions. This work was developed from the doctoral dissertation of Z.H. and funded by 10.13039/501100001659German Research Foundation—grant number 468927954 (to A.L.), the Hereditary hemorrhagic telangiectasia (HHT) foundation Germany (to A.L.), the BMFTR project ProxiTRAPS (03ZU1109CA to A.L. and F.H.), and a fellowship from the China Scholarship Council (to Z.H.).

## Author contributions

Z.H., S.L., A.B., and P.S. performed the experiments; F.H. provided critical resources; Z.H. and A.L. wrote the manuscript with contributions from all authors; A.L. conceived the study and supervised the research.

## Declaration of interests

The authors declare no competing interests.

## STAR★Methods

### Key resources table

**Table:** 

REAGENT or RESOURCE	SOURCE	IDENTIFIER
**Antibodies**		

Rabbit monoclonal anti-SMAD1	Invitrogen	Cat#MA5-41184; RRID: AB_2898938
Rabbit monoclonal anti-SMAD5	Invitrogen	Cat#MA5-32065; RRID: AB_2809359
Rabbit polyclonal anti-SMAD9	Invitrogen	Cat#720333; RRID: AB_2633263
Rabbit monoclonal anti-SMAD2	Cell Signaling Technology	Cat#5339; RRID: AB_10626777
Rabbit monoclonal anti-phospho-SMAD1/5/9	Cell Signaling Technology	Cat#13820; RRID: AB_2493181
Rabbit monoclonal anti-phospho-SMAD2	Cell Signaling Technology	Cat#3108;RRID: AB_490941
Rabbit polyclonal anti-phospho-SMAD3	Bioss	Cat#BS-3425R;RRID: AB_10857310
Rabbit monoclonal anti-FKPB12	HUABIO	Cat#ET1703-55; RRID: AB_3070411
Mouse monoclonal anti-β-Actin	Sigma-Aldrich	Cat#A5316; RRID: AB_476743

**Chemicals, peptides, and recombinant proteins**		

SYBR Green	Roche	Cat#04887352001
TGF-β	PeproTech	Cat#100-21C
GDF11	PeproTech	Cat#120–11
Activin A	PeproTech	Cat#120-14E
BMP2	PeproTech	Cat#120-02C
BMP6	PeproTech	Cat#120–06
BMP7	PeproTech	Cat#120-03P
BMP9	PeproTech	Cat#120–07
BMP10	PeproTech	Cat#120–40
K02288	Selleckchem	Cat#S7359
LDN-214117	Selleckchem	Cat#S7627
SB525334	Selleckchem	Cat#S1476
TEW-7197	Selleckchem	Cat#S7530
FKBP12 PROTACs (compound 5a1)	Geiger et al.^32^	N/A

**Critical commercial assays**		

High Pure RNA Isolation kit	Roche	Cat#11828665001

**Deposited data**		

Time series data	TUdatalib	https://doi.org/10.48328/tudatalib-2228

**Experimental models: Cell lines**		

EA.hy926	ATCC	Cat#CRL-2922; RRID: CVCL_3901
EA.hy926 mVenus-SMAD1	This paper	N/A
EA.hy926 mVenus-SMAD5	This paper	N/A
EA.hy926 mVenus-SMAD9	This paper	N/A
EA.hy926 mVenus-SMAD1 mCherry-SMAD2	This paper	N/A

**Oligonucleotides**		

Primer: β-actin forward: 5′-GGCACCCAGCACAATGAAGATCAA-3′ and reverse: 5′-TAGAAGCATTTGCGGTGGACGATG-3'	This paper	N/A
Primer: ID2 forward: 5′-TCCCAGGGTGTTCTCTTACTTGGACT-3′ and reverse: 5′-GGATCCTTCTGGTATTTCACGCTCCA-3'	This paper	N/A
Primer: EDN1 forward: 5′-ACTTCTGCCACCTGGACATC-3′ and reverse: 5′-GGCAAAAATTCCAGCACTTC-3'	This paper	N/A
Primer: DLL4 forward: 5′-TGCAACTGCCCTTATGGCTTTGTG-3′ and reverse: 5′-ACAAGTTGTTCATGGCTTCCCTGC-3	This paper	N/A
Primer: ALK1 forward 5′-ACAACATCCTAGGCTTCATCGC-3′ and reverse: 5′-GGTTTGCCCTGTGTACCG-3’	Hiepen et al.^29^	N/A
Primer: ALK2 forward: 5′-AAGCCTGGAGCATTGGTAA-3′ and reverse 5′- TCACTGGGGTACTCGGAGA-3’	Hiepen et al.^29^	N/A
Primer: ALK4 forward: 5′-TGCAACAGGATCGACTTGAG-3′ and reverse: 5′-ATGATGCCTACCAGCTCCAC-3’	Hiepen et al.^29^	N/A
Primer: ALK5 forward: 5′- ACTGTAAAGTCATCACCTGGC-3′ and reverse: 5′- GTGAATGACAGTGCGGTTGT-3’	Hiepen et al.^29^	N/A

**Recombinant DNA**		

pRRL-R4R3-NeoR-UbCp-mVenus-SMAD1	This paper	N/A
pRRL-R4R3-NeoR-UbCp-mVenus-SMAD5	This paper	N/A
pRRL-R4R3-NeoR-UbCp-mVenus-SMAD9	This paper	N/A
pRRL-R4R3-BlastR-UbCp-mCherry-SMAD2	This paper	N/A

**Software and algorithms**		

Custom image analysis algorithms	This paper	N/A
Fiji	Schindelin et al.^55^	https://imagej.net/software/fiji/
Floreada.io software	N/A	https://floreada.io/analysis
MATLAB	MathWorks	https://www.mathworks.com/
Quest Graph™ IC50 Calculator	AAT Bioquest, Inc.	https://www.aatbio.com/tools/ic50-calculator
Quest Graph™ EC50 Calculator	AAT Bioquest, Inc.	https://www.aatbio.com/tools/ec50-calculator
NIS-Elements	Nikon	https://www.microscope.healthcare.nikon.com/de_EU/products/software/nis-elements
StepOne Software	Applied Biosystems	https://www.thermofisher.com/de/de/home/technical-resources/software-downloads/StepOne-and-StepOnePlus-Real-Time-PCR-System.html
Analysis code	TUdatalib	https://doi.org/10.48328/tudatalib-2228

**Other**		

Nikon Ti-E inverted fluorescence microscope	Nikon	Ti-E inverted
24-well μ-Plate	ibidi	Cat#82426

### Experimental model and study participant details

#### Cell lines

EA.hy926 cells (originally from ATCC, Cat# CRL-2922) were a kind gift from Prof. Petra Knaus' lab (Freie Universität Berlin, Institute of Chemistry and Biochemistry - Biochemistry) and were maintained in M199 medium (Sigma Aldrich, Cat# M2154) supplemented with 20% fetal bovine serum (Bio&SELL, Cat# FBS.S 0615), 25 μg/mL heparin (Sigma Aldrich, Cat# H4784), 50 μg/mL endothelial cell growth supplement (ECGS) (Corning, Cat# 356006), 1% penicillin/streptomycin (Sigma, Cat# P0781), and 1% L-glutamine (Thermo Fisher Scientific, Cat# A2916801). For transgene selection and maintenance, the medium was supplemented with selective antibiotics as required, including 100 μg/mL G418 (Merck, CAS: 108321–42-2), 50 μg/mL Hygromycin B (Invitrogen, CAS: 31282–04-9), or 5 μg/mL Blasticidin S HCl (Thermo Fisher Scientific, Cat# A1113903). Cells were cultured at 37°C in a humidified atmosphere containing 5% CO2 and were passaged every 2–3 days. All cell lines were routinely tested for mycoplasma contamination and confirmed to be negative prior to experimentation.

### Method details

#### Plasmid construction

Plasmids were generated using Gateway cloning technology (Invitrogen). SMAD1, SMAD2, SMAD5, and SMAD9 were tagged with fluorescent proteins using a two-step Gateway cloning process. First, touchdown PCR was performed to amplify the cDNA sequences of SMAD1 (Cat# HG10715-M), SMAD2 (Cat# HG10016-M), SMAD5 (Cat# HG10017-M), and SMAD9 (Cat# HG12311-M) from Sino Biological, adding Gateway-compatible attB2R and attB3 overhangs. PCR products were cloned into the vector pDONR-P2R-P3 via BP reaction to create the entry vectors pENTR-P2R-P3-SMAD1, pENTR-P2R-P3-SMAD2, pENTR-P2R-P3-SMAD5, and pENTR-P2R-P3-SMAD9. The vectors pENTR-P4-P1R-UbCp (Ubiquitin C promoter), pENTR221-mVenus and pENTR221-mCherry were previously generated.^22^ Subsequently, LR reactions were performed using corresponding entry and destination vectors to generate the final expression vectors. For SMAD1, SMAD5, and SMAD9, the resulting constructs were pRRL-R4R3-Neo^R^-UbCp-mVenus-SMAD1, pRRL-R4R3-Neo^R^-UbCp-mVenus-SMAD5, and pRRL-R4R3-Neo^R^-UbCp-mVenus-SMAD9, respectively. For SMAD2, the construct pRRL-R4R3-Blast^R^-UbCp-mCherry-SMAD2 was generated. All cloning steps were verified by restriction enzyme digestion and DNA sequencing.

#### Lentiviral production and reporter cell line generation

Lentiviral particles were generated using standard procedures and EA.hy926 cells were transduced with lentiviral particles encoding reporter constructs along with viruses expressing histone 2B fused to cyan fluorescent protein (H2B-CFP) under the UbC promoter as a nuclear marker. The SMAD1- SMAD2 double reporter cell line was established by introducing SMAD2 fused to the red fluorescent protein mCherry into the existing SMAD1 reporter cell line. Following transduction, stable clonal cell lines were established and validated for their ability to monitor SMAD protein activity effectively.

#### Live-cell time-lapse microscopy

Reporter cells (0.48 × 10^5^) were plated in 24-well μ-Plate (ibidi, Cat# 82426) two days before experiments. The medium was replaced 20–24 h pre-imaging with phenol red- and riboflavin-free medium containing growth factors (Gibco, Cat# 2562725), 1.5% FBS (Bio&SELL, Cat# FBS.S 0615), and penicillin/streptomycin (Sigma, Cat# P0781). Imaging was performed on a Nikon Eclipse Ti-E inverted microscope with a 20× CFI Plan Fluor objective, Andor iXon Ultra 888 camera, and perfect focus system. Specific filter sets were used for Venus (500/20 nm excitation, 515 nm dichroic, 535/30 nm emission), CFP (436/20 nm excitation, 455 nm dichroic, 480/40 nm emission), and mCherry (562/40 nm excitation, 593 nm dichroic, 641/75 nm emission). Images were acquired every 10–20 min for the indicated durations using Nikon Elements software in an enclosure maintaining 37°C, 5% CO2, and humidity (OKO-Labs).

#### Ligand and inhibitor treatments

Recombinant proteins, including TGF-β (Cat# 100-21C), GDF11 (Cat# 120-11), Activin A (Cat# 120-14E), BMP2 (Cat# 120-02C), BMP6 (Cat# 120-06), BMP7 (Cat# 120-03P), BMP9 (Cat# 120-07), and BMP10 (Cat# 120-40), were sourced from Peprotech. TGF-β and GDF11 were stored at −80°C in a solution containing 4 mM HCl and 1 mg/mL BSA (BSA from Gibco, Cat# 2477575). Activin A was also stored at −80°C in 1 mg/mL BSA. The remaining ligands (BMP2, BMP6, BMP7, BMP9, and BMP10) were kept at −20°C, similarly prepared in 1 mg/mL BSA. Inhibitors K02288 (ALK1i, 1 μM, Selleckchem, Cat# S7359), LDN-214117 (ALK2/1i, 10 μM, Selleckchem, Cat# S7627), SB525334 (ALK5/4i, 10 μM, Selleckchem, Cat# S1476), TEW-7197 (ALK5/4i, 10 μM, Cat# S7530), and FKBP12 PROTACs (compound 5a1)^32^ were diluted in dimethyl sulfoxide (DMSO, Sigma, Cat# D2650) and stored at −20°C. Cells were imaged for 0.5 h before treatment. Ligands/inhibitors were prepared in 125 μL of 1.5% FBS medium and added to cells to achieve the desired concentration in a final volume of 1 mL. Control cells received medium only or medium with DMSO.

#### Western blot analysis

Cells were plated two days before experiments and harvested at indicated time points after stimulation. Proteins were isolated in the presence of protease and phosphatase inhibitors, and concentrations were measured by BCA assay (Thermo Fisher Scientific, Cat# 23225). Equal amounts of protein were separated by electrophoresis on 10–12.5% SDS-polyacrylamide gels and transferred to PVDF membranes (GE Healthcare, Cat# 10600023) by electroblotting. Membranes were blocked with 5% non-fat dried milk or 5% bovine serum albumin, incubated overnight with primary antibodies, washed, incubated with HRP-conjugated secondary antibodies, washed again, and detected using chemiluminescence (ECL Prime, GE Healthcare, Cat# RPN2232). The following primary antibodies were used: total SMAD1 (Invitrogen, Cat# MA5-41184), SMAD5 (Invitrogen, Cat# MA5-32065), SMAD9 (Invitrogen, Cat# 720333), SMAD2 (D43B4, Cell Signaling Technology, Cat# 5339), Phospho-SMAD1(Ser463/465)/5(Ser463/465)/9(Ser463/465) (Cell Signaling Technology, Cat# 13820), Phospho-Smad2 (Ser465/467) (Cell Signaling Technology, Cat# 3108), Phospho-Smad3 (Ser423, Ser425) (Bioss, Cat# BS-3425R), FKBP12 (HUABIO, Cat# ET1703-55), and β-actin (Sigma-Aldrich, Cat# A5316). Secondary antibodies were HRP conjugated anti-mouse IgG (Thermo Fisher Scientific, Cat# 31430) and HRP conjugated anti-rabbit IgG (Thermo Fisher Scientific, Cat# 31460). Band intensities were measured using Fiji.^55^

#### Real-time quantitative PCR (qPCR)

Cells were plated two days before experiments and harvested at indicated time points after stimulation. Total RNA was extracted using the High Pure RNA Isolation kit (Roche, Cat# 11828665001). RNA concentration was determined using a NanoDrop 2000 spectrophotometer (Thermo Fisher Scientific, Cat# ND-2000). Complementary DNA was synthesized from 1 μg of RNA using ProtoScript II reverse transcriptase (New England Biolabs, Cat# E6560L) with oligo-dT primers. Quantitative PCR was performed in triplicates using SYBR Green reagent (Roche, Cat# 04887352001) on a StepOnePlus PCR machine (Applied Biosystems, Cat# 10096224). The following primer sequences were used: β-actin forward: 5′-GGCACCCAGCACAATGAAGATCAA-3′ and reverse: 5′-TAGAAGCATTTGCGGTGGACGATG-3'; ID2 forward: 5′-TCCCAGGGTGTTCTCTTACTTGGACT-3′ and reverse: 5′-GGATCCTTCTGGTATTTCACGCTCCA-3'; EDN1 forward: 5′-ACTTCTGCCACCTGGACATC-3′ and reverse: 5′-GGCAAAAATTCCAGCACTTC-3'; DLL4 forward: 5′-TGCAACTGCCCTTATGGCTTTGTG-3′ and reverse: 5′-ACAAGTTGTTCATGGCTTCCCTGC-3'; ALK1 forward 5′-ACAACATCCTAGGCTTCATCGC-3′ and reverse: 5′-GGTTTGCCCTGTGTACCG-3’; ALK2 forward: 5′-AAGCCTGGAGCATTGGTAA-3′ and reverse 5′- TCACTGGGGTACTCGGAGA-3’; ALK4 forward: 5′-TGCAACAGGATCGACTTGAG-3′ and reverse: 5′-ATGATGCCTACCAGCTCCAC-3’; ALK5 forward: 5′- ACTGTAAAGTCATCACCTGGC-3′ and reverse: 5′- GTGAATGACAGTGCGGTTGT-3’. Relative expression was calculated using the ΔΔCt method with β-actin as endogenous control. Data was collected and analyzed using StepOne Software v2.3.5.7.

#### Flow cytometry

Cells were trypsinized and fixed in 70% ethanol at 20 °C. For DNA content analysis, cells were washed in PBS, incubated with 25 g/mL propidium iodide (PI), 0.1% Triton, and 0.2 g/mL RNase, and analyzed on a Biorad S3 Cell Sorter. Only cell singlets were analyzed, on the basis of the pulse width vs. height ratio. To obtain the percentages of G1, S, G2/M cells, corresponding populations were manually gated. Analysis was performed with the online tool “https://floreada.io”.

### Quantification and statistical analysis

#### Image analysis and cell tracking

Cell tracking and analysis were performed using custom MATLAB (2021a) scripts, based on code from the Alon laboratory^56^ and the CellProfiler project.^57^ In brief, raw images underwent flat-field correction, background subtraction and were segmented based on the nuclear H2B-CFP marker using thresholding and seeded watershed algorithms.^22^ A greedy match algorithm tracked cells across time points, excluding those leaving the field of view or dying. Nuclear fluorescence intensity was measured directly, while cytoplasmic fluorescence was quantified using a 4-pixel wide annulus around each nucleus. Data processing included artifact removal and smoothing of nuclear area and integrated fluorescence intensity. The nuclear-to-cytoplasmic (nuc/cyt) ratio was calculated over time, with values interpolated during mitosis. Median fluorescence values ensured robustness against fluctuations. This approach allowed for comprehensive analysis of single-cell trajectories. To assess effect sizes and their significance between conditions, permutation testing with 1000 permutations was performed, calculating changes in population means from 20% subsamples of imaged cells within each condition and establishing 90% confidence intervals.

#### Quantification of cell motility

Cell motility was quantified by calculating Euclidean distances between consecutive positions of individual cells using their x and y coordinates in pixels. To mitigate tracking errors, trajectories where the distance covered between two time points exceeded three times the average distance of all cells were excluded. Pixel-based distances were converted to actual distances using the objective and camera resolution. Finally, the distance moved by each cell during the course of the experiment was calculated by summing up the displacement between time points.

#### Data analysis and visualization

To analyze the time required for nuclear export of SMAD proteins upon receptor inhibition (Figure 2H), the dataset was normalized to initial values of the relevant conditions and median responses across populations were calculated. The largest change between consecutive time points was identified and its occurrence time recorded. The time point where the signal returned to approximately 1.1 times the baseline value was identified. The recovery time was then calculated as the time difference between addition of the ligand and this recovery point.

For EC_50_ and IC_50_ analysis, median values were calculated for each condition; peak values and their corresponding time points were identified and extracted. To assess variability, first (Q1) and third (Q3) quartiles of peak data were calculated for each condition, with error margins computed based on these quartiles. The concentration and peak data were used to fit a corresponding function and calculate EC_50_/IC_50_ values using Quest Graph IC50/EC50 Calculator (AAT Bioquest).

To create radar charts (Figure 6), peak values of the SMAD2 nuc/cyt ratio over specified time intervals were identified for each condition. These values were organized into a matrix, with missing values set to NaN. Median values for each condition were calculated and normalized by subtracting the median of the base condition without FKBP12 PROTAC, setting it to zero. The ligand stimulation with PROTAC condition was set as the reference with a median value of one. The normalized data were divided into two groups and combined into a single matrix for plotting. The radar chart was generated using the ‘radarChart’ function in MATLAB.

For correlating SMAD1 and SMAD2 nuclear translocation dynamics (Figure S8), peak responses of individual reporter cells were determined by using the ‘findpeaks’ function in MATLAB to identify maxima within the first 5 h post-ligand addition. If multiple peaks existed, the maximum value was selected; if no peak was detected, the value was set to 1^–10^. Results were visualized using various plotting techniques.

To analyze the correlation between SMAD1 and SMAD2 response at the single-cell level (Figure S9F), data were initially normalized using the bsxfun function in MATLAB, and the normalized SMAD1 and SMAD2 data for the condition of interest were extracted. A correlation matrix was computed to determine Pearson correlation coefficients between SMAD1 and SMAD2 for each cell, with NaN values replaced by 1^–10^ for computational stability. To validate the correlation results, a bootstrap analysis with 100 iterations was performed. For each cell and iteration, a temporary matrix was created by randomly permuting the SMAD2 data order, and correlation coefficients were calculated between the SMAD1 data and the permuted SMAD2 data.

To evaluate FKBP12’s impact on SMAD1 and SMAD2 responses across BMP7 concentrations (Figure S9A), maximum values at different time points were organized into a matrix for each concentration. Median values were calculated for each condition and time point, then normalized by dividing by the initial median value from the baseline (no FKBP12 treatment). Peak ratio values with FKBP12 were standardized to 1 for each BMP7 concentration, expressing changes as percentages. Percentage differences between FKBP12-treated and untreated conditions were computed and visualized using scatterplots. For analyzing TGF-β and BMP9 co-treatment experiment (Figure S9B), SMAD1 and SMAD2 responses were analyzed using different reference points: BMP9-only treatment for SMAD1 and TGF-β-only treatments at various doses for SMAD2, both set to a reference value of 1. Otherwise, the analysis was performed as described above.

#### Statistical analysis

Statistical analyses were performed using MATLAB (version 2021a, MathWorks) unless otherwise stated. The number of biological replicates, and experimental repeats are indicated in the corresponding figure legends. Data are presented as median values unless otherwise indicated. Confidence intervals, permutation testing, bootstrap analysis, and correlation analyses were performed as described above.

## Supplemental information

Document S1. Figures S1–S12

Data S1. Raw western blot images

Data S2. Raw western blot images of additional repeats

Table S1. Number of cells analyzed for indicated figure panels

## References

[1] Kulikauskas M.R., X S., Bautch V.L. (2022). The versatility and paradox of BMP signaling in endothelial cell behaviors and blood vessel function. Cell. Mol. Life Sci..

[2] Shi Y., Massagué J. (2003). Mechanisms of TGF-β Signaling from Cell Membrane to the Nucleus. Cell.

[3] David C.J., Massagué J. (2018). Contextual determinants of TGFβ action in development, immunity and cancer. Nat. Rev. Mol. Cell. Bio.

[4] Goumans M.-J., Ten Dijke P. (2018). TGF-β Signaling in Control of Cardiovascular Function. Cold Spring Harb. Perspect. Biol..

[5] Goumans M.J., Zwijsen A., ten Dijke P., Bailly S. (2018). Bone morphogenetic proteins in vascular homeostasis and disease. Cold Spring Harb. Perspect. Biol..

[6] Benn A., Hiepen C., Osterland M., Schütte C., Zwijsen A., Knaus P. (2017). Role of bone morphogenetic proteins in sprouting angiogenesis: Differential BMP receptor-dependent signaling pathways balance stalk vs. tip cell competence. FASEB J..

[7] Gaengel K., Genové G., Armulik A., Betsholtz C. (2009). Endothelial-mural cell signaling in vascular development and angiogenesis. Arterioscler Thromb Vasc Biol..

[8] Kaneda H., Arao T., Matsumoto K., De Velasco M.A., Tamura D., Aomatsu K., Kudo K., Sakai K., Nagai T., Fujita Y. (2011). Activin A inhibits vascular endothelial cell growth and suppresses tumour angiogenesis in gastric cancer. Br. J. Cancer.

[9] Maeshima K., Maeshima A., Hayashi Y., Kishi S., Kojima I. (2004). Crucial Role of Activin A in Tubulogenesis of Endothelial Cells Induced by Vascular Endothelial Growth Factor. Endocrinology.

[10] Heldin C.-H., Moustakas A. (2016). Signaling Receptors for TGF-β Family Members. Csh Perspect Biol.

[11] Mueller T.D., Nickel J. (2012). Promiscuity and specificity in BMP receptor activation. FEBS Lett..

[12] David L., Mallet C., Mazerbourg S., Feige J.J., Bailly S. (2007). Identification of BMP9 and BMP10 as functional activators of the orphan activin receptor-like kinase 1 (ALK1) in endothelial cells. Blood.

[13] Macίas-Silva M., Hoodless P.A., Tang S.J., Buchwald M., Wrana J.L. (1998). Specific Activation of Smad1 Signaling Pathways by the BMP7 Type I Receptor, ALK2. J. Biol. Chem..

[14] Olsen O.E., Wader K.F., Hella H., Mylin A.K., Turesson I., Nesthus I., Waage A., Sundan A., Holien T. (2015). Activin A inhibits BMP-signaling by binding ACVR2A and ACVR2B. Cell Commun. Signal..

[15] Chen Y.G., Liu F., Massagué J. (1997). Mechanism of TGFbeta receptor inhibition by FKBP12. EMBO J..

[16] Wang T., Li B.Y., Danielson P.D., Shah P.C., Rockwell S., Lechleider R.J., Martin J., Manganaro T., Donahoe P.K. (1996). The immunophilin FKBP12 functions as a common inhibitor of the TGF beta family type I receptors. Cell.

[17] Miyazawa K., Miyazono K. (2017). Regulation of TGF-β family signaling by inhibitory smads. Cold Spring Harb. Perspect. Biol..

[18] Abdalla S.A., Letarte M. (2006). Hereditary haemorrhagic telangiectasia: current views on genetics and mechanisms of disease. J. Med. Genet..

[19] Thenappan T., Ormiston M.L., Ryan J.J., Archer S.L. (2018). Pulmonary arterial hypertension: pathogenesis and clinical management. BMJ.

[20] Cunha S.I., Magnusson P.U., Dejana E., Lampugnani M.G. (2017). Deregulated TGF-β/BMP Signaling in Vascular Malformations. Circ. Res..

[21] Roman B.L., Hinck A.P. (2017). ALK1 signaling in development and disease: new paradigms. Cell. Mol. Life Sci..

[22] Strasen J., Sarma U., Jentsch M., Bohn S., Sheng C., Horbelt D., Knaus P., Legewie S., Loewer A. (2018). Cell-specific responses to the cytokine TGF β are determined by variability in protein levels. Mol. Syst. Biol..

[23] Bohn S., Hexemer L., Huang Z., Strohmaier L., Lenhardt S., Legewie S., Loewer A. (2023). State- And stimulus-specific dynamics of SMAD signaling determine fate decisions in individual cells. Proc Natl. Acad. Sci. USA.

[24] Edgell C.J., McDonald C.C., Graham J.B. (1983). Permanent cell line expressing human factor VIII-related antigen established by hybridization. Proc. Natl. Acad. Sci. USA.

[25] Warmflash A., Zhang Q., Sorre B., Vonica A., Siggia E.D., Brivanlou A.H. (2012). Dynamics of TGF- signaling reveal adaptive and pulsatile behaviors reflected in the nuclear localization of transcription factor Smad4. Proc. Natl. Acad. Sci. USA.

[26] Clarke D.C., Brown M.L., Erickson R.A., Shi Y., Liu X. (2009). Transforming Growth Factor β Depletion Is the Primary Determinant of Smad Signaling Kinetics. Mol. Cell Biol..

[27] Massagué J., Kelly B. (1986). Internalization of transforming growth factor-β and its receptor in BALB/c 3T3 fibroblasts. J. Cell. Physiol..

[28] Koli K.M., Arteaga C.L. (1997). Processing of the transforming growth factor β type I and II receptors. Biosynthesis and ligand-induced regulation. J. Biol. Chem..

[29] Hiepen C., Jatzlau J., Hildebrandt S., Kampfrath B., Goktas M., Murgai A., Cuellar Camacho J.L., Haag R., Ruppert C., Sengle G. (2019). BMPR2 acts as a gatekeeper to protect endothelial cells from increased TGFβ responses and altered cell mechanics. PLoS Biol..

[30] Upton P.D., Davies R.J., Trembath R.C., Morrell N.W. (2009). Bone Morphogenetic Protein (BMP) and Activin Type II Receptors Balance BMP9 Signals Mediated by Activin Receptor-like Kinase-1 in Human Pulmonary Artery Endothelial Cells. J. Biol. Chem..

[31] Peiffer B.J., Qi L., Ahmadi A.R., Wang Y., Guo Z., Peng H., Sun Z., Liu J.O. (2019). Activation of BMP Signaling by FKBP12 Ligands Synergizes with Inhibition of CXCR4 to Accelerate Wound Healing. Cell Chem. Biol..

[32] Geiger T.M., Walz M., Meyners C., Kuehn A., Dreizler J.K., Sugiarto W.O., Maciel E.V.S., Zheng M., Lermyte F., Hausch F. (2024). Discovery of a Potent Proteolysis Targeting Chimera Enables Targeting the Scaffolding Functions of FK506-Binding Protein 51 (FKBP51). Angew. Chem. Int. Ed..

[33] Herrera B., van Dinther M., ten Dijke P., Inman G.J. (2009). Autocrine bone morphogenetic protein-9 signals through activin receptor-like kinase-2/Smad1/Smad4 to promote ovarian cancer cell proliferation. Cancer Res..

[34] Mazerbourg S., Sangkuhl K., Luo C.W., Sudo S., Klein C., Hsueh A.J.W. (2005). Identification of receptors and signaling pathways for orphan bone morphogenetic protein/growth differentiation factor ligands based on genomic analyses. J. Biol. Chem..

[35] Yan X., Xiong X., Chen Y.-G. (2018). Feedback regulation of TGF-β signaling. Acta Biochim. Biophys. Sin..

[36] Martinez-Hackert E., Sundan A., Holien T. (2021). Receptor binding competition: A paradigm for regulating TGF-β family action. Cytokine Growth Factor Rev..

[37] Rostama B., Turner J.E., Seavey G.T., Norton C.R., Gridley T., Vary C.P.H., Liaw L. (2015). DLL4/Notch1 and BMP9 Interdependent Signaling Induces Human Endothelial Cell Quiescence via P27KIP1 and Thrombospondin-1. Arterioscler. Thromb. Vasc. Biol..

[38] Veerasamy M., Phanish M., Dockrell M.E.C. (2013). Smad Mediated Regulation of Inhibitor of DNA Binding 2 and Its Role in Phenotypic Maintenance of Human Renal Proximal Tubule Epithelial Cells. PLoS One.

[39] Attisano L., Lee-Hoeflich S.T. (2001). The Smads. Genome Biol..

[40] Tremblay K.D., Dunn N.R., Robertson E.J. (2001). Mouse embryos lacking Smad1 signals display defects in extra-embryonic tissues and germ cell formation. Development.

[41] Arnold S.J., Maretto S., Islam A., Bikoff E.K., Robertson E.J. (2006). Dose-dependent Smad1, Smad5 and Smad8 signaling in the early mouse embryo. Dev. Biol..

[42] Machado R.D., Southgate L., Eichstaedt C.A., Aldred M.A., Austin E.D., Best D.H., Chung W.K., Benjamin N., Elliott C.G., Eyries M. (2015). Pulmonary Arterial Hypertension: A Current Perspective on Established and Emerging Molecular Genetic Defects. Hum. Mutat..

[43] Huang Z., Wang D., Ihida-Stansbury K., Jones P.L., Martin J.F. (2009). Defective pulmonary vascular remodeling in Smad8 mutant mice. Hum. Mol. Genet..

[44] Klumpe H.E., Langley M.A., Linton J.M., Su C.J., Antebi Y.E., Elowitz M.B. (2022). The context-dependent, combinatorial logic of BMP signaling. Cell Syst..

[45] Quist-Løkken I., Andersson-Rusch C., Kastnes M.H., Kolos J.M., Jatzlau J., Hella H., Olsen O.E., Sundan A., Knaus P., Hausch F., Holien T. (2023). FKBP12 is a major regulator of ALK2 activity in multiple myeloma cells. Cell Commun. Signal..

[46] Gipson G.R., Goebel E.J., Hart K.N., Kappes E.C., Kattamuri C., McCoy J.C., Thompson T.B. (2020). Structural perspective of BMP ligands and signaling. Bone.

[47] Song K., Krause C., Shi S., Patterson M., Suto R., Grgurevic L., Vukicevic S., van Dinther M., Falb D., ten Dijke P., Alaoui-Ismaili M.H. (2010). Identification of a key residue mediating bone morphogenetic protein (BMP)-6 resistance to noggin inhibition allows for engineered BMPs with superior agonist activity. J. Biol. Chem..

[48] Chaikuad A., Bullock A.N. (2016). Structural basis of intracellular TGF-β signaling: Receptors and smads. Cold Spring Harb. Perspect. Biol..

[49] Hata A., Chen Y.G. (2016). TGF-β signaling from receptors to smads. Cold Spring Harb. Perspect. Biol..

[50] Hiepen C., Mendez P.-L., Knaus P. (2020). It Takes Two to Tango: Endothelial TGFβ/BMP Signaling Crosstalk with Mechanobiology. Cells.

[51] Albiñana V., Sanz-Rodríguez F., Recio-Poveda L., Bernabéu C., Botella L.M. (2011). Immunosuppressor FK506 increases endoglin and activin receptor-like kinase 1 expression and modulates transforming growth factor-β1 signaling in endothelial cells. Mol. Pharmacol..

[52] Hessels J., Kroon S., Boerman S., Nelissen R.C., Grutters J.C., Snijder R.J., Lebrin F., Post M.C., Mummery C.L., Mager J.J. (2022). Efficacy and Safety of Tacrolimus as Treatment for Bleeding Caused by Hereditary Hemorrhagic Telangiectasia: An Open-Label, Pilot Study. J. Clin. Med..

[53] Shah A.J., Beckmann T., Vorla M., Kalra D.K. (2023). New Drugs and Therapies in Pulmonary Arterial Hypertension. Int. J. Mol. Sci..

[54] Orriols M., Gomez-Puerto M.C., Ten Dijke P. (2017). BMP type II receptor as a therapeutic target in pulmonary arterial hypertension. Cell. Mol. Life Sci..

[55] Schindelin J., Arganda-Carreras I., Frise E., Kaynig V., Longair M., Pietzsch T., Preibisch S., Rueden C., Saalfeld S., Schmid B. (2012). Fiji: an open-source platform for biological-image analysis. Nat. Methods.

[56] Cohen A.A., Geva-Zatorsky N., Eden E., Frenkel-Morgenstern M., Issaeva I., Sigal A., Milo R., Cohen-Saidon C., Liron Y., Kam Z. (2008). Dynamic Proteomics of Individual Cancer Cells in Response to a Drug. Science.

[57] Carpenter A.E., Jones T.R., Lamprecht M.R., Clarke C., Kang I.H., Friman O., Guertin D.A., Chang J.H., Lindquist R.A., Moffat J. (2006). CellProfiler: image analysis software for identifying and quantifying cell phenotypes. Genome Biol..

