# Supplementary material for: FKBP12 determines ligand-specific SMAD signaling in individual living endothelial cells
Source: iScience. 2026 Sep 22;29(10):117728. doi: 10.1016/j.isci.2026.117728 (PMC13659684; doi:10.1016/j.isci.2026.117728)
Supplement: Supplementary file 1 [file mmc1.pdf]

iScience, Volume *29*

Supplemental information

FKBP12 determines ligand-specific SMAD signaling in individual living endothelial cells

Zixin Huang, Sonja Lenhardt, Petra Snyder, Ahmed Bulldan, Felix Hausch, and Alexander Loewer

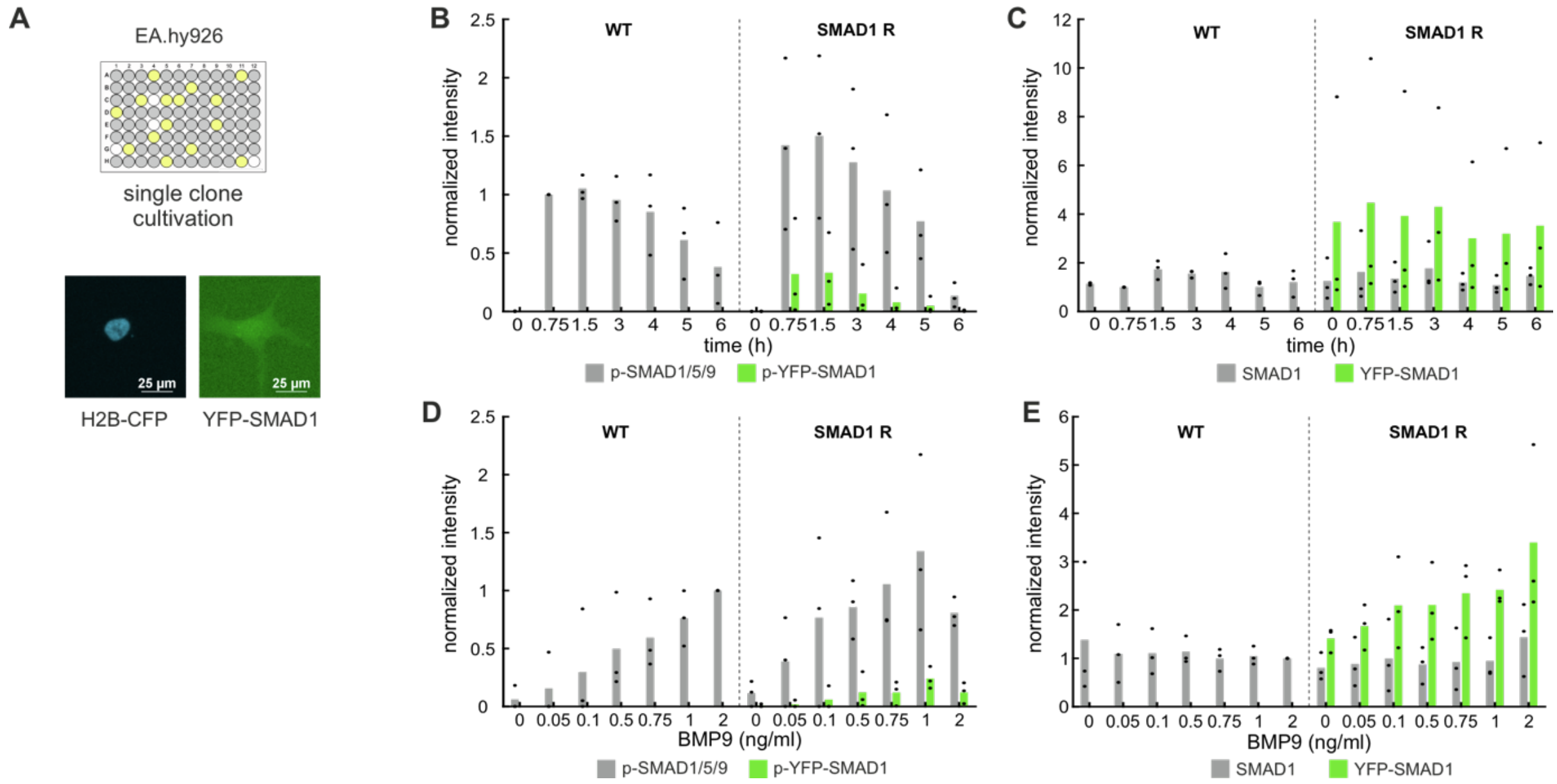

Figure S1. Characterization of the SMAD1 reporter cell line. (A) Clonal cell lines of the fluorescent reporters were established by limited dilution. Exemplary microscopy images are shown for the expressed fusion proteins. Scale bar: 25 µm. **(B-E)** Quantification of Western blot analysis of SMAD1 activation in SMAD1 reporter cells and parental EA.hy926 wildtype cells (Figure 1B). Phosphorylated SMAD1/5/9 and total SMAD1 were examined at indicated time points after stimulation with 1 ng/ml BMP9 (B-C), as well as 1.5h after stimulation with varying doses of the ligand (D-E). Band intensities were measured using Fiji and then normalized in relation to the loading control ß-Actin and the WT 0.75 h (B-C) and WT 2 ng/ml (D-E) condition, respectively. **Note**: For all experiments, cells were cultured in 1.5% FBS medium for 20 hours prior to BMP9 stimulation.

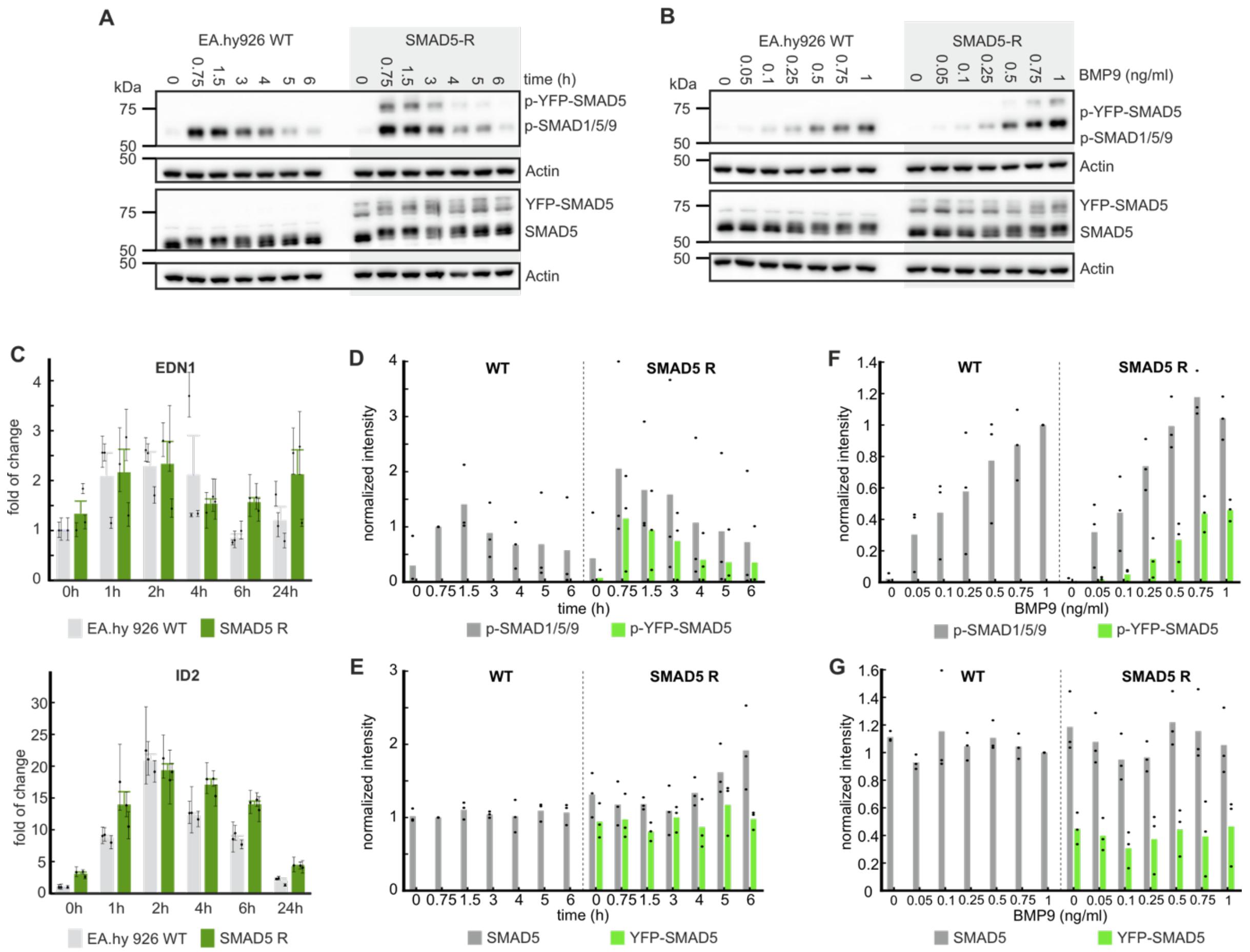

Figure S2. Characterization of SMAD5 reporter cell line. (A) SMAD5 reporter cells were stimulated with 1 ng/ml BMP9, harvested at the indicated time points and compared with EA.hy926 WT cells. p-SMAD1/5/9 is shown on the top, total SMAD5 on the bottom. **(B)** SMAD5 reporter cells were stimulated with the indicated BMP9 concentrations, harvested after 1.5 hours and compared with EA.hy926 WT cells. p-SMAD1/5/9 antibody results are shown on the top, total SMAD5 on the bottom. Representative blots from three independent experiments are shown (A, B). **(C)** RT-qPCR analysis comparing SMAD target gene expression kinetics in EA.hy926 WT and EA.hy926 SMAD5 reporter cells following 1 ng/ml BMP9 stimulation. β-Actin served as internal control. **(D-G)** Quantification of Western blot analysis of SMAD5 activation in SMAD5 reporter cells and parental EA.hy926 wildtype cells. Band intensities were measured using Fiji and then normalized in relation to the loading control ß-Actin and the WT 0.75 h (D-E) and WT 2 ng/ml (F-G) condition, respectively. **Note**: For all experiments, cells were cultured in 1.5% FBS medium for 20 hours prior to BMP9 stimulation.

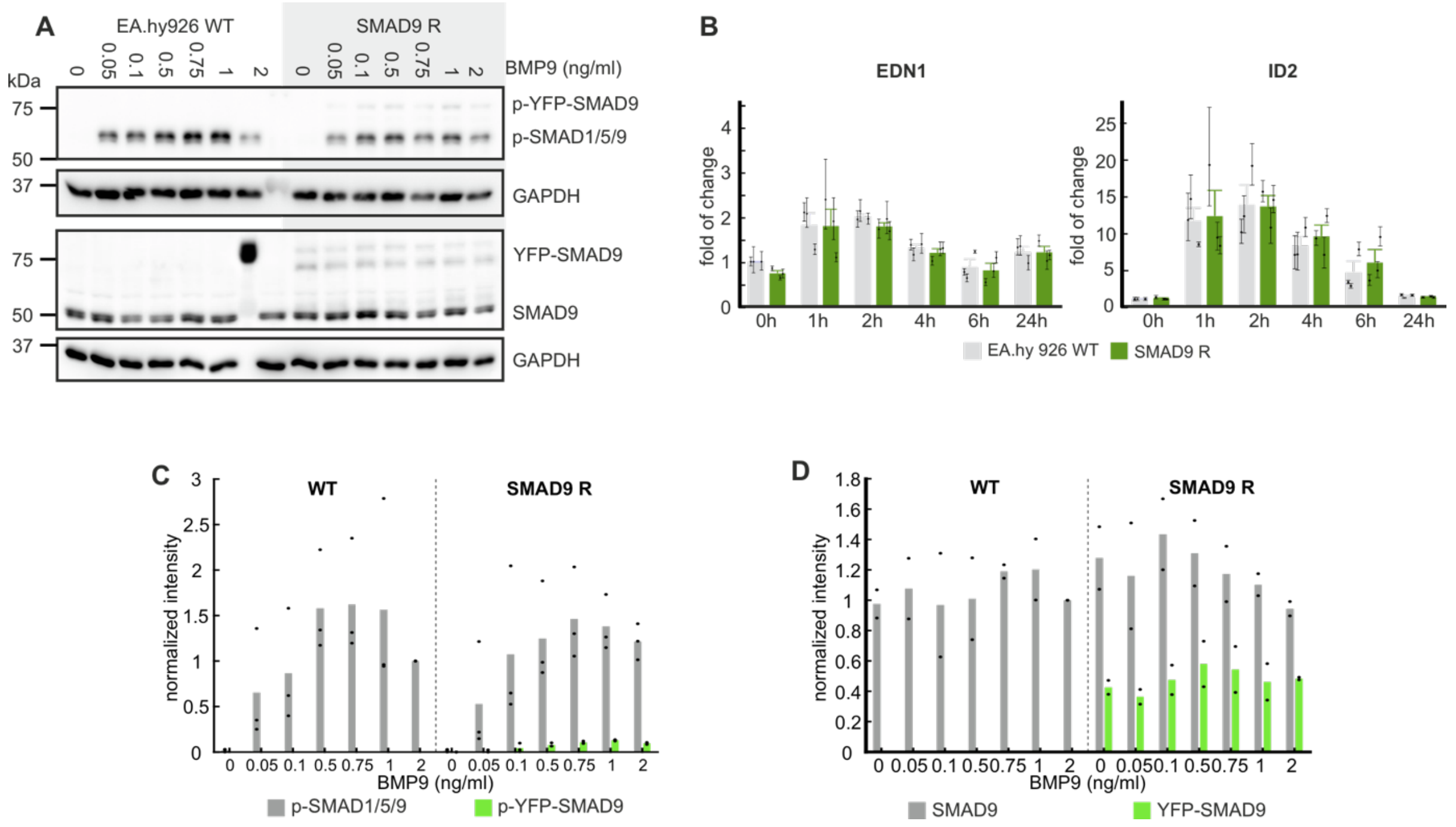

Figure S3. Characterization of SMAD9 reporter cell line. (A) SMAD9 reporter cells were stimulated with the indicated BMP9 concentrations, harvested after 1.5 hours and compared with EA.hy926 WT cells. p-SMAD1/5/9 antibody results are shown on the top, total SMAD9 results on the bottom. Representative blots from three independent experiments are shown. **(B)** RT-qPCR analysis comparing SMAD target gene expression kinetics in EA.hy926 WT and EA.hy926 SMAD9 reporter cells following 1 ng/ml BMP9 stimulation. Colored bars represent mean ± standard deviation (SD) of biological triplicates, individual data points ± standard deviation (SD) of technical triplicates are shown in black. **(C-D)** Quantification of Western blot analysis of SMAD9 activation in SMAD9 reporter cells and parental EA.hy926 wildtype cells. Band intensities were measured using Fiji and then normalized in relation to the loading control ß-Actin and WT 2 ng/ml condition. **Note**: For all experiments, cells were cultured in 1.5% FBS medium for 20 hours prior to BMP9 stimulation.

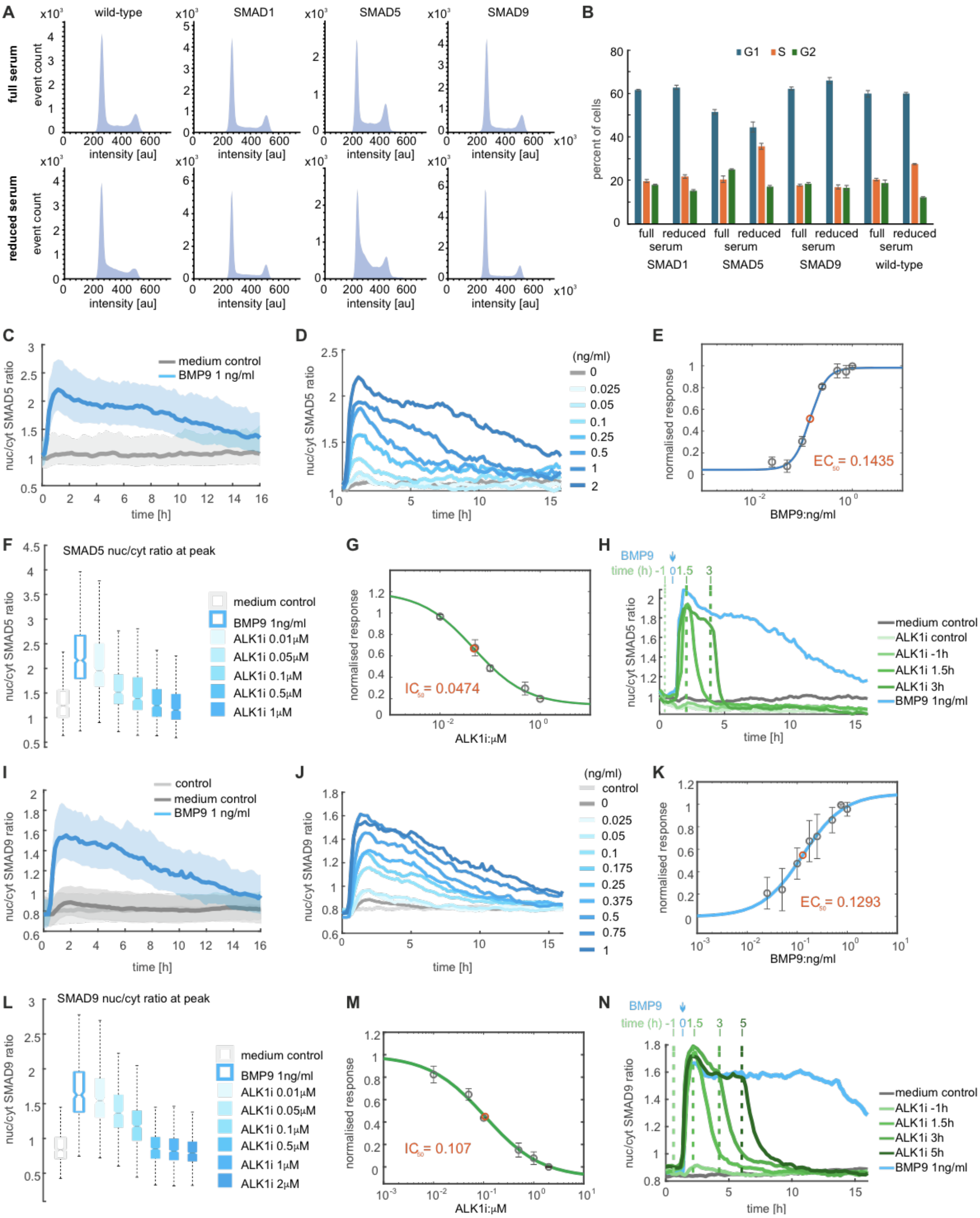

Figure S4. Dynamics of SMAD5 and SMAD9 signaling in living cells. (A-B) Cell cycle profiles of wt and SMAD reporter cells. (A) DNA content was measured by flow cytometry for cells grown in medium with 10% FBS (upper row) or 1.5% FBS (lower row) for 20h. The percentage of cells in each cell cycle phase was quantified from flow cytometry. Mean and SD from biological triplicates are shown. **(C-H)** Analysis of SMAD5 dynamics: **(C)** Median nuc/cyt SMAD5 ratio upon 1 ng/ml BMP9 stimulation (blue), unstimulated (light gray), or medium control (dark gray). **(D)** Median nuc/cyt SMAD5 ratio following varying BMP9 concentrations. **(E)** EC_{50} calculation (orange) based on peak median nuc/cyt SMAD5 ratio for each BMP9 concentration. **(F)** Peak median nuc/cyt SMAD5 ratio with the indicated ALK1i (K02288) concentrations 1 hour prior to BMP9 stimulation. **(G)** IC_{50} calculation (orange) for

ALK1i based on peak median nuc/cyt SMAD5 ratio. **(H)** ALK1i efficacy at different timepoints relative to BMP9 induction. **(I-N)** Analysis of SMAD9 dynamics: **(I)** Median nuc/cyt SMAD9 ratio upon 1 ng/ml BMP9 stimulation (blue), unstimulated (light gray), or medium control (dark gray). **(J)** Median nuc/cyt SMAD9 ratio following varying BMP9 concentrations. **(K)** EC_{50} calculation (orange) based on peak median nuc/cyt SMAD9 ratio for each BMP9 concentration. **(L)** Peak median nuc/cyt SMAD9 ratio with various ALK1i concentrations 1 hour prior to BMP9 stimulation. **(M)** IC_{50} calculation (orange) for ALK1i based on peak median nuc/cyt SMAD9 ratio. **(N)** ALK1i efficacy at different timepoints relative to BMP9 induction. **Note**: For all experiments, cells were pre-cultured in 1.5% FBS medium for 20 hours prior to stimulation. Time-lapse imaging was performed every 10 minutes for 16 hours, starting 0.5 hours before stimulation. Solid lines represent median values; shaded areas indicate 25th to 75th percentiles. Data in E, G, K, and M represent mean ± SD from two independent experiments, data in C, D, F, H-J, L and N are representative of two biological repeats. DMSO served as control for inhibitor experiments.

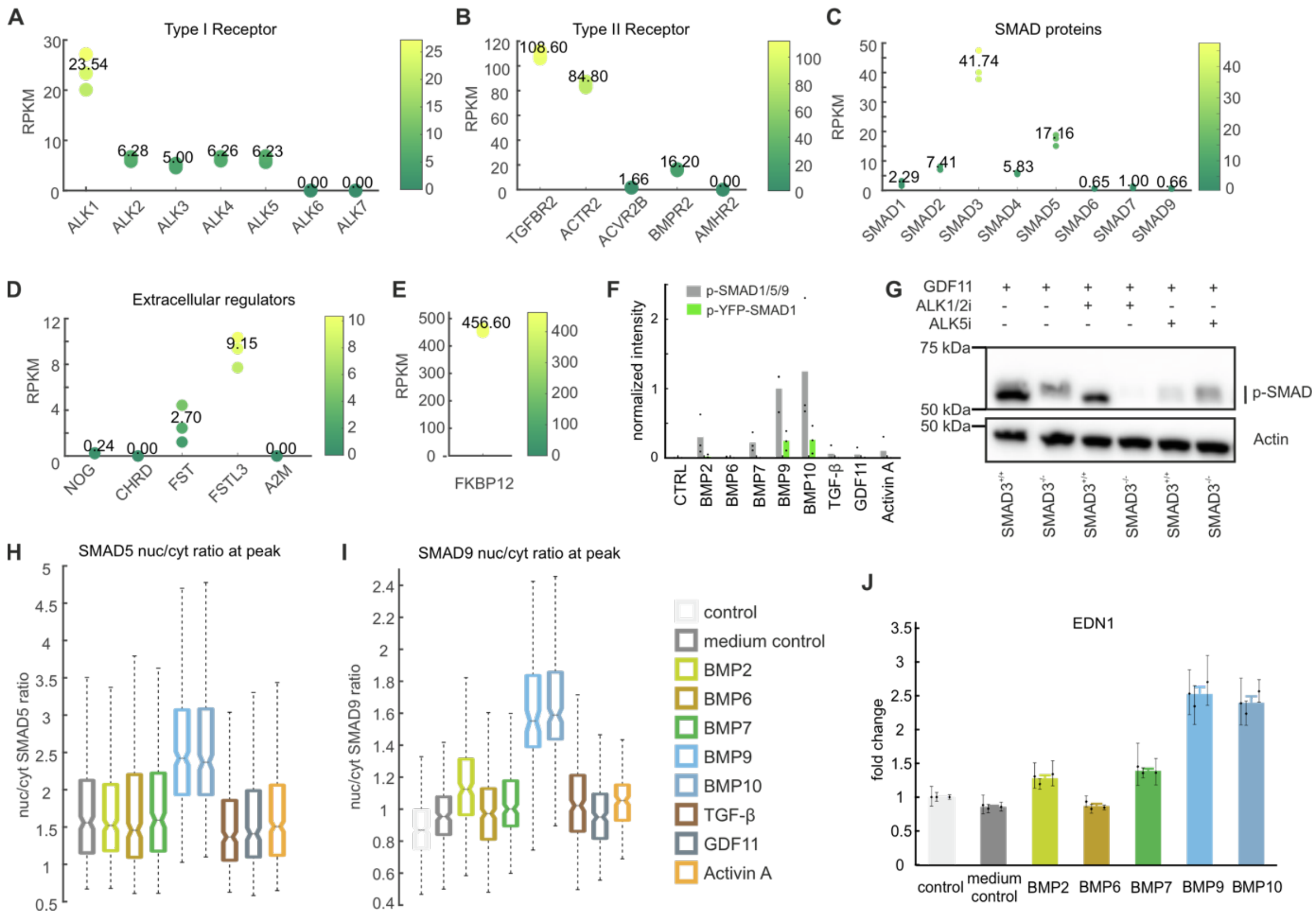

Figure S5. Expression levels of key BMP pathway components and ligand-induced nuclear translocation of SMAD5 and SMAD9 (A-E) RNA-seq data showing expression levels of type I and II receptors (A-B), SMADs (C), inhibitory proteins in the BMP pathway (D), and FKBP12 (E) in EA.hy926 cells. RNA-seq data from Hiepen et al., 2019. **(F)** Quantification of Western blot analysis of SMAD1/5/9 phosphorylation in SMAD1 reporter cells (Figure 3A). Phosphorylated SMAD1/5/9 was examined 1.5h after stimulation with varying ligands. Band intensities were measured using Fiji and then normalized in relation to the loading control ß-Actin and the mean intensity of the BMP9 condition **(G)** Western blot analysis of phosphorylated SMAD3 in wildtype and SMAD3 KO MCF10A breast epithelial cells treated with GDF11 (250 ng/ml) and indicated inhibitors (ALK1/2i: K02288, 1 µM; ALK5i: SB525334, 10 µM) Samples were harvested 1.5h post-stimulation. Actin served as loading control. Data is representative of two biological repeats. **(H-I)** Peak nuc/cyt ratios of SMAD5 (H) and SMAD9 (I) following stimulation with the indicated ligands over 16 hours. Ligand concentrations for SMAD5 were as follows: BMP2 (1400 ng/ml), BMP6 (1310 ng/ml), BMP7, GDF11, Activin A (250 ng/ml), BMP9 (1 ng/ml), BMP10 (2 ng/ml), and TGF-β (50 ng/ml). For SMAD9, ligand concentrations included BMP2 (7 ng/ml), BMP6 (1310 ng/ml), BMP7, GDF11, Activin A (250 ng/ml), BMP9 (1 ng/ml), BMP10 (2 ng/ml), and TGF-β (50 ng/ml). Cells were cultured in 1.5% FBS medium for 20 hours prior to stimulation. Time-lapse imaging was performed every 10 minutes for 16 hours, starting 0.5 hours before stimulation. Data is representative of two biological repeats. **(J)** RT-qPCR analysis of EDN1 expression in SMAD1 reporter cells treated with the indicated BMP ligands. Fold changes were calculated in relation to unstimulated cells and ß-Actin as a reference. Colored bars represent mean ± standard deviation (SD) of biological triplicates, individual data points ± standard deviation (SD) of technical triplicates are shown in black. Ligand concentrations: BMP2 (250 ng/ml), BMP6 (250 ng/ml), BMP7 (250 ng/ml), BMP9 (1 ng/ml), BMP10 (1 ng/ml).

A

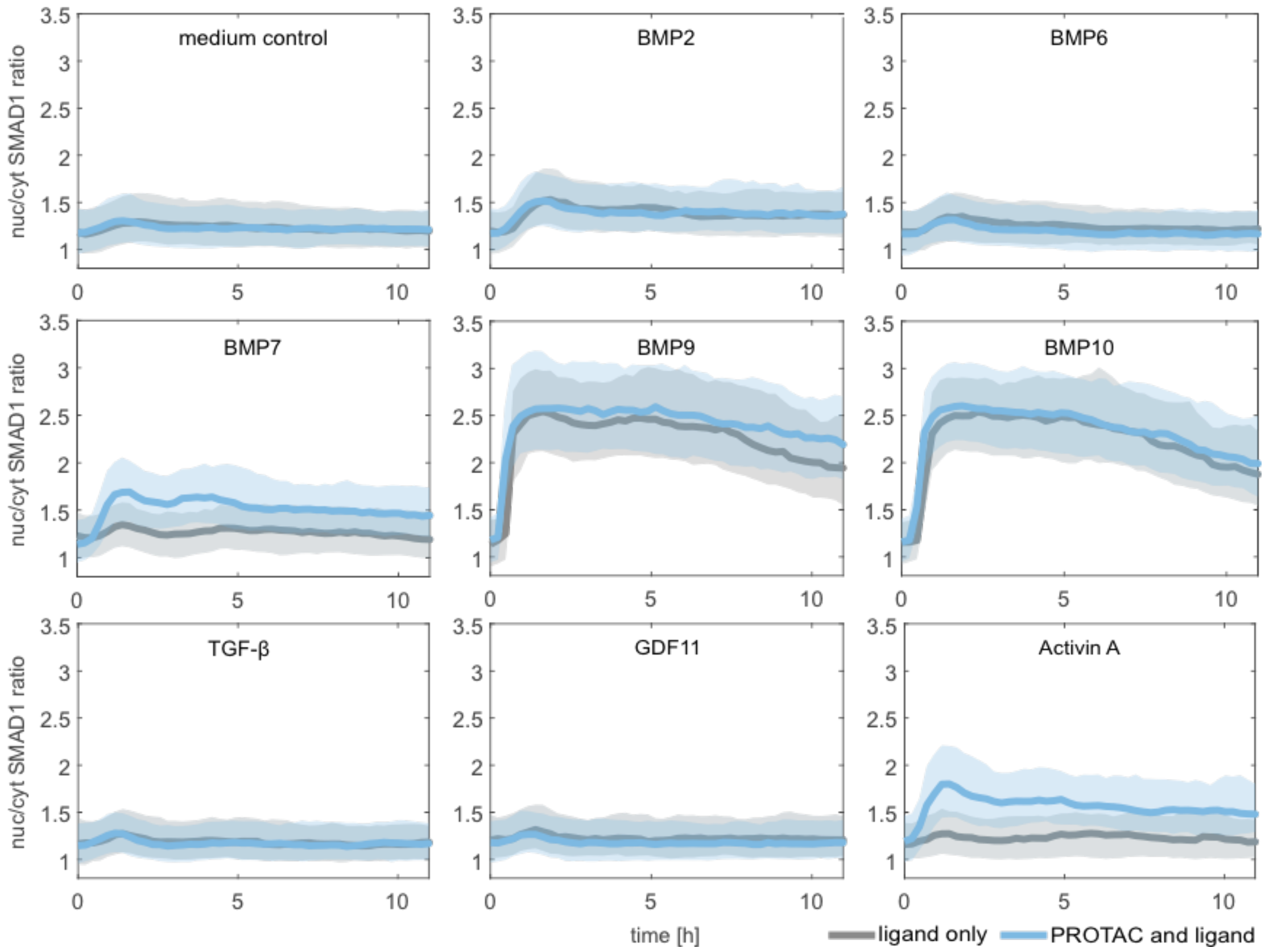

Figure S6. SMAD1 nuclear translocation dynamics upon FKBP12 degradation. (A) Median nuc/cyt SMAD1 ratio following stimulation with indicated TGF-β superfamily ligands in SMAD1 reporter cells, with (blue) or without (grey) FKBP12 PROTAC pre-treatment. Cells were cultured in 1.5% FBS medium for 24 hours with or without FKBP12 PROTAC prior to stimulation. Ligand concentrations were identical to those used in Figure 3A. Solid lines represent median values; shaded areas indicate 25th to 75th percentiles of the population. Data is representative of three biological repeats.

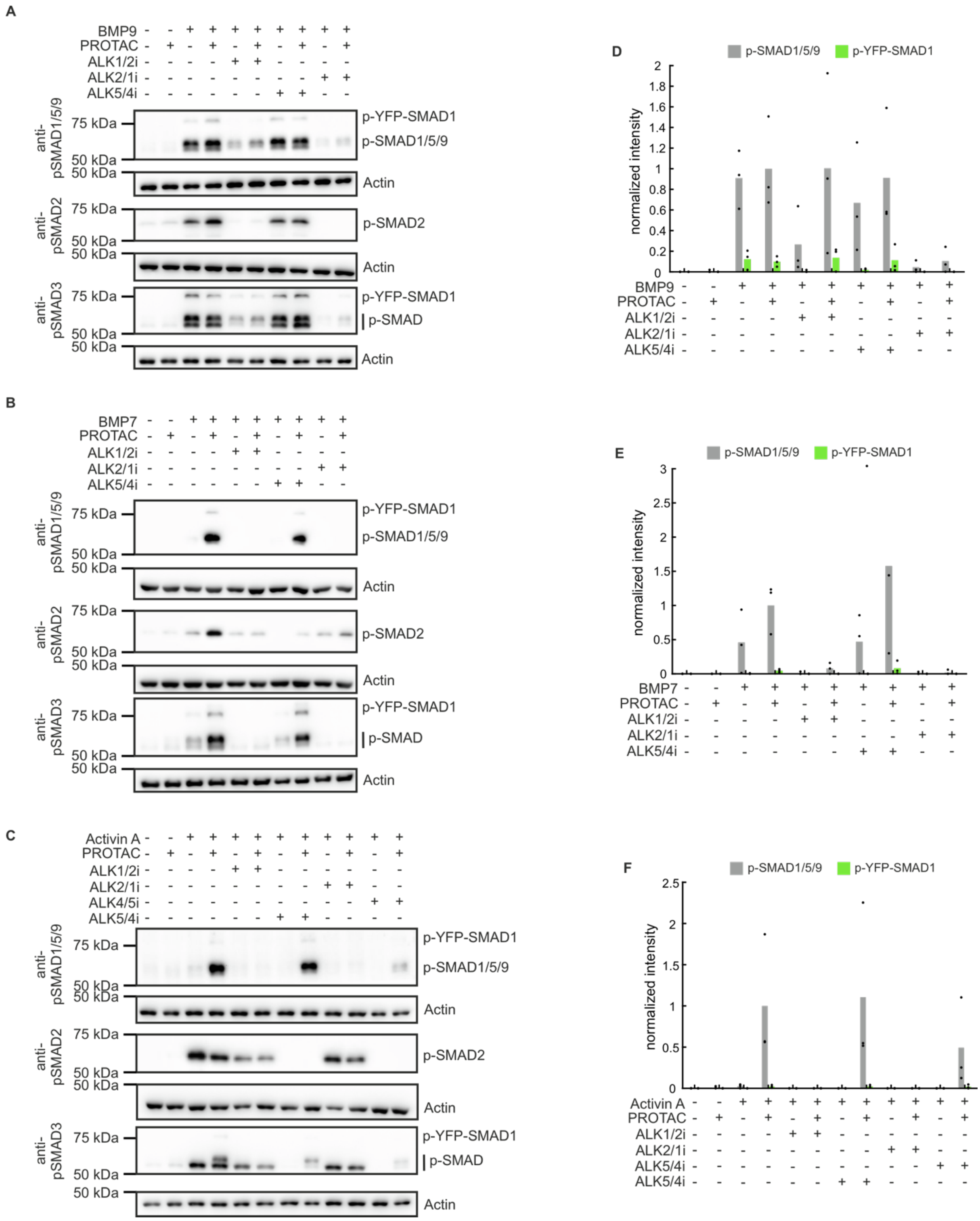

Figure S7. Differential SMAD activation upon ligand stimulation. (A-C) Western blot analysis of phosphorylated SMAD1/5/9, SMAD2, and SMAD3 in SMAD1 reporter cells treated with BMP9 (1 ng/ml) (A), BMP7 (250 ng/ml) (B), or Activin A (250 ng/ml) (C). Effects of PROTAC pre-treatment and indicated ALK inhibitors are shown. PROTAC was added 24h before ligand stimulation in 1.5% FBS medium. Inhibitors (ALK1/2i: K02288, 1 μM; ALK2/1i: LDN214117, 10 μM; ALK4/5i: TEW7179, 10 μM; ALK5/4i: SB525334, 10 μM) were added 1h before ligands. Samples were harvested 1.5h post-stimulation. Upper plot: p-SMAD1/5/9; middle plot: p-SMAD2; lower plot: p-SMAD3. Actin served as loading control. Representative blots from three independent experiments are

shown. **(D-F)** Quantification of Western blot analysis of phosphorylated SMAD1/5/9 in SMAD1 reporter cells. Band intensities were measured using Fiji and then normalized in relation to the loading control ß-Actin and the mean intensity of the PROTAC-BMP9 condition.

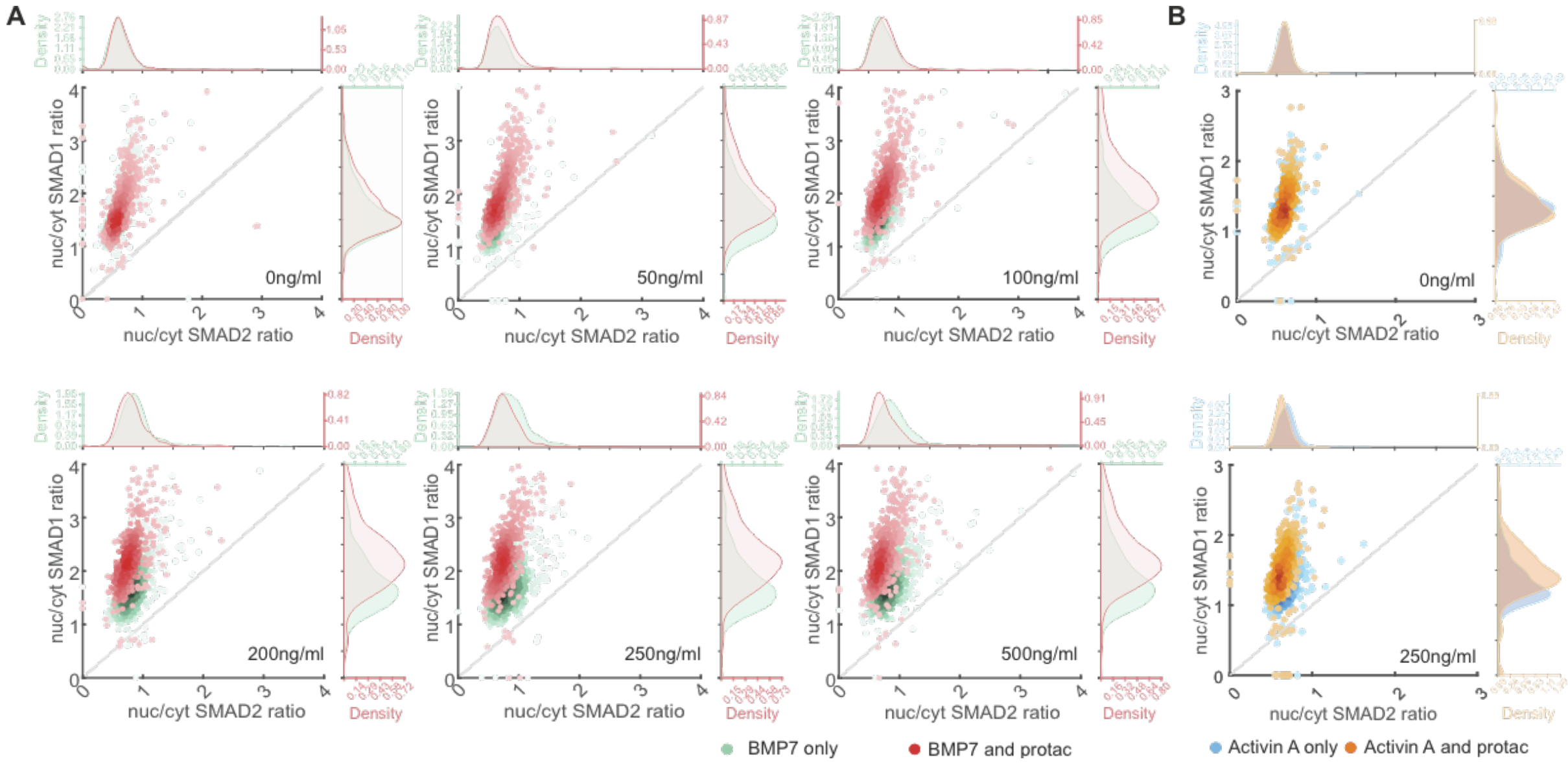

Figure S8. Correlation of SMAD1 and SMAD2 nuclear translocation dynamics. (A-B) Scatter plots of peak nuc/cyt ratios for SMAD2 and SMAD1 in individual cells treated with the indicated doses of BMP7 (A) or Activin A (B) with and without FKBP12 PROTAC. Each dot represents a single cell. The histograms on the axes show the marginal distribution for each condition as smoothed kernel-density estimations. **Note:** Cells were cultured in 1.5% FBS medium with or without FKBP12 PROTAC for 24h prior to stimulation. Ligands (BMP7 or Activin A, 250 ng/ml) were added at 0.5h. Images were captured for 16h. Data is representative for three (A) or five (B) biological repeats.

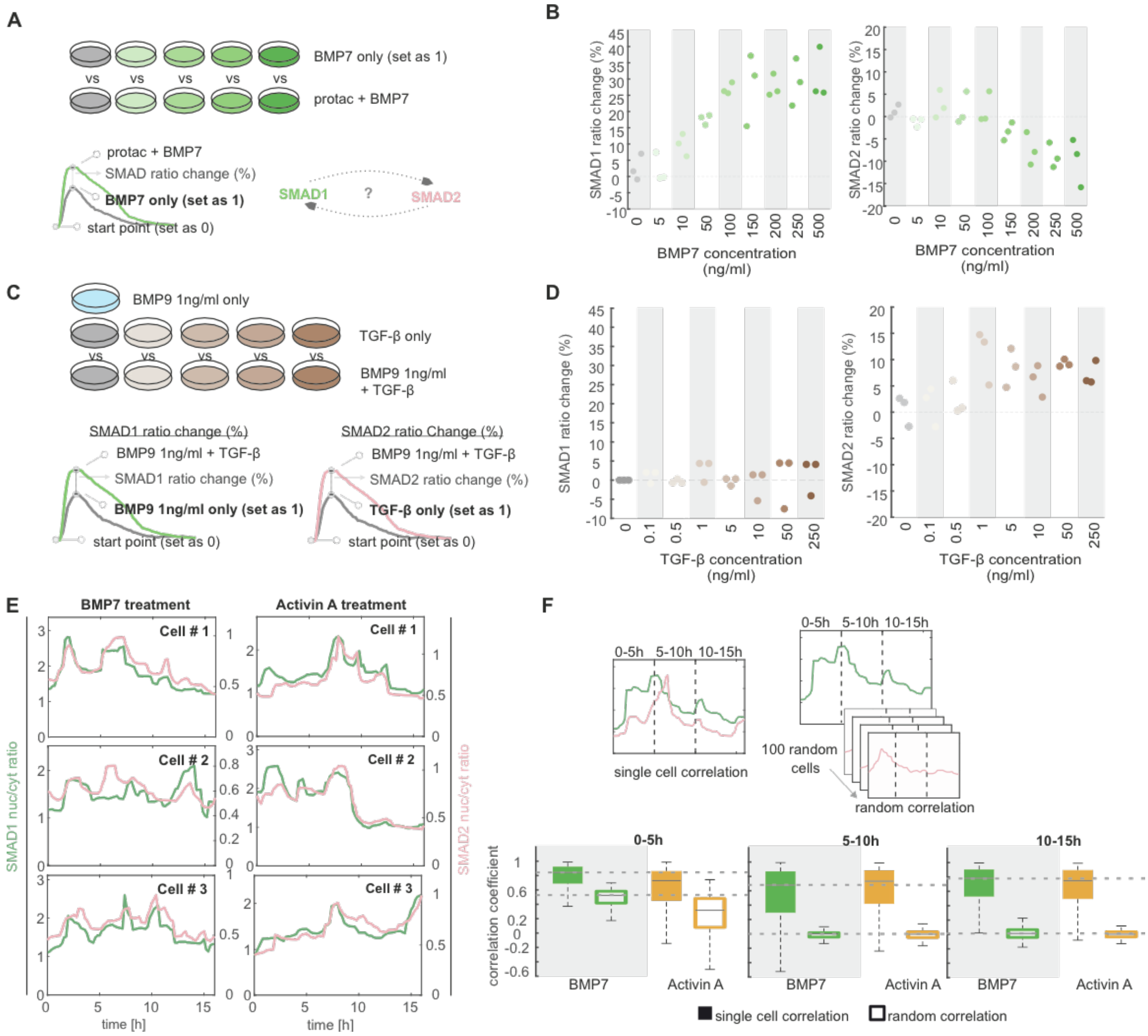

Figure S9. Balance of SMAD1 and SMAD2 activation upon ligand stimulation. (A-B) Median SMAD1 and SMAD2 response ratios were measured in the presence or absence of FKBP12 across BMP7 concentrations. Peak ratios in the presence of FKBP12 were set to 1 and the relative differences calculated. **(C-D)** Dose-response analysis of TGF-β concentrations alone and combined with 1 ng/ml BMP9. Normalization based on median SMAD1 and SMAD2 response ratios, with BMP9-only and TGF-β-only treatments as references (set to 1). Dots in (B) and (D) represent data from biological triplicates. Dashed lines serve as guides to the eye. (**E)** Single-cell time courses of nuc/cyt SMAD1 (green) and SMAD2 (pink) ratios upon stimulation with BMP7 (left) or Activin A (right). **(F)** Pearson correlation analysis of SMAD1 and SMAD2 responses across early (0-5h), middle (5-10h), and late (10-15h) phases. Correlations within individual cells are compared to randomized controls. Dashed lines serve as guides to the eye. Data is representative for three biological repeats.

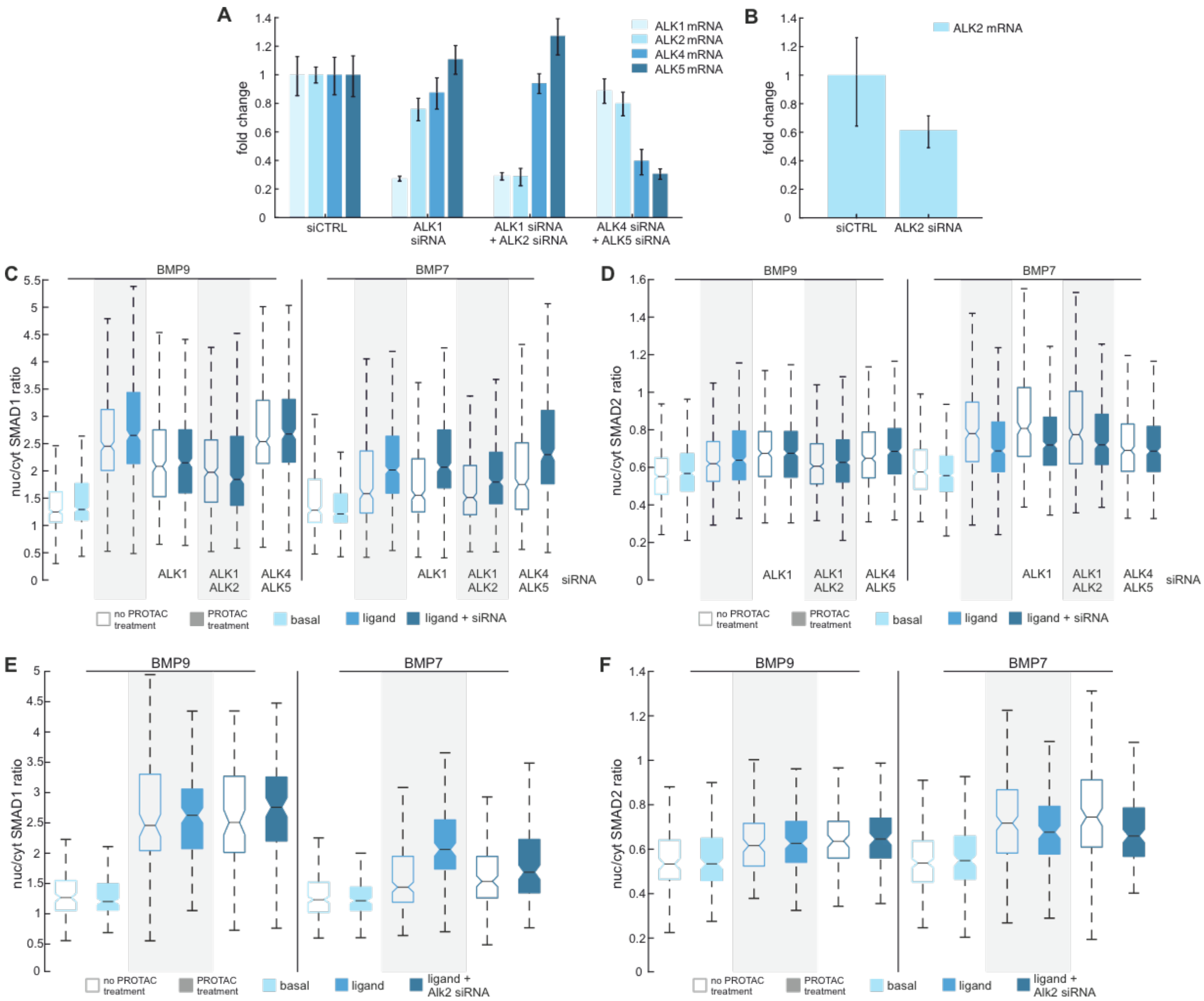

Figure S10: Differential SMAD signaling upon receptor silencing. (A-B) RT-qPCR analysis of receptor expression upon siRNA-mediated silencing. Fold changes were calculated in relation to cells treated with non-targeting siRNA and ß-Actin as a reference. **(C-F)** Peak nuc/cyt ratios of SMAD1 (C, E) and SMAD2 (D, F) upon stimulation with BMP9 (1 ng/ml) or BMP7 (250 ng/ml) over 16 hours. Cells were pre-treated with PROTAC (200nM) and expression of ALK1, 2, 4 and 5 was silenced by siRNA alone or in combination as indicated. Data is representative for two biological repeats.

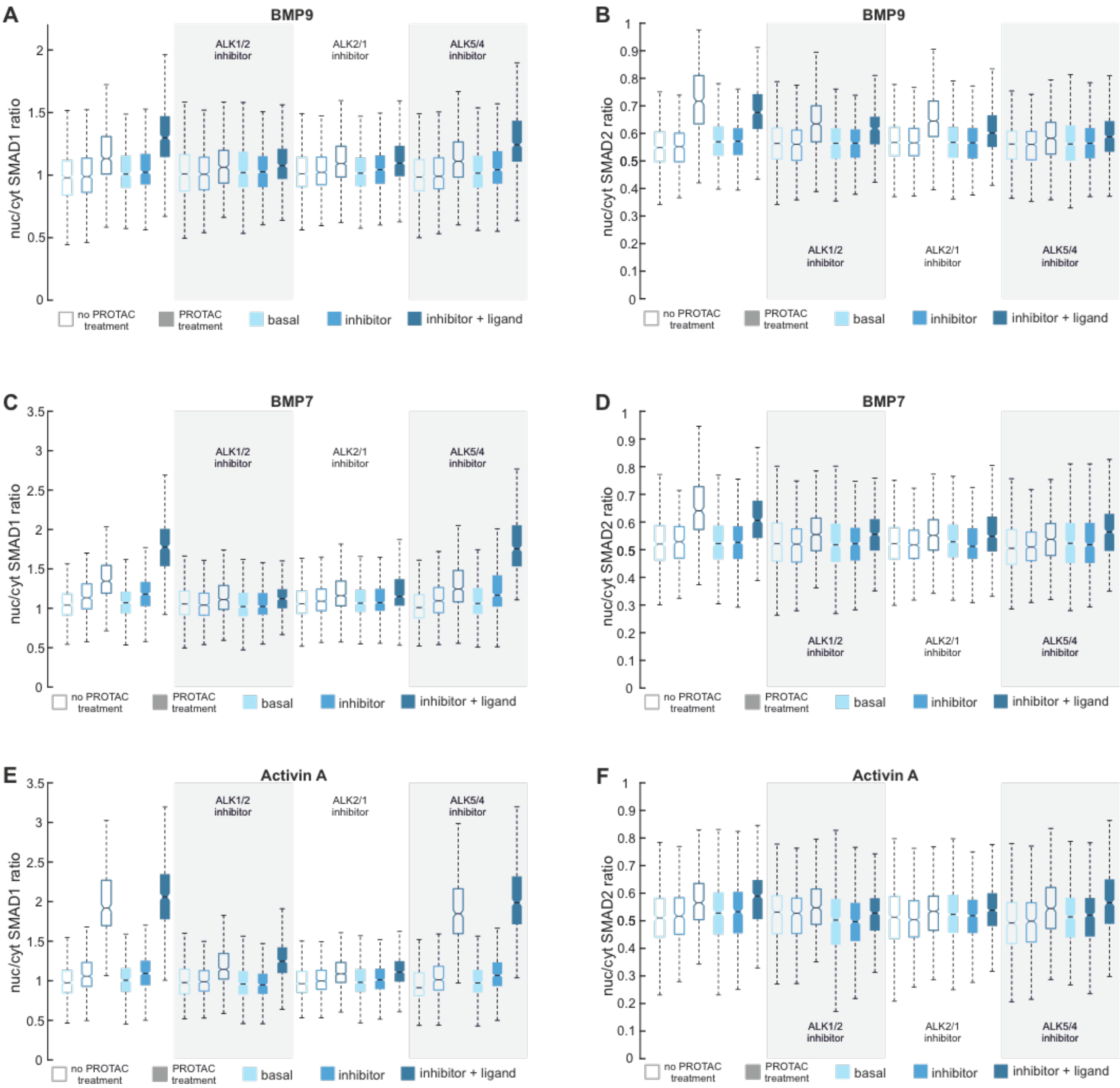

Figure S11: Differential SMAD signaling upon receptor inhibition. (A-F) SMAD1 and SMAD2 responses upon stimulation with (A-B) BMP9 (1 ng/ml), (C-D) BMP7 (250 ng/ml), or (E-F) Activin A (250 ng/ml) over 16 hours. Cells were pre-treated with PROTAC (200nM) and ALK inhibitors ALK1/2i (K02288, 1 µM), ALK2/1i (LDN214117, 10 µM) and ALK5/4i (SB525334, 10 µM) as indicated. Basal nuc/cyt SMAD ratios are compared to those after ALK inhibition alone and post ligand stimulation. When no inhibitor is indicated, DMSO was used as control. Data is representative for three (A-D) or five (E-F) biological repeats.

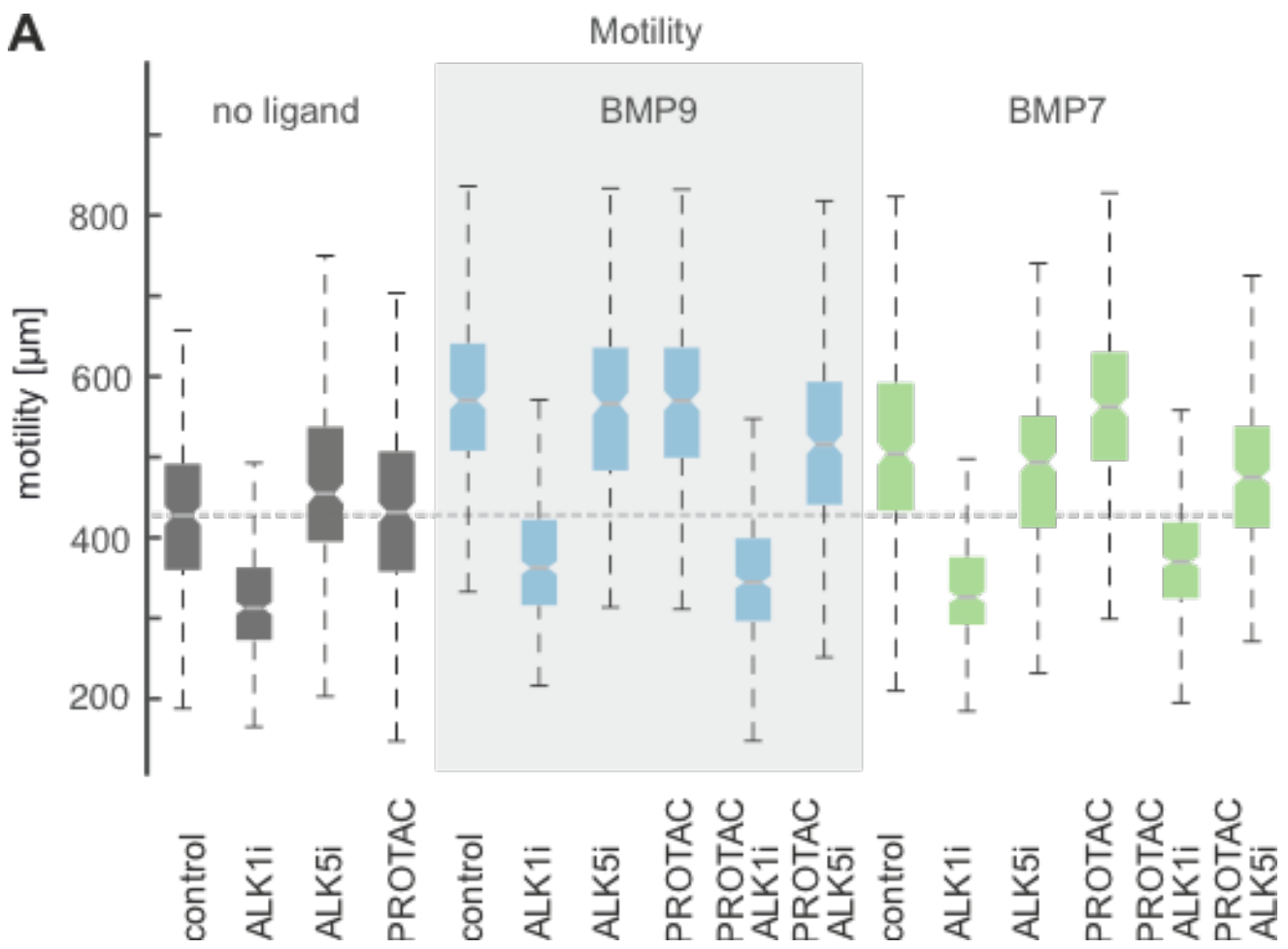

Figure S12: Differential SMAD activation affects cellular motility. Quantification of motility in individual cells following treatment with BMP9 (1 ng/ml) or BMP7 (250 ng/ml). Cells were followed for 48h by time-lapse microscopy and their cumulative displacement quantified. PROTAC (200 nM) was added 24h and ALK1i (1µM) or Alk5/4i (10 µM) were added 1h before stimulation where indicated. **Note:** Cells were cultured in 1.5% FBS medium with or without PROTAC for 24 hours.
