# Supplementary material for: FKBP12 determines ligand-specific SMAD signaling in individual living endothelial cells
Source: iScience. 2026 Sep 22;29(10):117728. doi: 10.1016/j.isci.2026.117728 (PMC13659684; doi:10.1016/j.isci.2026.117728)
Supplement: Supplementary file 2 [file mmc2.pdf]

Raw western blot images

Figure 1B (time course)

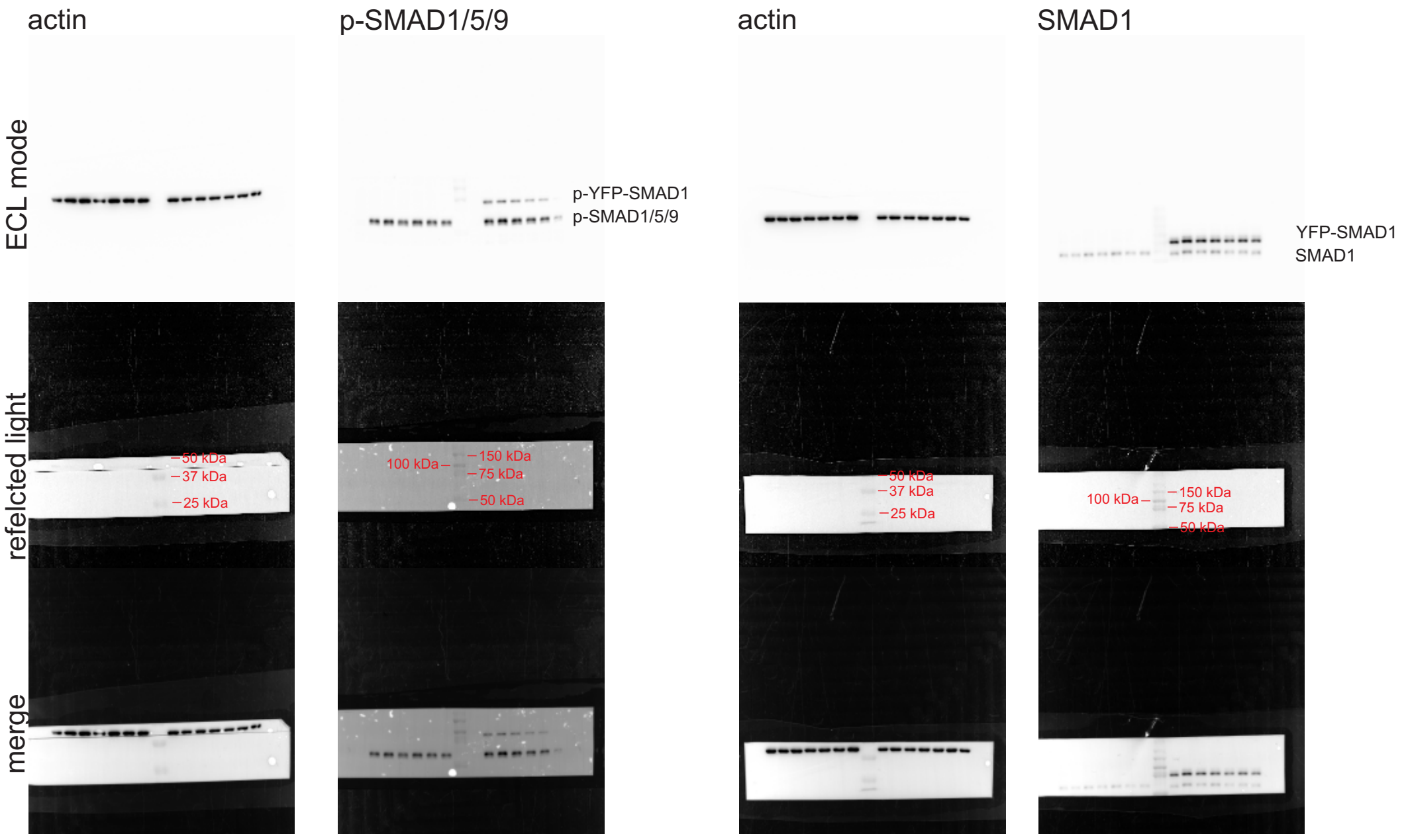

Figure 1B (titration)

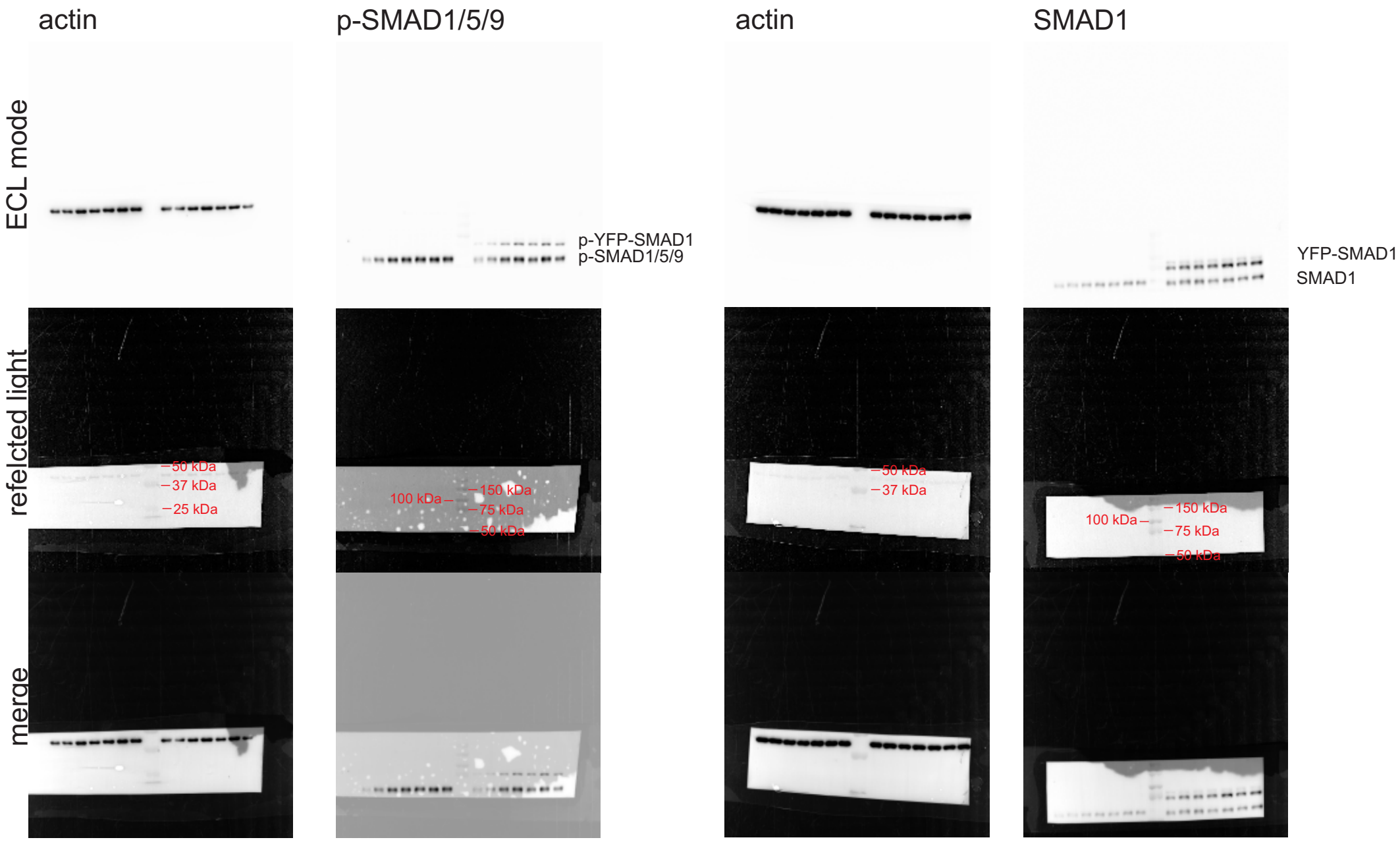

Figure 3A

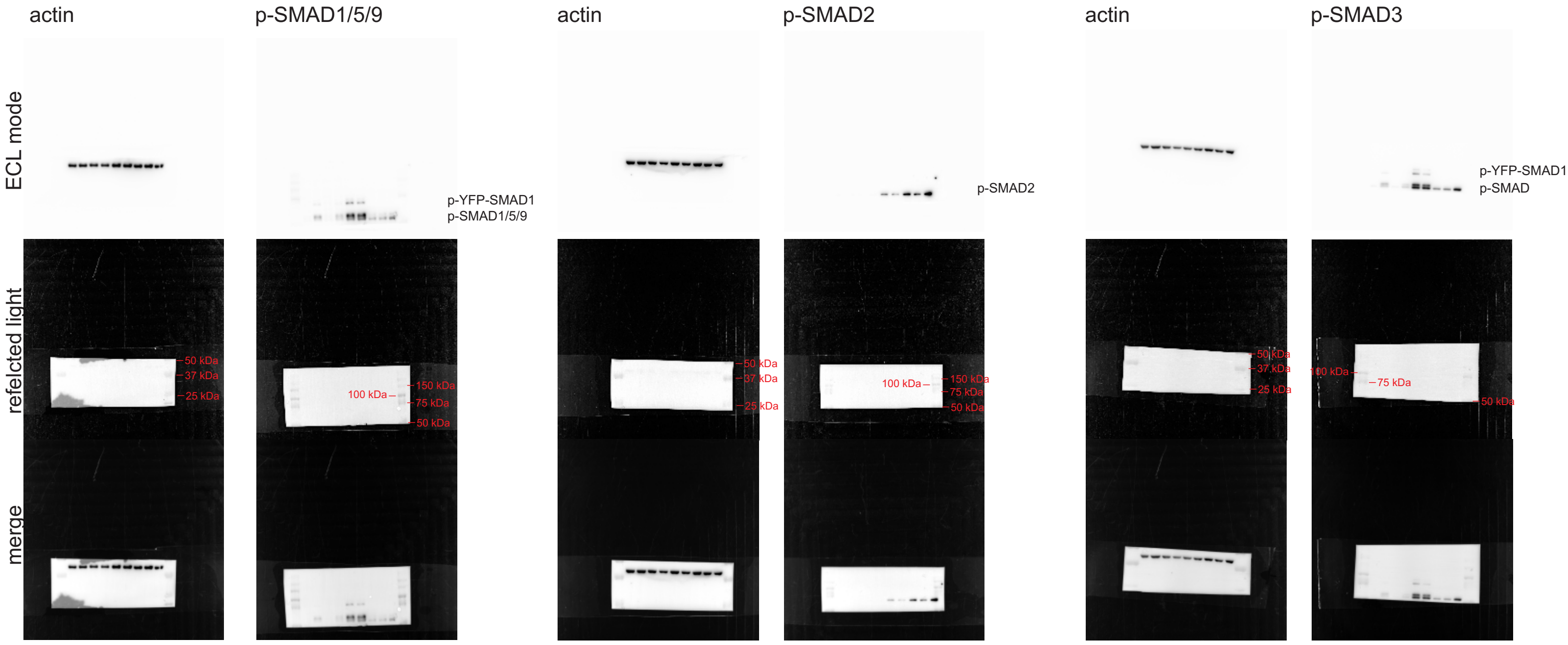

actin
p-SMAD1/5/9
actin
p-SMAD2
actin
p-SMAD3
ECL mode
refelcted light
merge
p-YFP-SMAD1
p-SMAD1/5/9
p-SMAD2
p-YFP-SMAD1
p-SMAD
50 kDa
37 kDa
25 kDa
100 kDa
150 kDa
75 kDa
50 kDa
50 kDa
37 kDa
25 kDa
100 kDa
150 kDa
75 kDa
50 kDa
50 kDa
37 kDa
25 kDa
75 kDa
50 kDa

Figure 4B (titration)

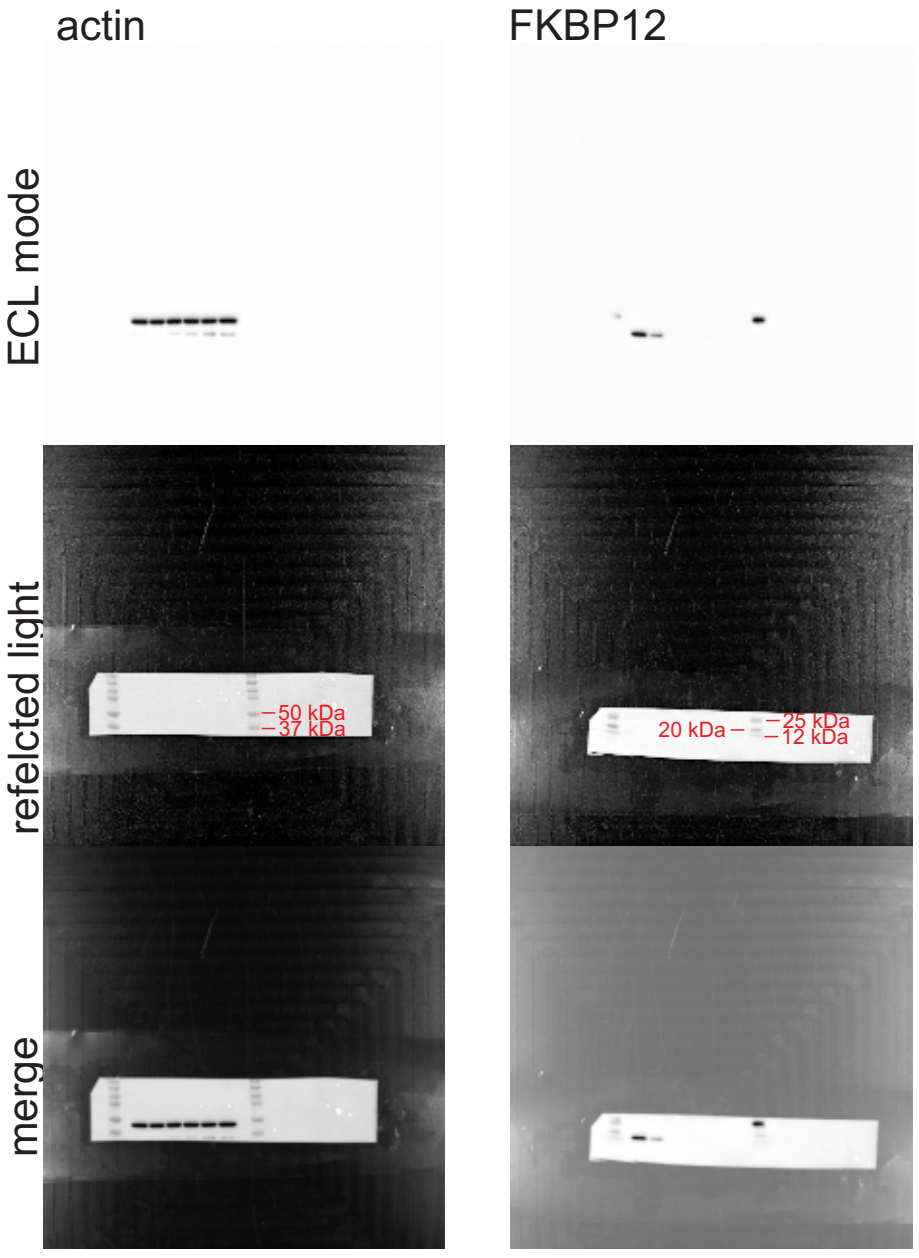

Figure 4B (time course)

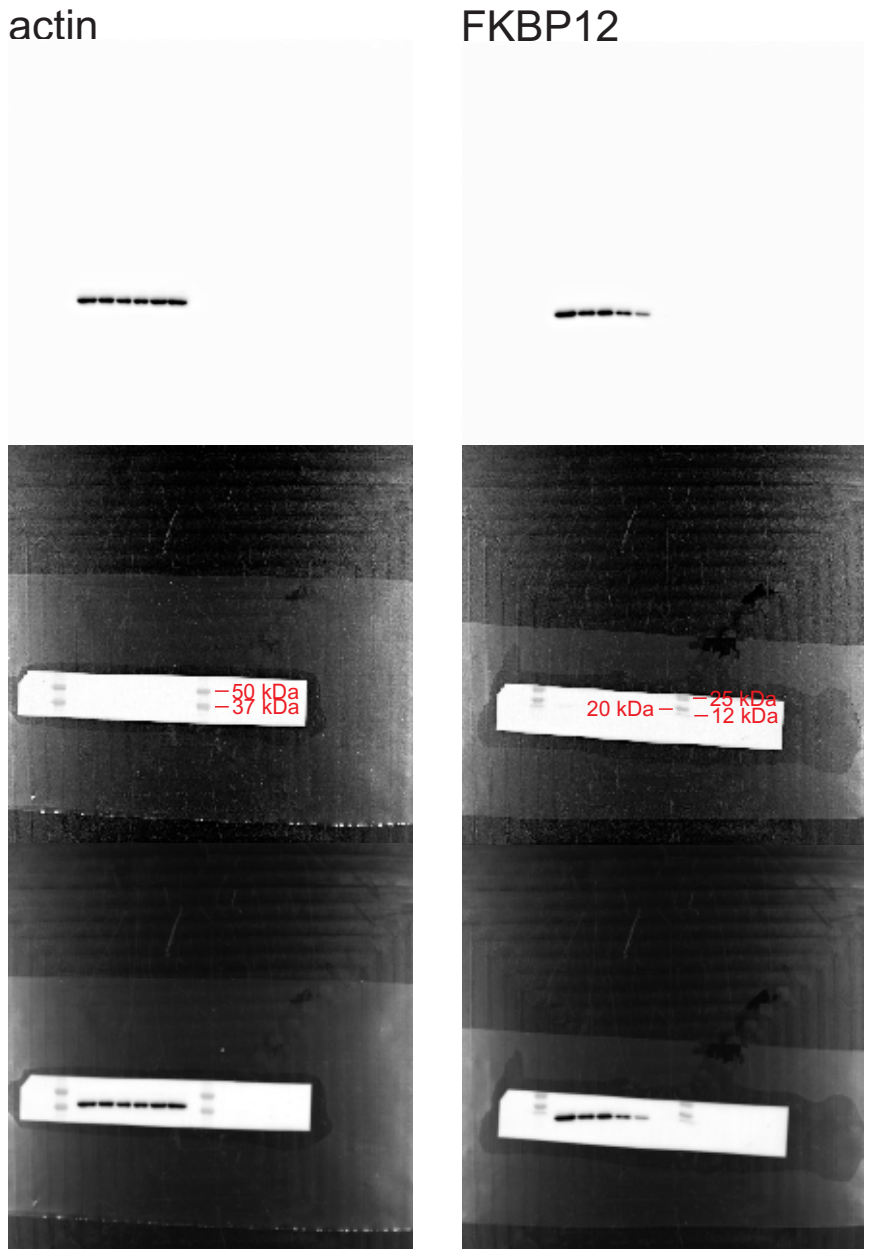

Figure 4D (left column)

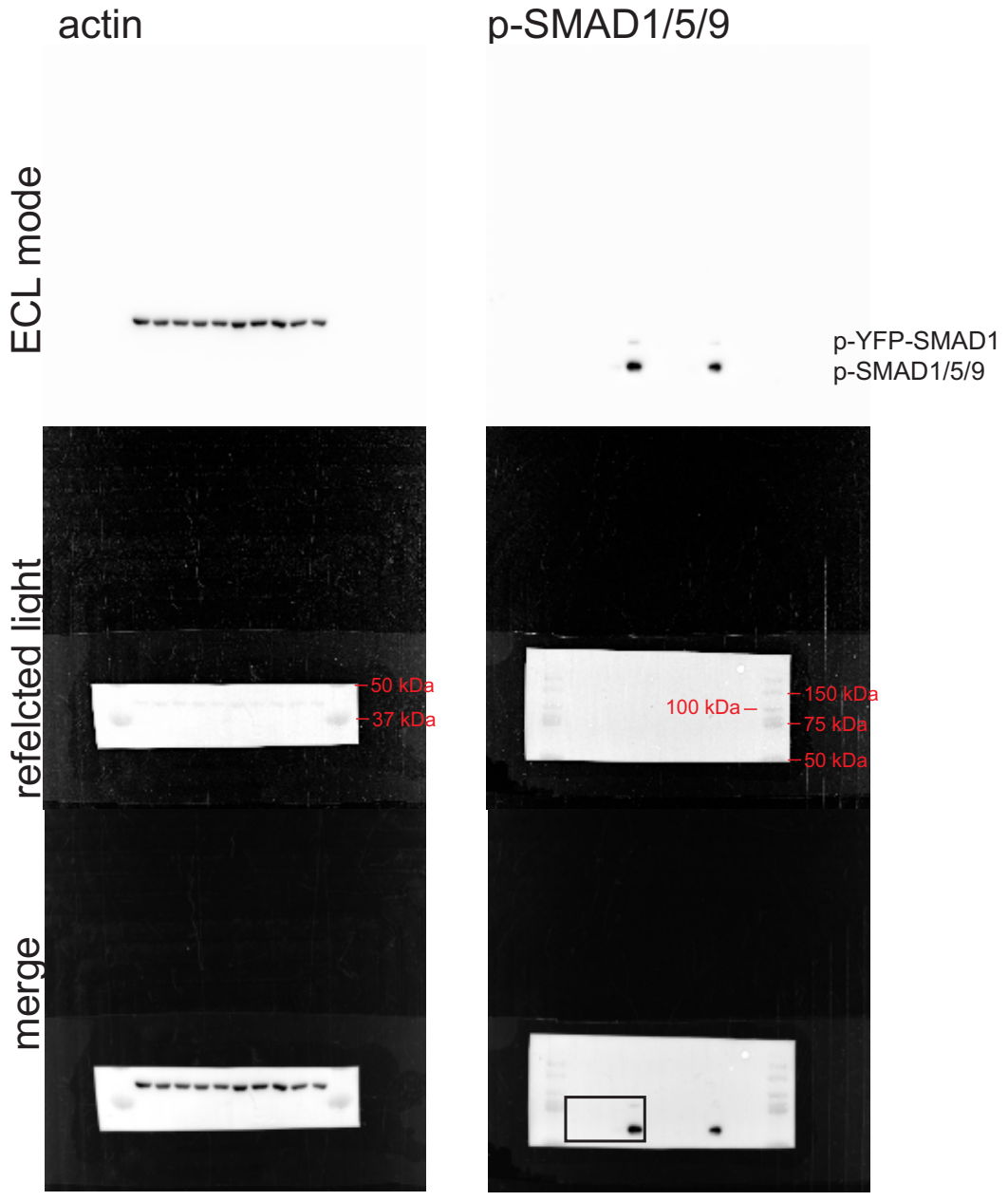

Figure 4D (right column)

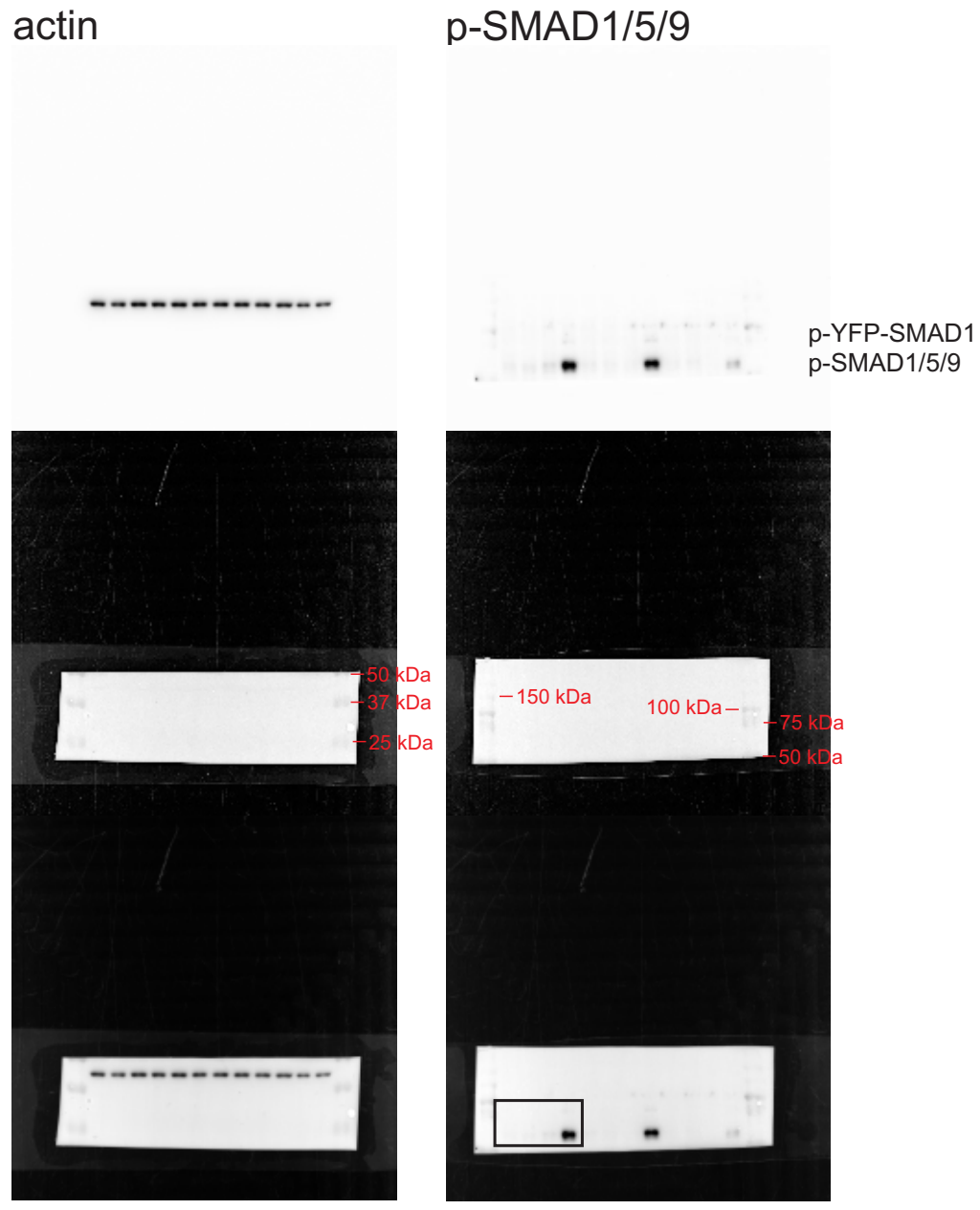

Figure S2A

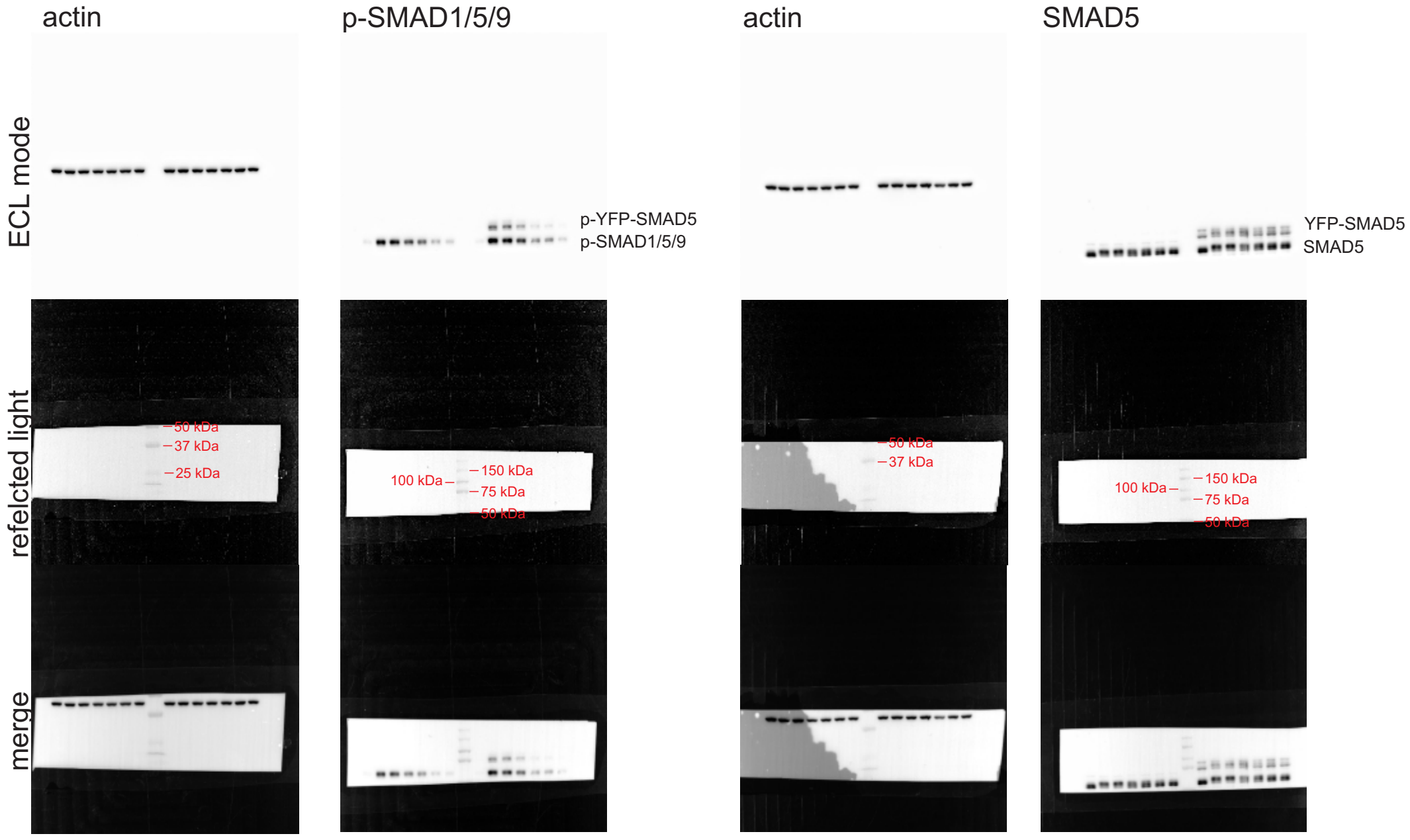
actin
p-SMAD1/5/9
actin
SMAD5
ECL mode
refelcted light
merge
p-YFP-SMAD5
p-SMAD1/5/9
YFP-SMAD5
SMAD5
50 kDa
37 kDa
25 kDa
100 kDa
150 kDa
75 kDa
50 kDa

Figure S2B

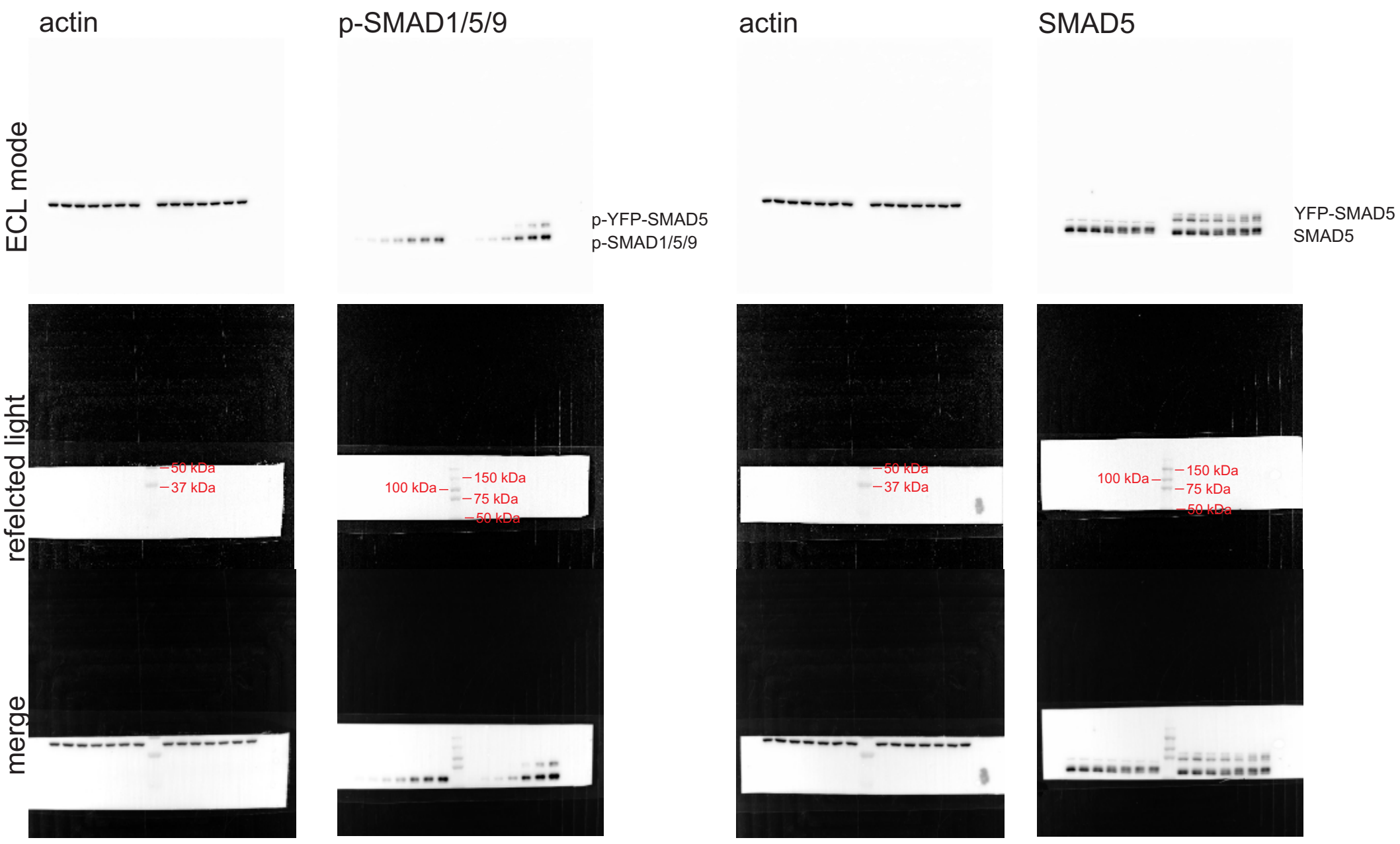
actin
p-SMAD1/5/9
actin
SMAD5
ECL mode
refelcted light
merge
p-YFP-SMAD5
p-SMAD1/5/9
YFP-SMAD5
SMAD5
50 kDa
37 kDa
100 kDa
150 kDa
75 kDa
50 kDa

Figure S3A

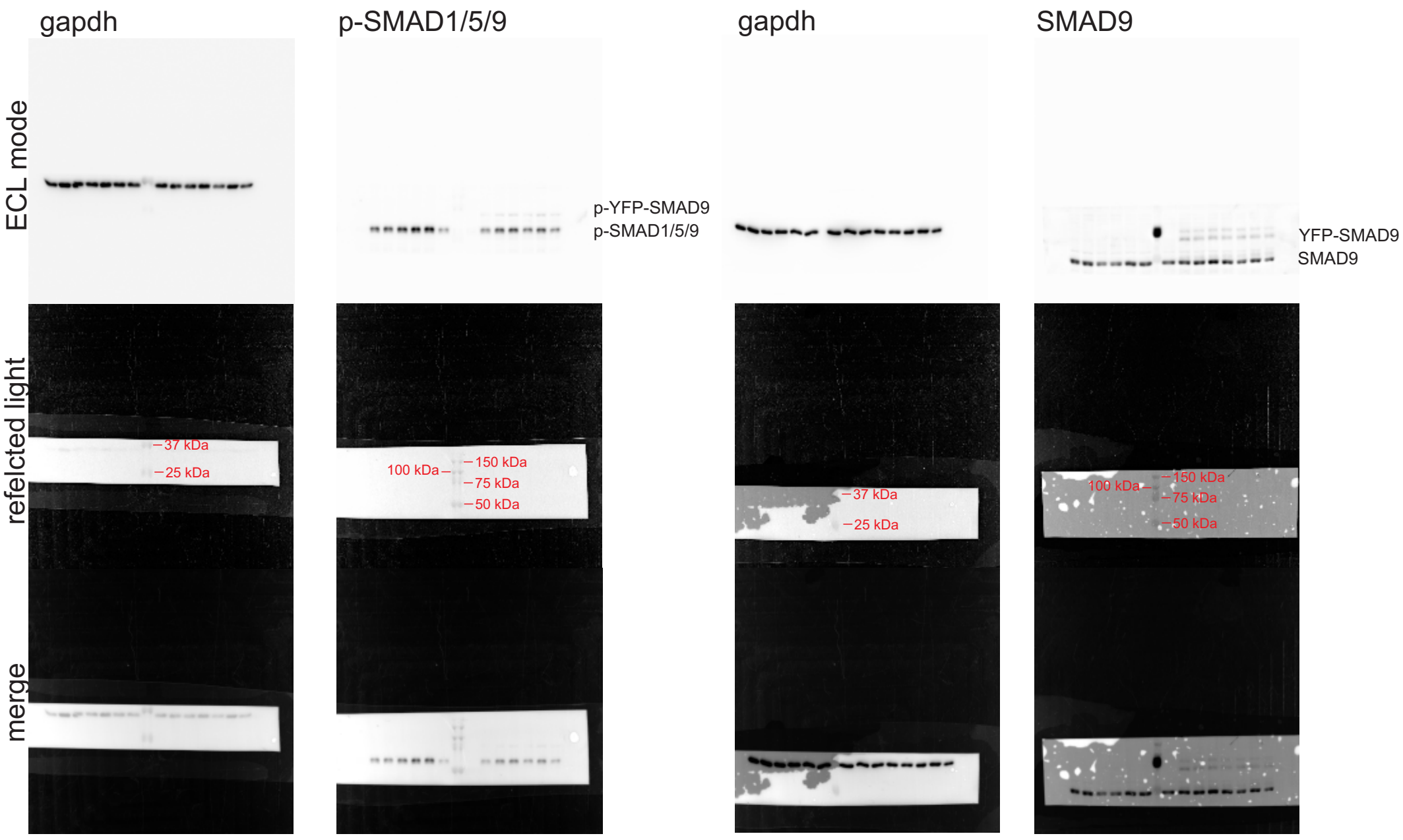
gapdh
p-SMAD1/5/9
gapdh
SMAD9
ECL mode
refelcted light
merge
p-YFP-SMAD9
p-SMAD1/5/9
YFP-SMAD9
SMAD9
37 kDa
25 kDa
100 kDa
150 kDa
75 kDa
50 kDa
37 kDa
25 kDa
100 kDa
150 kDa
75 kDa
50 kDa

Figure S5G

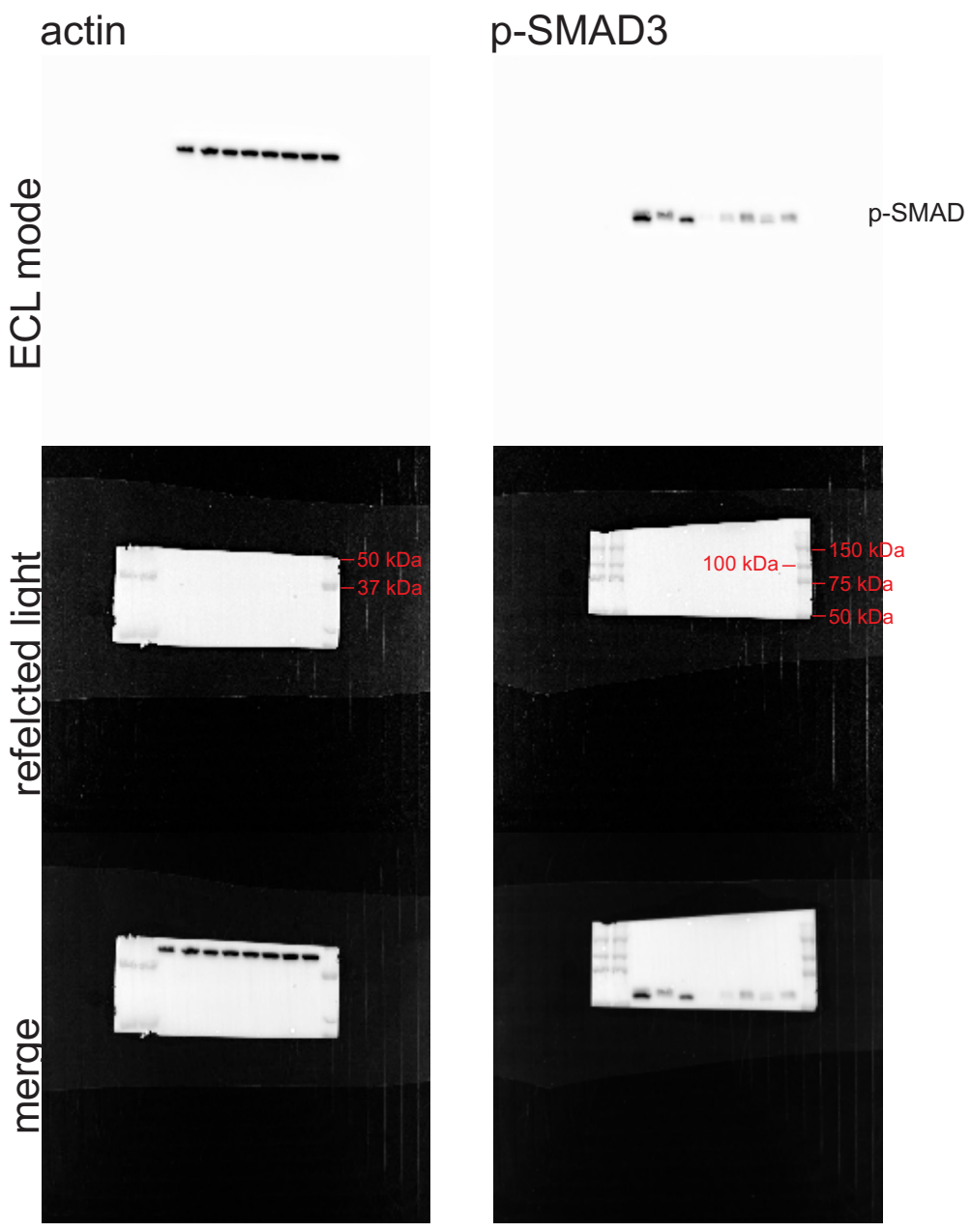
actin
p-SMAD3
ECL mode
refelcted light
merge
p-SMAD
50 kDa
37 kDa
150 kDa
100 kDa
75 kDa
50 kDa

Figure S7A

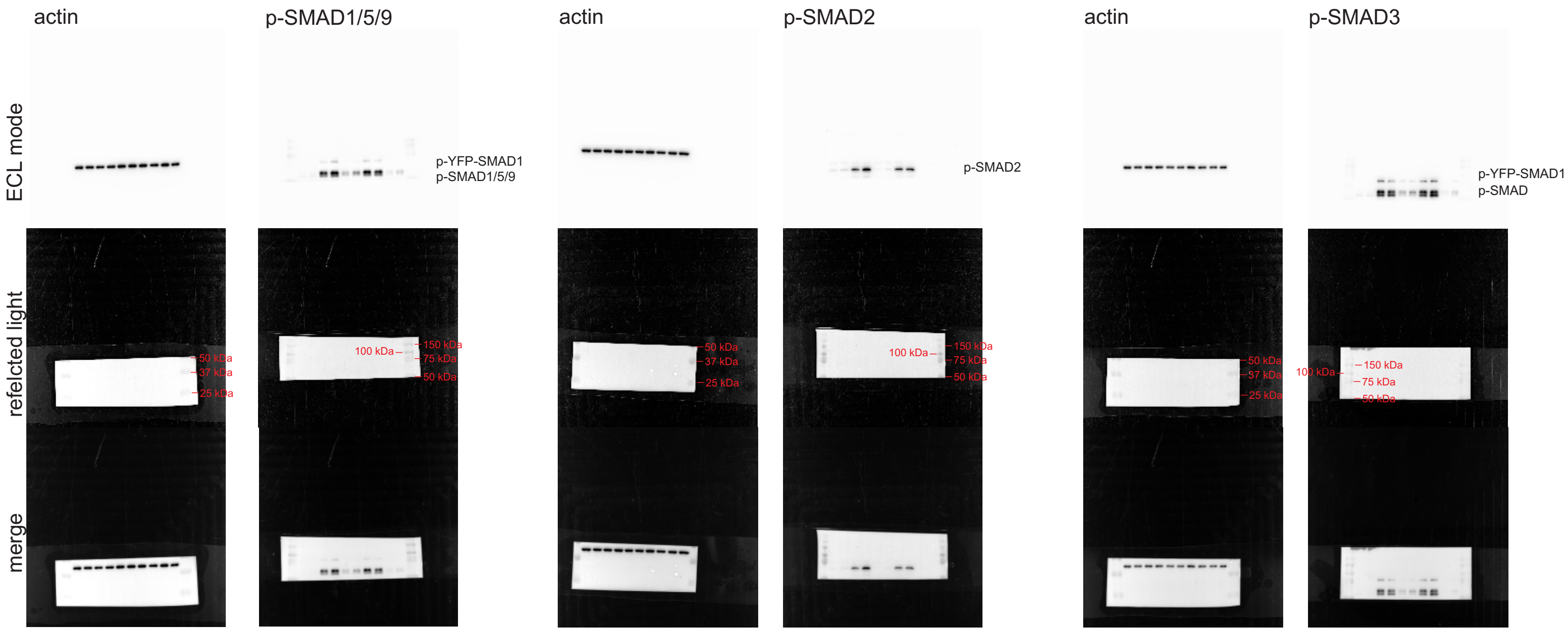
actin
p-SMAD1/5/9
actin
p-SMAD2
actin
p-SMAD3
ECL mode
refelcted light
merge
p-YFP-SMAD1
p-SMAD1/5/9
p-SMAD2
p-YFP-SMAD1
p-SMAD
50 kDa
37 kDa
25 kDa
100 kDa
150 kDa
75 kDa
50 kDa

Figure S7B

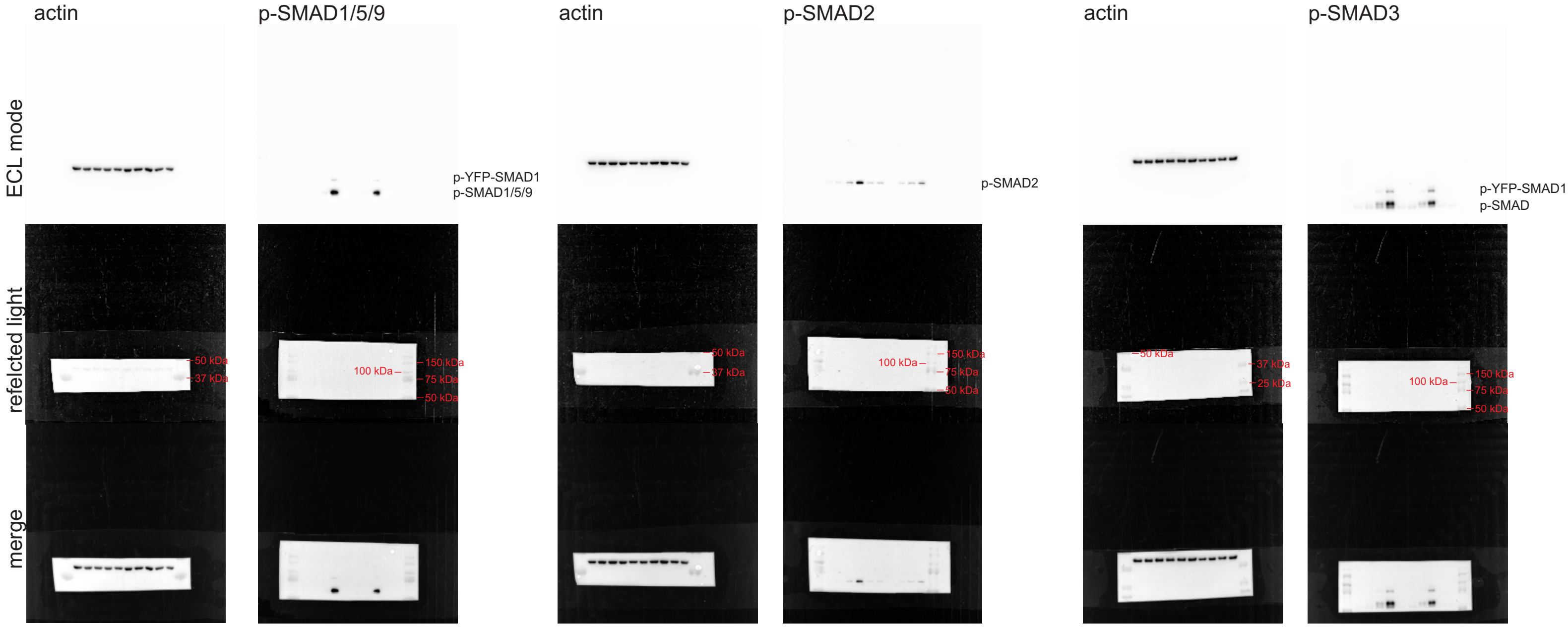
actin
p-SMAD1/5/9
actin
p-SMAD2
actin
p-SMAD3
ECL mode
refelcted light
merge
p-YFP-SMAD1
p-SMAD1/5/9
p-SMAD2
p-YFP-SMAD1
p-SMAD
50 kDa
37 kDa
100 kDa
150 kDa
75 kDa
50 kDa
50 kDa
37 kDa
100 kDa
150 kDa
75 kDa
50 kDa
50 kDa
37 kDa
25 kDa
100 kDa
150 kDa
75 kDa
50 kDa

Figure S7B

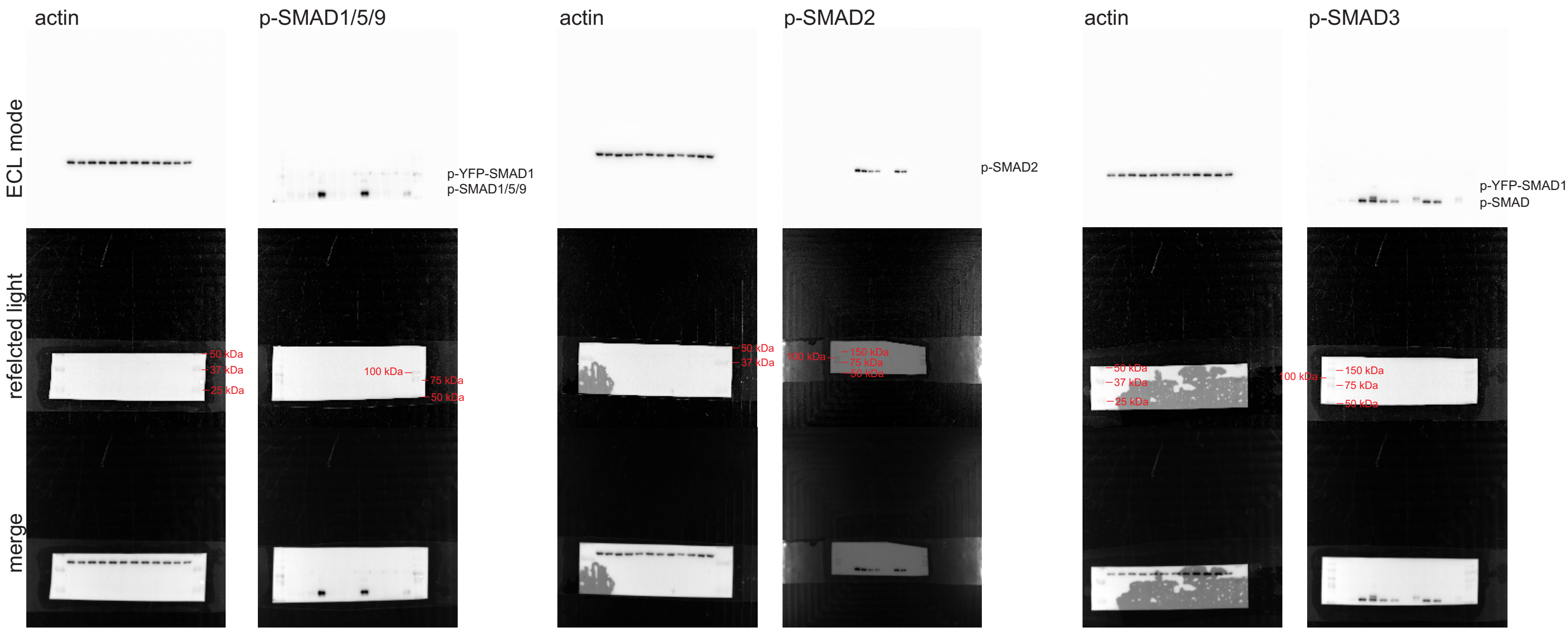
actin
p-SMAD1/5/9
actin
p-SMAD2
actin
p-SMAD3
ECL mode
refelcted light
merge
p-YFP-SMAD1
p-SMAD1/5/9
p-SMAD2
p-YFP-SMAD1
p-SMAD
50 kDa
37 kDa
25 kDa
100 kDa
75 kDa
50 kDa
50 kDa
37 kDa
100 kDa
150 kDa
75 kDa
50 kDa
50 kDa
37 kDa
25 kDa
100 kDa
150 kDa
75 kDa
50 kDa
