# Supplementary material for: FKBP12 determines ligand-specific SMAD signaling in individual living endothelial cells
Source: iScience. 2026 Sep 22;29(10):117728. doi: 10.1016/j.isci.2026.117728 (PMC13659684; doi:10.1016/j.isci.2026.117728)

Western Blot Raw Data

Figure 1B (time course)

repeat 1

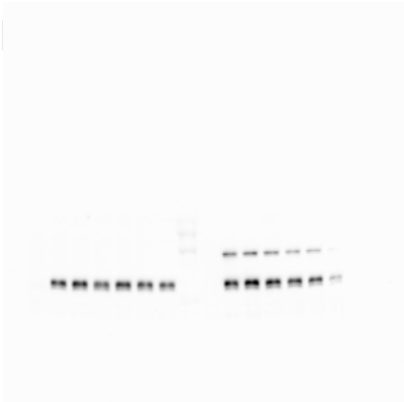

repeat 2

actin

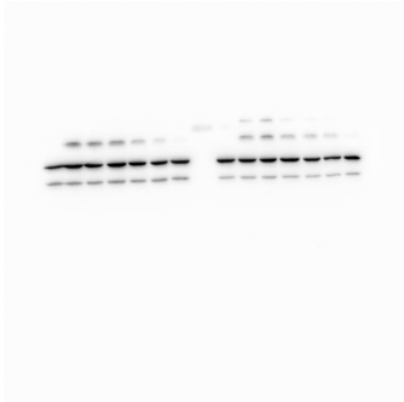

p-SMAD1/5/9

repeat 3

actin

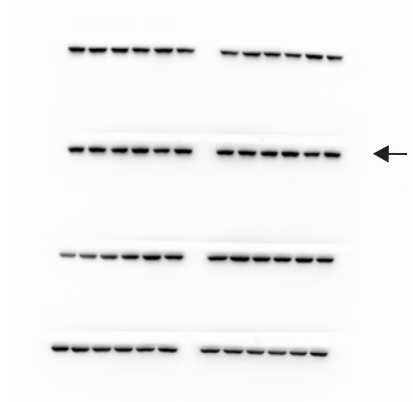

p-SMAD1/5/9

repeat 1

actin

SMAD1

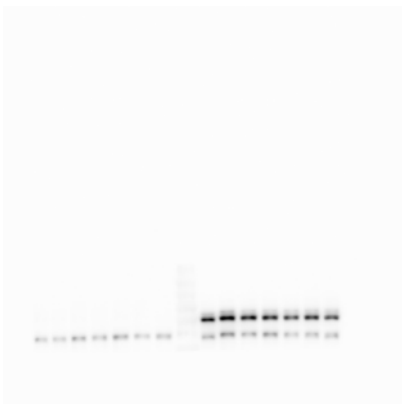

repeat 2

actin

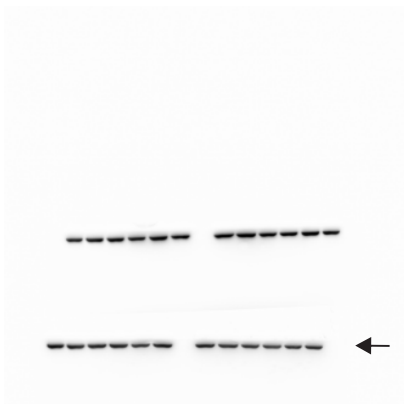

SMAD1

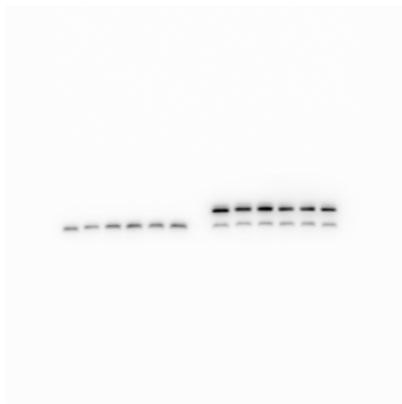

repeat 3

actin

SMAD1

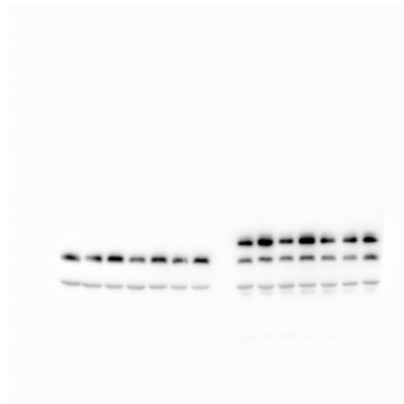

Figure 1B (titration)

repeat 1

actin

p-SMAD1/5/9

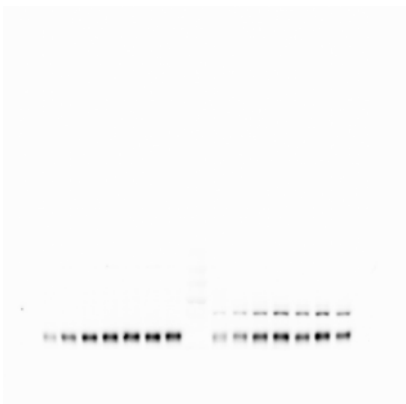

repeat 2

actin

p-SMAD1/5/9

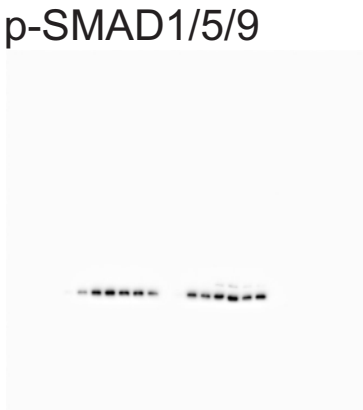

repeat 3

actin / p-SMAD1/5/9

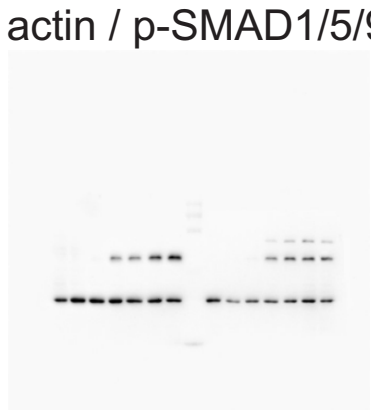

repeat 1

actin

SMAD1

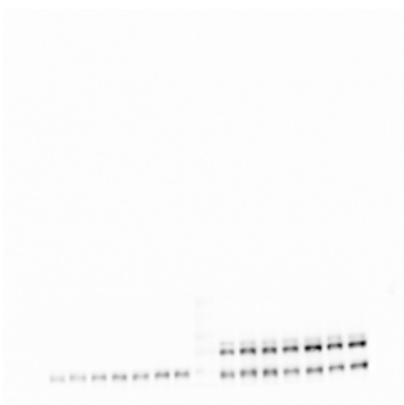

repeat 2

actin

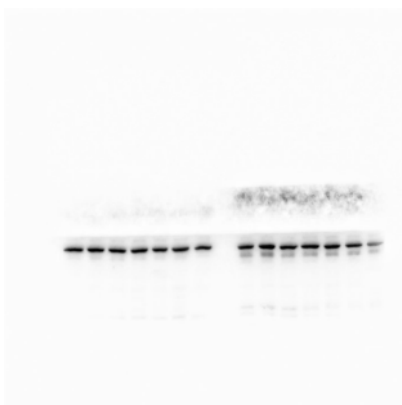

SMAD1

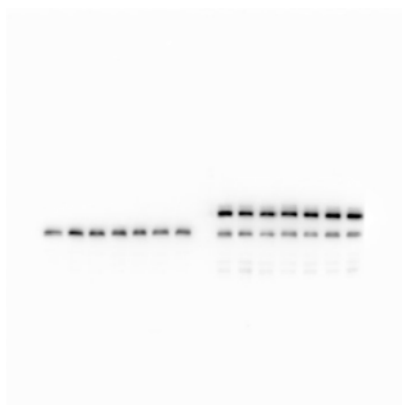

repeat 3

actin

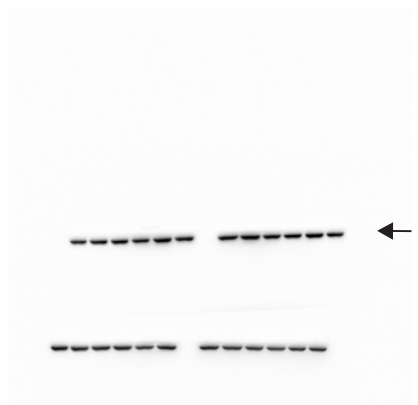

SMAD1

Western Blot Raw Data

Figure 3A

repeat 1

actin

p-SMAD1/5/9

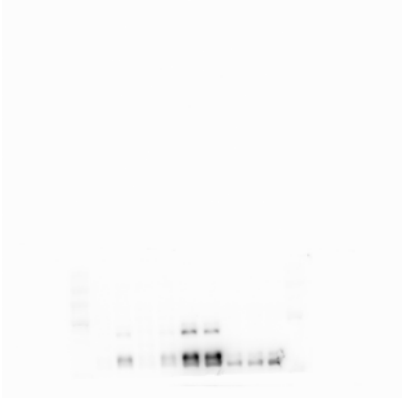

repeat 2

actin

p-SMAD1/5/9

repeat 3

actin

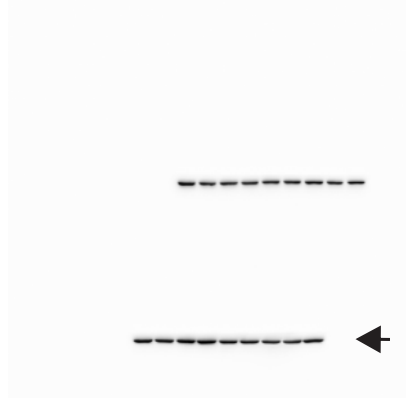

p-SMAD1/5/9

repeat 1

actin

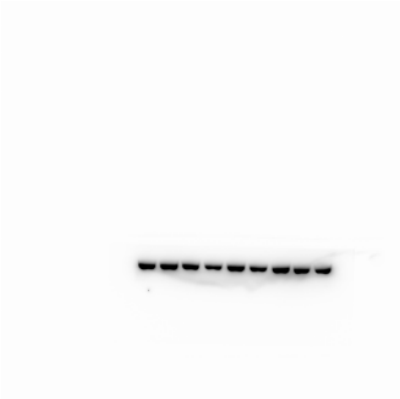

p-SMAD2

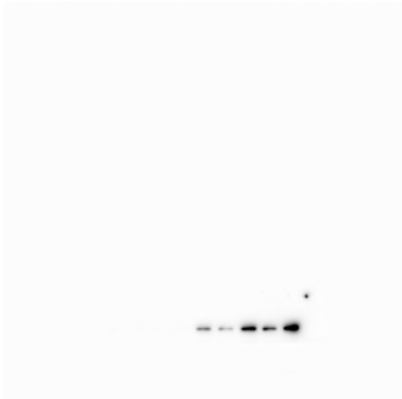

repeat 2

actin

p-SMAD2

repeat 3

actin

p-SMAD2

repeat 1

actin

p-SMAD3

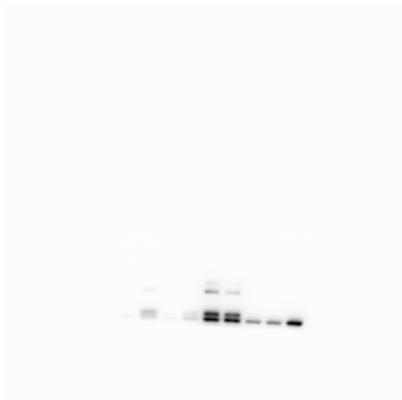

repeat 2

actin

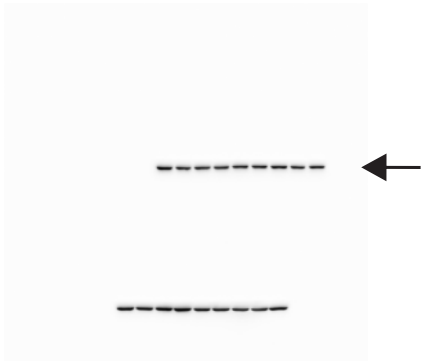

p-SMAD3

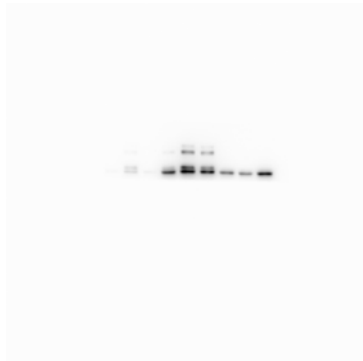

repeat 3

actin

p-SMAD3

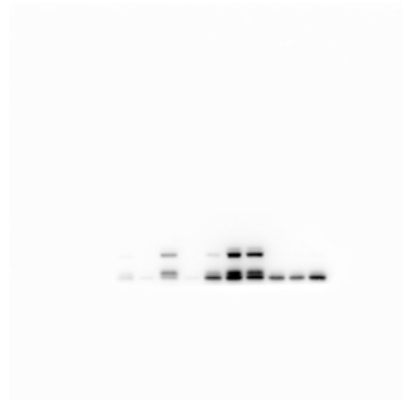

Western Blot Raw Data

Figure 4B (titration)

repeat 1

actin

FKBP12

repeat 2

actin

FKBP12

Figure 4B (time course)

repeat 1

actin

FKBP12

repeat 2

actin

FKBP12

Western Blot Raw Data

Figure 4D (upper blot)

repeat 1

actin

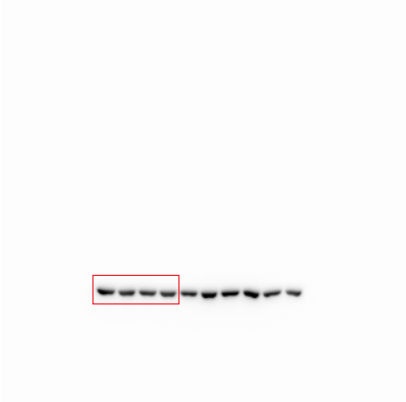

p-SMAD1/5/9

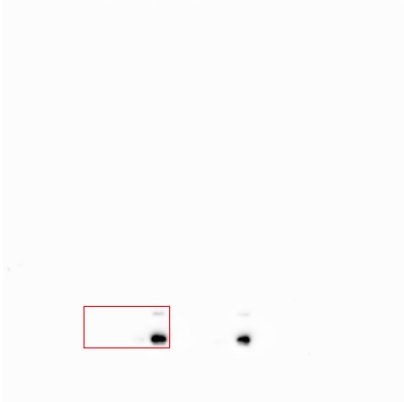

repeat 2

actin

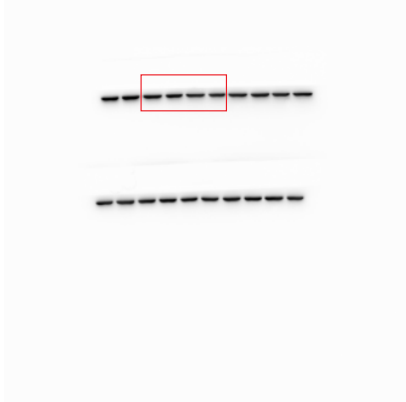

p-SMAD1/5/9

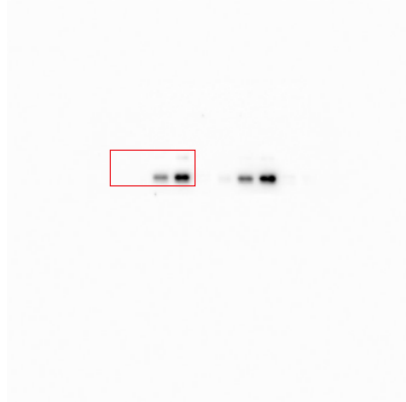

Figure 4D (lower blot)

repeat 1

actin

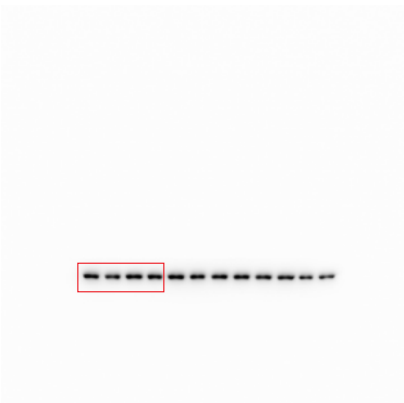

p-SMAD1/5/9

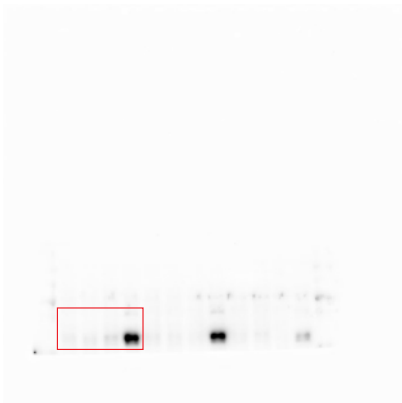

repeat 2

actin

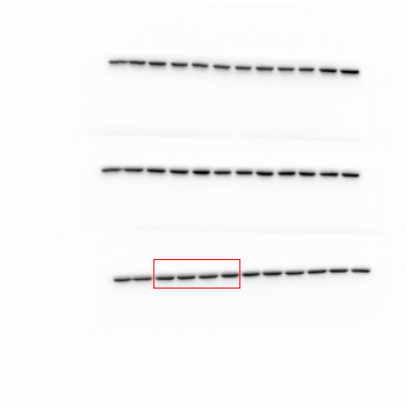

p-SMAD1/5/9

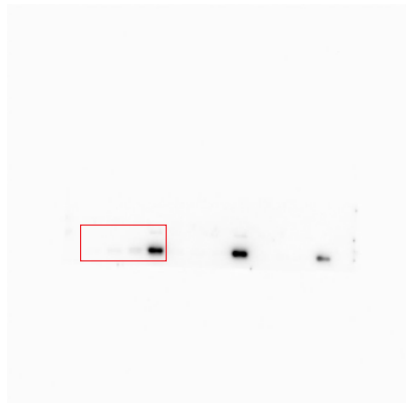

Western Blot Raw Data

Figure S2A

repeat 1

actin

p-SMAD1/5/9

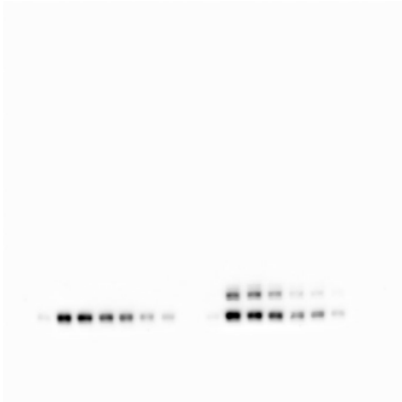

repeat 2

actin

p-SMAD1/5/9

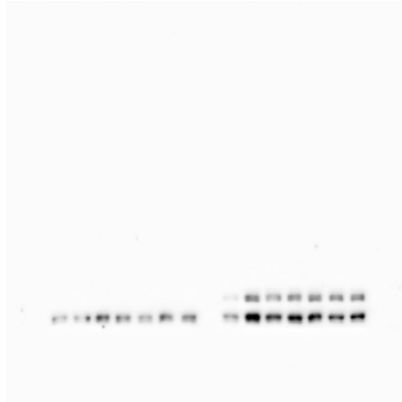

repeat 3

actin

p-SMAD1/5/9

repeat 1

actin

SMAD5

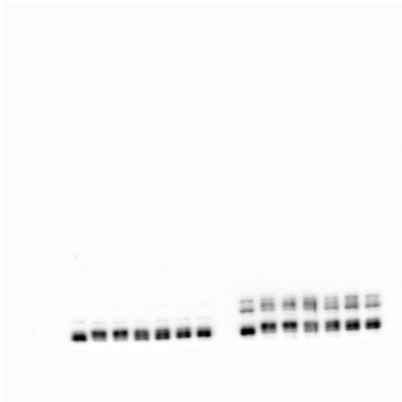

repeat 2

actin

SMAD5

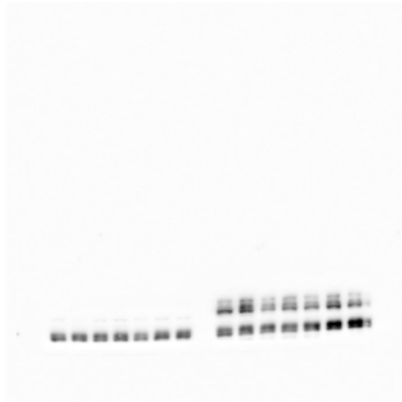

repeat 3

actin

SMAD5

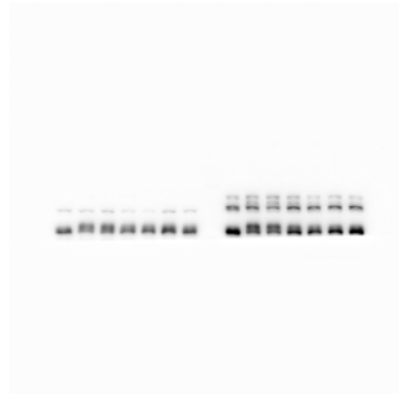

Western Blot Raw Data

Figure S2B

repeat 1

actin

p-SMAD1/5/9

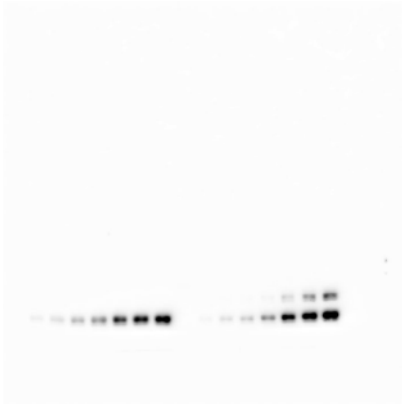

repeat 2

actin

p-SMAD1/5/9

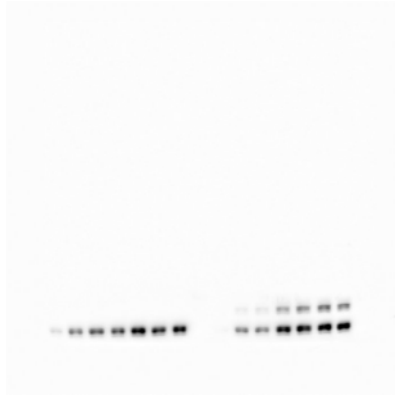

repeat 3

actin

p-SMAD1/5/9

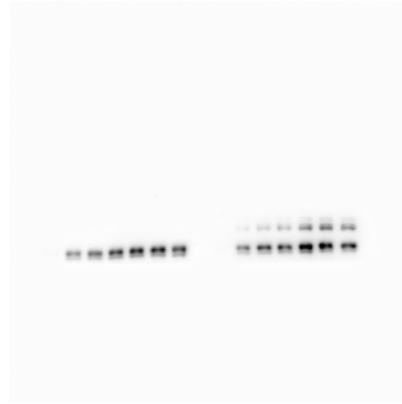

repeat 1

actin

SMAD5

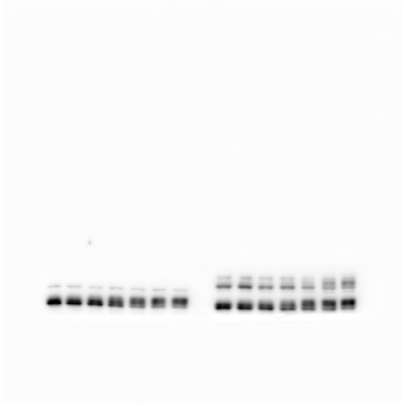

repeat 2

actin

SMAD5

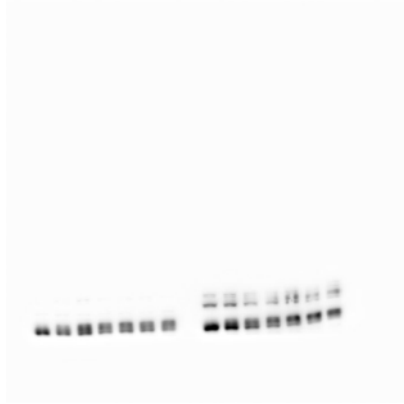

repeat 3

actin

SMAD5

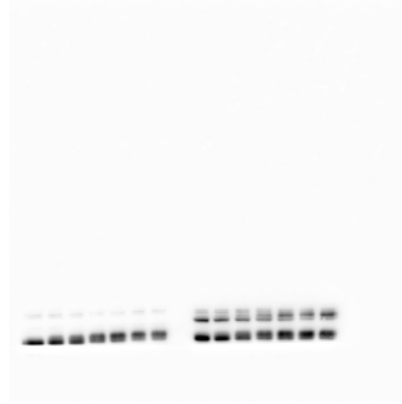

Western Blot Raw Data

Figure S3A

repeat 1

GAPDH

p-SMAD1/5/9

repeat 2

actin

p-SMAD1/5/9

repeat 3

actin

p-SMAD1/5/9

repeat 1

GAPDH

SMAD9

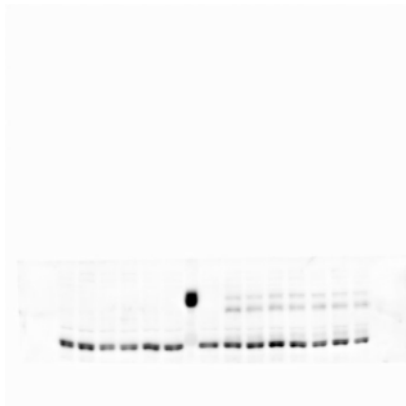

repeat 2

actin

SMAD9

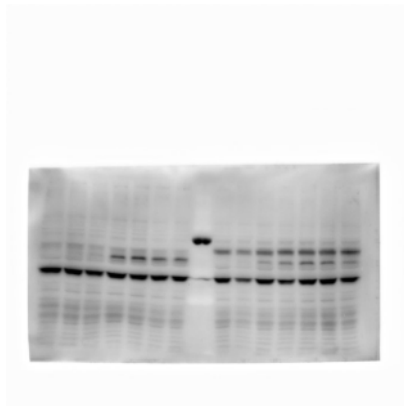

Western Blot Raw Data

Figure S5G

actin

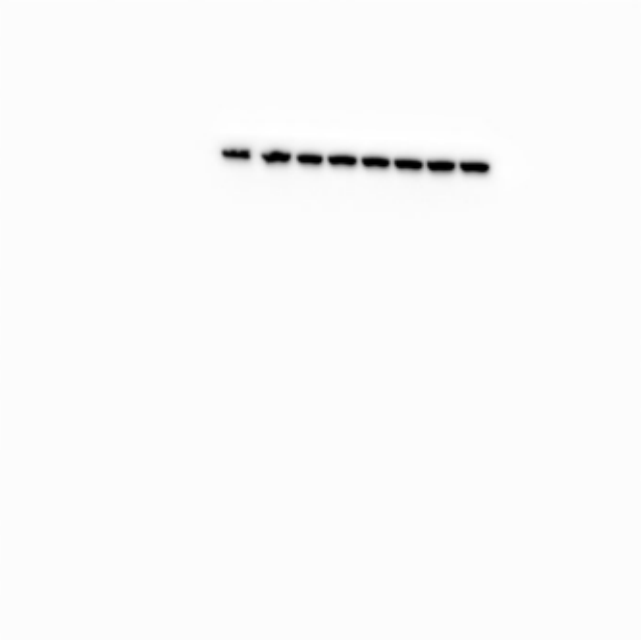

p-SMAD3

Western Blot Raw Data

Figure S7A

repeat 1

actin

p-SMAD1/5/9

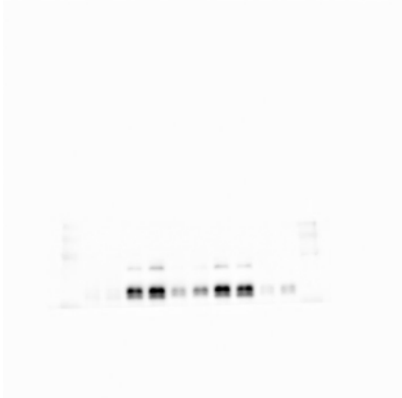

repeat 2

actin

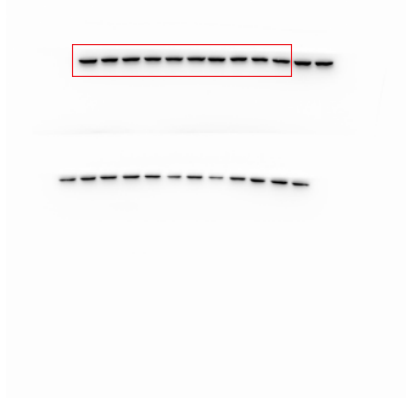

p-SMAD1/5/9

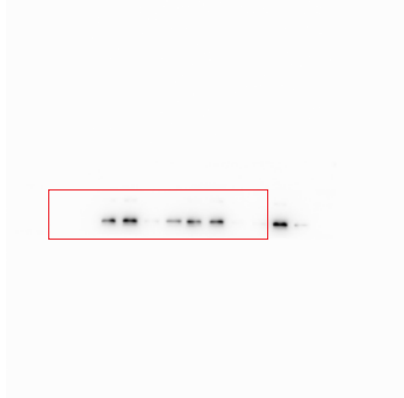

repeat 3

actin

p-SMAD1/5/9

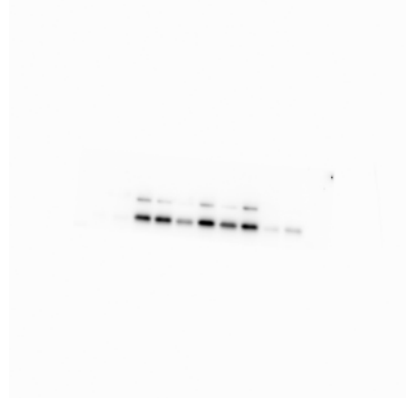

repeat 1

actin

p-SMAD2

repeat 2

actin

p-SMAD2

repeat 3

actin

p-SMAD2

repeat 1

actin

p-SMAD3

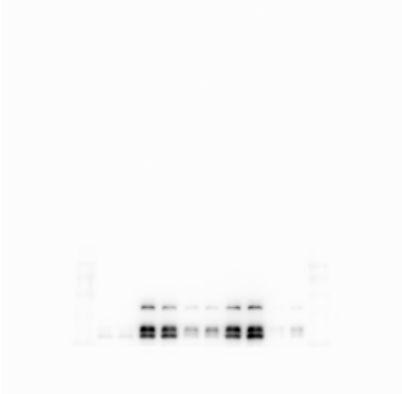

repeat 2

actin

p-SMAD3

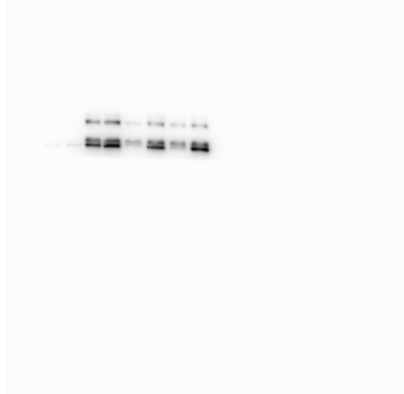

repeat 3

actin

p-SMAD3

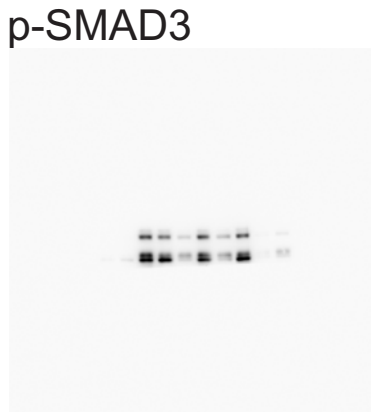

Western Blot Raw Data

Figure S7B

repeat 1

actin

p-SMAD1/5/9

repeat 2

actin

p-SMAD1/5/9

repeat 3

actin

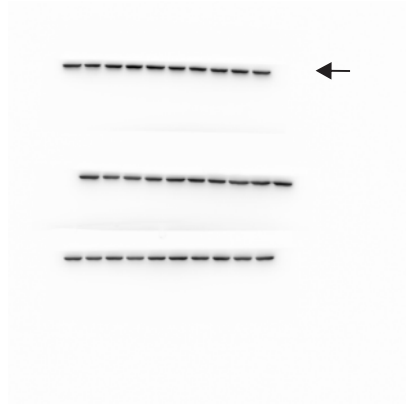

p-SMAD1/5/9

repeat 1

actin

p-SMAD2

repeat 2

actin

p-SMAD2

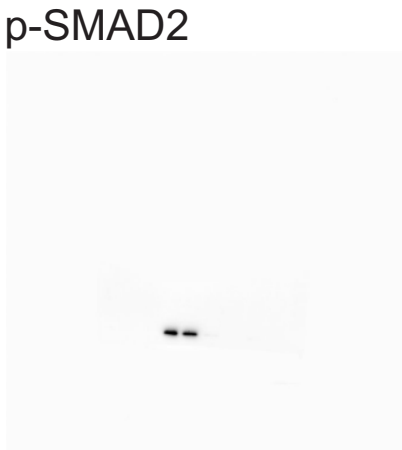

repeat 3

actin

p-SMAD2

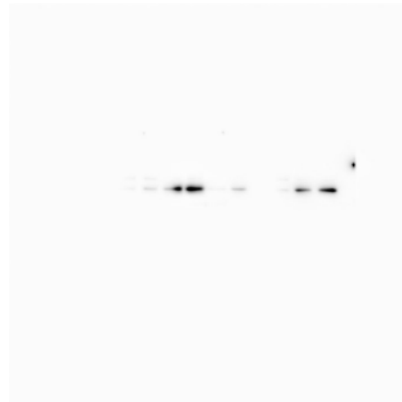

repeat 1

actin

p-SMAD3

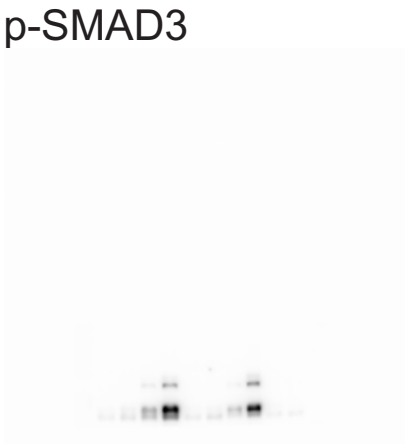

repeat 2

actin

p-SMAD3

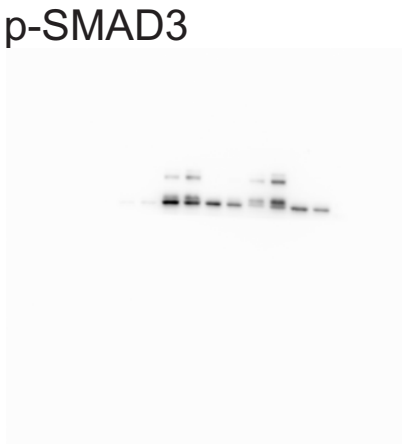

repeat 3

actin

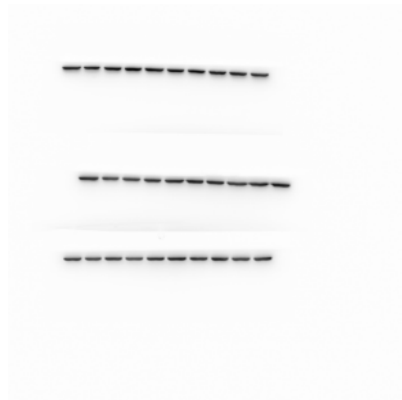

p-SMAD3

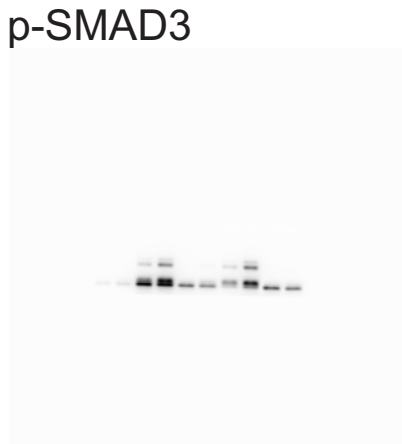

Western Blot Raw Data

Figure S7C

repeat 1

actin

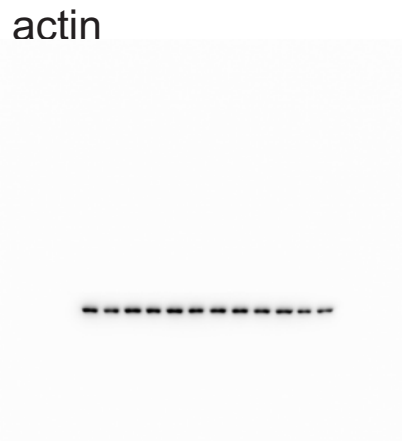

p-SMAD1/5/9

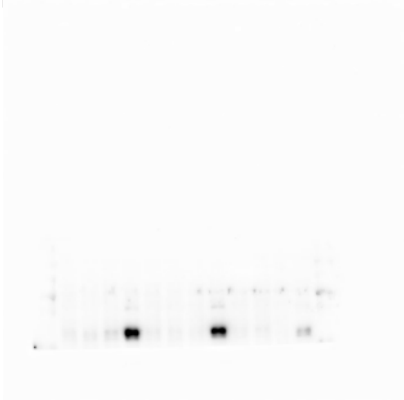

repeat 2

actin

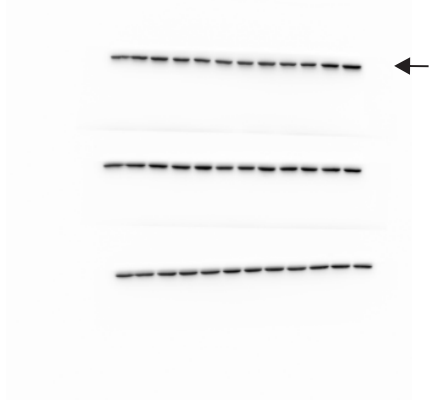

p-SMAD1/5/9

repeat 3

actin

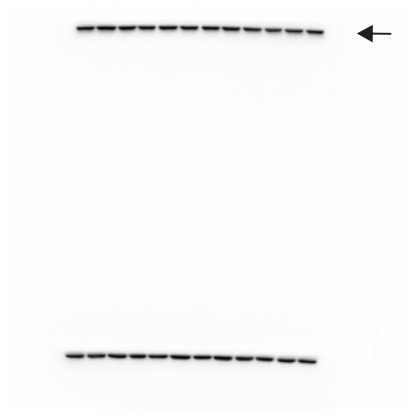

p-SMAD1/5/9

repeat 1

actin

p-SMAD2

repeat 2

actin

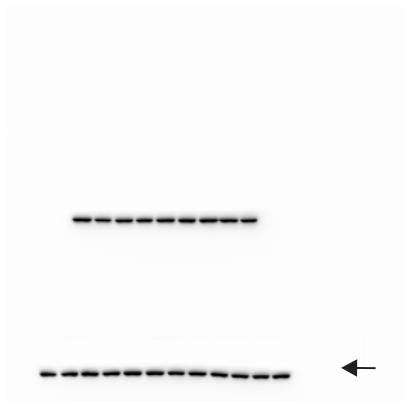

p-SMAD2

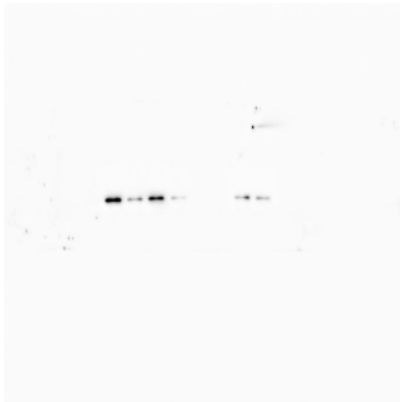

repeat 3

actin

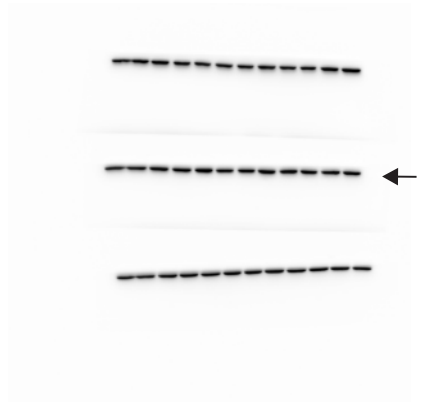

p-SMAD2

repeat 1

actin

p-SMAD3

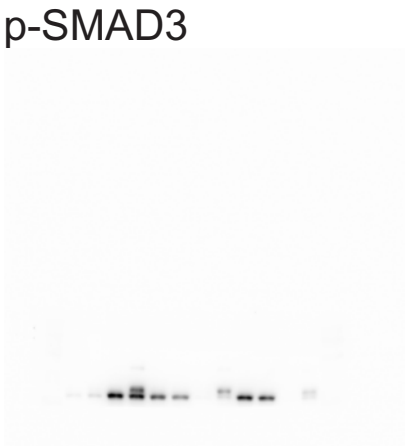

repeat 2

actin

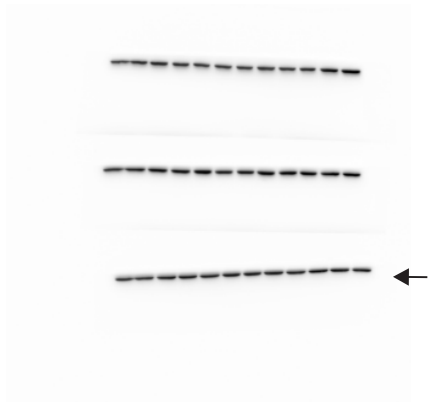

p-SMAD3

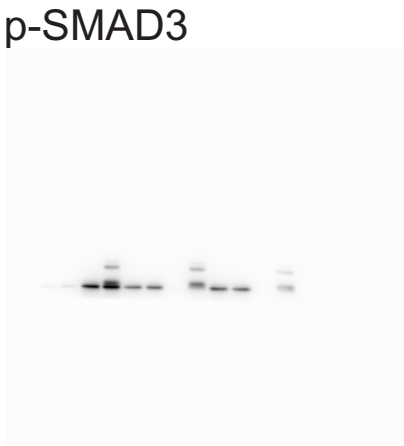

repeat 3

actin

p-SMAD3

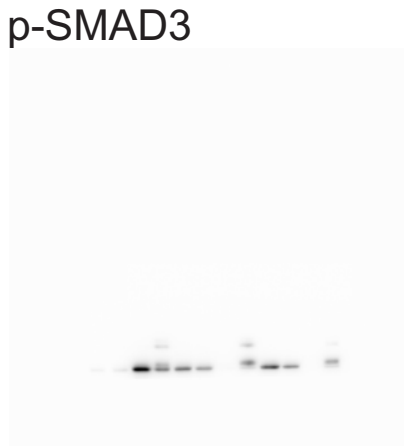

Supplement: Supplementary file 3 [file mmc3.pdf]
